# Macrocyclic Diterpenoids from *Euphorbia peplus* Possessing Activity Towards Autophagic Flux

**DOI:** 10.3390/ijms26010299

**Published:** 2024-12-31

**Authors:** Lu Chen, Lulan Liu, Yingyao Li, Shipeng Guan, Lingling Fan, Xujie Qin, Yingtong Di, Lei Tang, Rongcan Luo, Ying Yan

**Affiliations:** 1 State Key Laboratory of Functions and Applications of Medicinal Plants & College of Pharmacy, Guizhou Provincial Engineering Technology Research Center for Chemical Drug R&D, Guizhou Medical University, Guiyang 550014, China; 2 State Key Laboratory of Phytochemistry and Plant Resources in West China, Kunming Institute of Botany, Chinese Academy of Sciences, Kunming 650201, Chinadiyt@mail.kib.ac.cn (Y.D.); 3 Gansu Key Laboratory of Biomonitoring and Bioremediation for Environmental Pollution, and Key Laboratory of Cell Activities and Stress Adaptations, Ministry of Education, School of Life Sciences, Lanzhou University, Lanzhou 730000, China

**Keywords:** euphjatrophane, *Euphorbia peplus*, macrocyclic diterpenoid, autophagy, blood–brain barrier permeation

## Abstract

Euphjatrophanes H–L (**1**–**5**), four new jatrophane-type and one new lathyrane-type diterpenoid, were isolated from *Euphorbia peplus*, along with eight known diterpenoids (**6**–**13**). Their structures were established on the basis of extensive spectroscopic analysis and X-ray crystallographic experiments. All compounds were subjected to bioactivity evaluation using flow cytometry in autophagic flux assays with HM mCherry-GFP-LC3 cells, the human microglia cells which stably expressed the tandem monomeric mCherry-GFP-tagged LC3. Compounds **1**–**3**, **5**–**10**, and **12** significantly increase autophagic flux, and compounds **1** and **12** displayed relatively high BBB permeability, with log*Pe* values of −4.853 and −5.017, respectively. These findings indicated that jatrophane diterpenoids could serve as a valuable source for innovative autophagy inducers.

## 1. Introduction

Over the past decades, phytochemical research has always focused on the structurally diverse diterpenoids from the plants of the genus *Euphorbia*, resulting in the numerous diterpenoids featuring jatrophane, ingenane, pepluane, paraliane, and segetane scaffolds [1,2,3,4,5,6,7,8,9]. For instance, Resiniferatoxin, a diterpenoid of the daphnane type isolated from *E. resinifera*, is renowned for its ability to relieve neuropathic pain and is currently undergoing Phase I human clinical trials for the treatment of severe pain in cancer patients [10]. Euphodendroidin D, for example, is a jatrophane-type diterpenoid derived from *E. dendroides* that exhibits inhibitory effects on the transport activity of P-glycoprotein, an ABC transporter protein associated with multidrug resistance by lowering the intracellular drug concentration [11]. In 2012, the FDA approved ingenol 3-angelate, an ingenane-type diterpenoid obtained from *E. peplus*, for the treatment of actinic keratosis [12].

*E. peplus* Linn., a tiny annual weed originally from the Mediterranean coastline, was introduced to Yunnan province in China. Presently, it is found across the globe and has served as a traditional remedy for numerous years to address skin conditions, inflammatory disorders, asthma, diabetes, and various cancers [13]. Ingenane-type diterpenoids, specifically 20-deoxyingenol and its derivatives, have ever been reported by our research group. These compounds exhibited the ability to stimulate lysosome biogenesis and inhibit amyloid plaque formation in the brains of APP/PS1 mice [14]. This finding indicates their promising potential for the therapy of Alzheimer’s disease.

**Figure 1:**
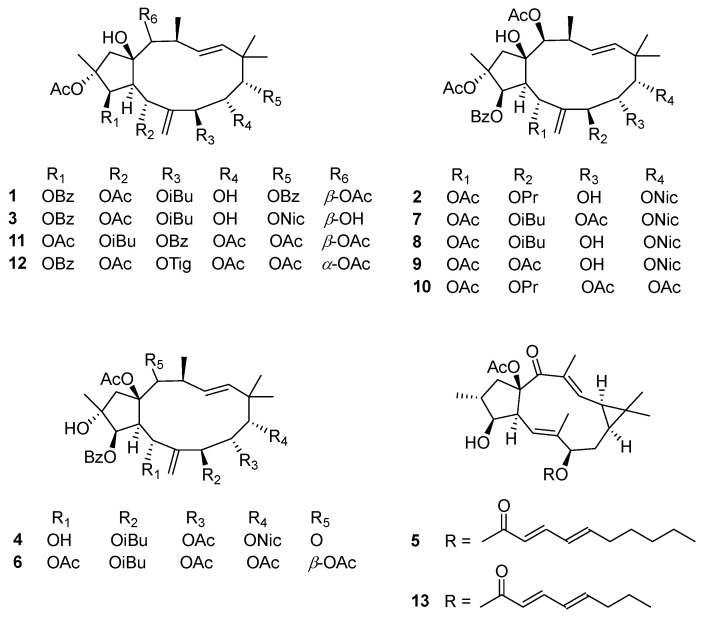
The chemical structures of compounds **1**–**13**.

The subsequent phytochemical studies of the plants of the genus *Euphorbia* led to the isolation of several novel diterpenoids with significant bioactivities. Among them, jatrophane-type diterpenes could significantly activate the lysosomal–autophagy pathway, which suggested that the *Euphorbia* diterpenoids could potentially be an important source for novel anti-Alzheimer’s disease (AD) drugs [15]. In our continuing efforts to uncover structurally novel diterpenes capable of inducing lysosomal biogenesis, four new jatrophane-type and one new lathyrane-type diterpenoid, named euphjatrophanes H–L (**1**–**5**), along with eight previously reported diterpenoids (**6**–**13**), were obtained from *E. peplus* (Figure 1). Their structures were elucidated using extensive NMR, X-ray crystallographic, and electronic circular dichroism (ECD) experiments. Moreover, compounds **1** and **12** displayed significant activity on autophagic flux and had relatively high BBB permeability. Herein, we reported the structural elucidation and biological evaluation of these compounds.

## 2. Results

### 2.1. Structure Elucidation

Compound **1** was obtained as a colorless cube crystal. The identification of its molecular formula of C_44_H_54_O_14_ was achieved based on its HRESIMS ion at *m*/*z* 829.3407 [M + Na]^+^ (calculated for 829.3406), corresponding to 18 degrees of unsaturation (DOUs). Its ^1^H and ^13^C-DEPT NMR spectra indicated the presence of three acetyls [*δ*_H_ 2.01, 2.10, and 2.18 (each 3H, s); *δ*_C_ 20.5, 21.1, 22.4, 168.5, 170.5, and 170.8], two benzoyloxy groups [*δ*_H_ 7.42, 7.45, 7.55, and 7.58 (each 2H), 7.55, and 7.58 (each 1H); *δ*_C_ 128.4 × 2, 128.5 × 2, 129.7 × 2, 130.3 × 2, 129.0, 130.1, 133.1, 133.6, 164.8, and 167.2], and one isobutyryloxy group [*δ*_H_ 1.92 (m, 1H), 0.91, 0.41 (each 3H); *δ*_C_ 17.2, 19.1, 33.5, and 174.2]. Apart from these acyl groups, the ^13^C NMR also showed twenty carbon signals, including four methyls (*δ*_C_ 23.2, 23.3, 23.6, and 27.5), one sp^3^ methylene (*δ*_C_ 50.0), eight sp^3^ methines with six oxygenated (*δ*_C_ 37.5, 44.8, 68.2, 70.4, 71.9, 79.3, 80.5, and 86.4), three sp^3^ non-proton-bearing carbons with two oxygenated (*δ*_C_ 40.4, 84.3, and 88.5), an exocyclic double bond [*δ*_H_ 4.45, and 4.83 (each, br s, 1H); *δ*_C_ 109.0, and 144.3], and a trans-disubstituted double bond [*δ*_H_ 6.15 (d, *J* = 16.0 Hz), and 5.66 (dd, *J* = 9.6, 16.0 Hz); *δ*_C_ 131.2, and 134.0] (Table 1). Further signals were detected in the ^1^H NMR spectrum of **1** for the resonances of two exchangeable protons [*δ*_H_ 3.13, 3.48]. Among the 18 indices of hydrogen deficiency, 16 were attributed to 2 benzoyls, 3 acetyls, an isobutyryl, and 2 double bonds. The remaining two DOUs were assumed to be due to the presence of the bicyclic system of **1**. Therefore, it could be deduced to be a jatrophane polyester.

**Figure 2:**
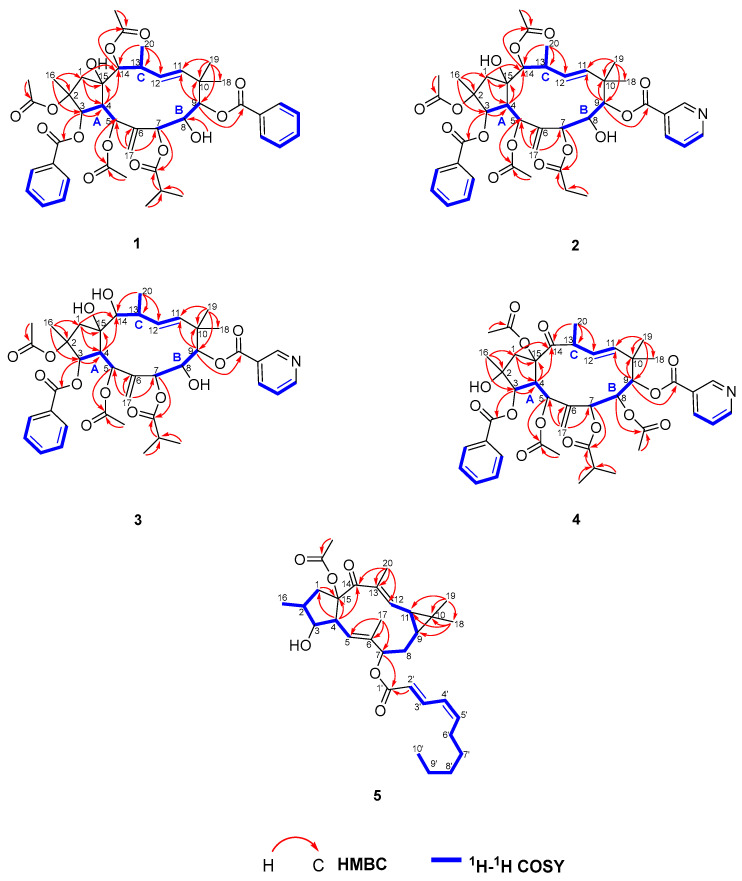
^1^H−^1^H COSY correlations and key HMBC of compounds **1**–**5**.

Analyses of the 2D NMR data enable the assembly of the aforementioned functionalities and then establish the structure of **1**. Three structural fragments, namely C-3/C-4/C-5 (fragment A), C-7/C-8/C-9 (fragment B), and C-11/C-12/C-13(Me-20)/C-14 (fragment C), were deduced from the analysis of 1H-1H COSY and HSQC spectra (Figure 2). HMBC correlations from H-4 (*δ*_H_ 3.66) to C-14 (*δ*_C_ 79.3) and C-15 (*δ*_C_ 84.3), and H-14 (*δ*_H_ 5.14) to C-4 (*δ*_C_ 44.8) and C-15 (*δ*_C_ 84.3) suggested that fragments A and C were linked through C-15. Fragments B and C were inferred to be interconnected via gem-dimethyl and carbonyl groups, supported by HMBC correlations from H3-19 (*δ*_H_ 1.36)/H3-18 (*δ*_H_ 1.07) to C-9 (*δ*_C_ 86.4), C-10 (*δ*_C_ 40.4), and C-11 (*δ*_C_ 134.0); H-9 (*δ*_H_ 5.03) to C-10 (*δ*_C_ 40.4) and C-11 (*δ*_C_ 134.0). Cross peaks of H2-17 (*δ*_H_ 4.83, 4.45) to C-5 (*δ*_C_ 71.9), C-6 (*δ*_C_ 144.3), and C-7 (*δ*_C_ 68.2) in the HMBC spectrum indicated the connection between fragments A and B, and CH2-17 through the olefinic carbon C-6. In addition, The HMBC correlations from H2-1 (*δ*_H_ 2.82, 2.10) to C-4 (*δ*_C_ 44.8) and C-15 (*δ*_C_ 84.3), from Me-16 (*δ*_H_ 1.49) to C-1 (*δ*_C_ 50.0), C-2 (*δ*_C_ 88.5), and C-3 (*δ*_C_ 80.5) indicated the presence of a five-membered ring. Finally, cross peaks of H-2/the acetyl carbonyl (*δ*_C_ 170.8), H-3/the benzoyl carbonyl (*δ*_C_ 164.9), H-5/the acetyl carbonyl (*δ*_C_ 168.5), H-7/the isobutyryl carbonyl (*δ*_C_ 174.2), H-9/the benzoyl carbonyl (*δ*_C_ 167.2), H-14/the acetyl carbonyl (*δ*_C_ 170.5) in HMBC spectrum could be located the three acetoxy groups at C-2, C-5, and C-14, the two benzoyloxy groups at C-3, and C-9, and the isobutyryl group at C-7. The remaining two hydroxyls must be located at C-8, and C-15, respectively. Thus, the gross structures of **1** were established, as shown in Figure 2.

**Figure 3:**
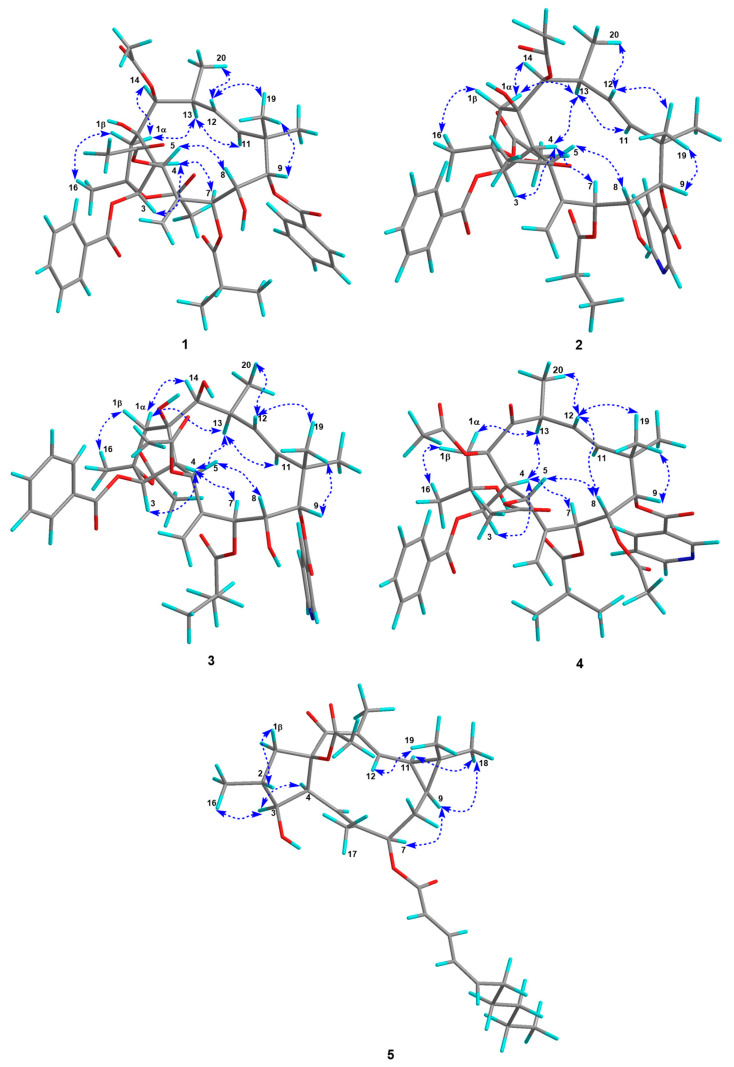
Selected ROESY correlations of compounds **1**–**5**.

**Figure 4:**
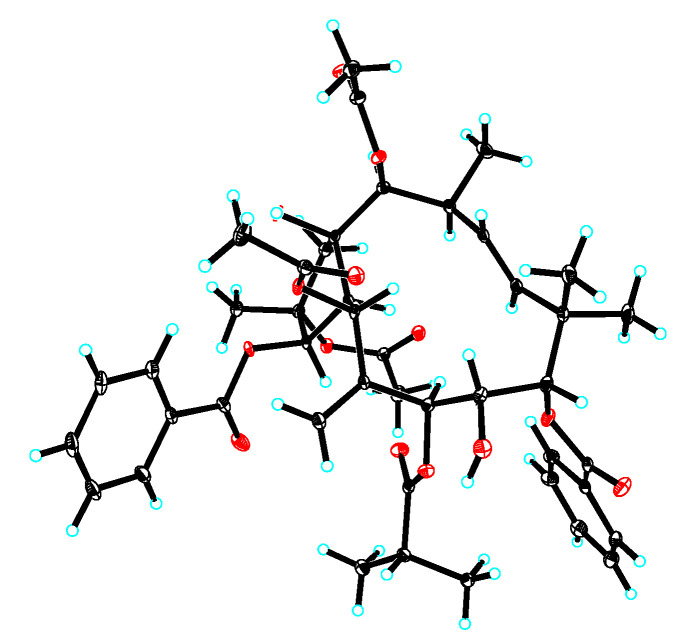
X-ray ORTEP drawing of euphjatrophane H (**1**).

The relative configuration of **1** was determined to be the same as that of the known compound, 2,5,14-triacetoxy-3-benzoyloxy-8,15-dihydroxy-7-isobutyroyloxy-9-nicotinoyloxyjatropha-6(17), 11E-diene [16], based on their similar ^1^H and ^13^C NMR data and ROESY correlations (Figure 3). A single-crystal X-ray diffraction by Cu Kα radiation [CCDC: 2408429, Flack parameter: 0.11(6)] allowed an unambiguous assignment of the absolute configuration for **1** as 2*R*,3*R*,4*S*,5*R*,7*S*,8*R*,9*S*,13*S*,14*S*,15*R* (Figure 4).

Euphjatrophane I (**2**), a white powder, was assigned a molecular formula of C_42_H_51_NO_14_, as deduced from its HRESIMS (*m*/*z* 794.3390 [M + H]^+^, calculated for C_42_H_52_NO_14_, 794.3382). The ^1^H and ^13^C NMR data (Table 1) of **2** showed a high similarity to euphjatrophane H (**1**), but one propionyloxy group (*δ*_H_ 1.92, 1.65, 0.60; *δ*_C_ 171.5, 27.1, 8.2) and one nicotinoyloxy group (*δ*_H_ 9.30, 8.82, 8.35, 7.40; *δ*_C_ 165.7, 154.1, 151.5, 137.4, 125.0, 123.3) occurred in compound **2** instead of the isobutyryloxy group and benzoyloxy group in the latter (Table 1). An extensive analysis of the HSQC, HMBC, and ^1^H-^1^H COSY spectra permitted a C-7 propionyloxy group and a C-9 nicotinoyloxy group in euphjatrophane I (**2**). In addition, the relative configuration of euphjatrophane B (**2**) was established as shown on the basis of the ROESY spectrum (Figure 3).

Compound **3** was obtained as a white powder, and the HRESIMS data showed an [M + H]^+^ ion at *m*/*z* 766.3435 (calculated for C_41_H_52_NO_13_, 766.3433), corresponding to the molecular formula C_41_H_51_NO_13_. The 1D NMR data (Table 1) of **3** showed a high similarity to those of euphjatrophane H (**1**) with the exception of the absence of the resonances from one benzoyloxy group and the presence of one nicotinoyloxy group (*δ*_H_ 9.28, 8.82, 8.35, 7.41; *δ*_C_ 165.9, 154.0, 151.5, 137.5, 125.1, 123.4). The HMBC correlations from H-9 (*δ*_H_ 5.06) to the nicotinoyl’s carbonyl (*δ*_C_ 165.9) revealed that the nicotinoyloxy group was located at C-9. On the grounds of the ROESY correlations analysis, the relative configuration of **3** was determined to be the same as that of euphjatrophane H (**1**). Specifically, the ROESY correlations of H-1α/OAc-2, H-3/H-4, H-4/H-7, H-4/H-13, H-7/H-11, and H-11/H_3_-18, as well as H-5/H-8, H-8/H-12, H-8/H_3_-19, and H-9/H_3_-19, were observed.

**Figure 5:**
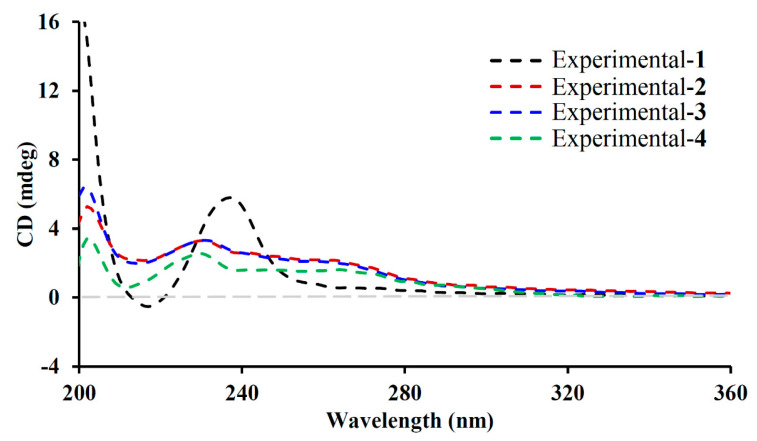
Measured ECD spectra of compounds **1**–**4**.

Euphjatrophane K (**4**) was also obtained as an amorphous white solid and possessed the molecular formula C_41_H_49_NO_13_, based on its HRESIMS ion at *m*/*z* 786.3112 [M + Na]^+^ (calculated for C_41_H_49_NO_13_Na, 786.3102). The ^1^H and ^13^C NMR data of euphjatrophane K (4) were closely related to those of the isolated euphopeplin A (**7**), except for one carbonyl group occurring in euphjatrophane K (**4**) instead of the acetoxy group at C-14 in euphopeplin A (Table 2), as evidenced by the crucial HMBC correlation from H_2_-1/H-4/H_3_-20 to the ketone carbonyl (*δ*_C_ 212.5) (Figure 2). The remaining relative configurations of euphjatrophane K (**4**) were the same as those of euphopeplin A (**7**) based on their similar 1D NMR shifts and ROESY data. Furthermore, the similar electronic circular dichroism (ECD) spectra of compounds **1**−**4** (Figure 5) indicated they shared the same absolute configuration.

Euphjatrophane L (**5**) was isolated as a yellow oil, and its molecular formula was deduced as C_32_H_46_O_6_ by the observed HRESIMS ion at *m*/*z* 527.3372 [M + H]^+^, calculated for C_32_H_47_O_6_, 527.3367, indicating 10 DOUs. The ^1^H and ^13^C NMR data (Table 2) of **5** indicated the presence of one acetyl (*δ*_H_ 2.04; *δ*_C_ 169.7, 21.7), one (2*E*,4*Z*)-deca-2,4-dienoyl (*δ*_H_ 6.58; *δ*_C_ 194.7,145.3, 133.1), one exocyclic double bond, and one trisubstituted α,β-unsaturated ketone moiety (*δ*_H_ 7.63, 6.12, 5.89, 5.88, 2.26, 1.48, 1.42, 1.30, 1.28, 0.88; *δ*_C_ 166.9, 142.3, 140.2, 126.3, 120.8, 31.4, 29.1, 28.3, 22.5, 14.0). Among the ten DOUs, seven were attributed to one acetyl, one (2*E*,4*Z*)-deca-2,4-dienoyl, two double bonds, and one ketone carbonyl. The remaining three DOUs were assumed to be due to the presence of the tricyclic system of **1**. Moreover, resonances [*δ*_H_ 1.14 (H-9), 1.29 (H-11), 1.19 (H_3_-18), 1.09 (H_3_-19), and *δ*_C_ 24.3 (C-10), as well as two tertiary methyl groups at *δ*_H_ 1.56 (H_3_-17) and 1.84 (H_3_-20)], were observed which are characteristic signals for a gem-dimethyl cyclopropane moiety. Therefore, it could be deduced to be a lathyrane-type polyester with a 5/11/3-tricyclinc skeleton (Table 2).

A comparison of the ^1^H and ^13^C NMR spectra of compound **5** with euphohelioscopin B suggested that they have the same lathyrane ring system [17], except for one (2*E*,4*Z*)-2,4-dienoyloxy group at C-7 occurring in compound **5** instead of the 4,5-dihydroxy-2*E*-octenoyloxy moiety in euphohelioscopin B. This finding was further supported by the HMBC cross-peak from H-7 (*δ*_H_ 4.89) to the carbonyl carbon (*δ*_C_ 166.9). In addition, the relative configuration at the remaining chiral centers of euphjatrophane L (**5**) would be analogous to those of the latter on the basis of ^13^C NMR shifts and NOE data.

Furthermore, nine previously identified diterpenoids, named euphpepluones G (**6**) [15], euphopeplin A (**7**) [6], 2,5,14-triacetoxy-3-benzoyloxy-8,15-dihydroxy-7-isobutyroyloxy-9-nicotinoyloxyjatropha-6(17),11E-diene (**8**) [16], 2, 5,7,14-tetraacetoxy-3-benzoyloxy-8,15-dihydroxy9-nicotinoyloxyjatropha-6(17),11E-diene (**9**) [16], 2α,5α,8α,9α,14β-Pentaacetoxy-3β-benzoyloxy-7-propionyloxyjatropha-6(17),11E-dien-15β-ol (**10**) [18], pepluanin C (**11**) [19], euphpepluone F (**12**) [20], and Euphohelioscopin A (**13**) [17], were characterized through a comparative analysis of their MS and NMR data with the established literature values.

### 2.2. X-Ray Diffraction Data Analysis

A suitable crystal of **1** was selected and recorded on a Bruker APEX DUO diffractometer equipped with an APEX II CCD using Cu Kα radiation. Cell refinement and data reduction were performed with Bruker SAINT Software Version 6.45. The structures were solved by direct methods using SHELXS-97, and refinements were performed with SHELXL-97 using full-matrix least-squares, with anisotropic displacement parameters for all the non-hydrogen atoms; hydrogen atoms were placed in calculated positions and refined using a riding model. Molecular graphics were computed with PLATON. The crystallographic data were deposited at the Cambridge Crystallographic Data Center with the deposition number CCDC 2408429.

Crystal data for **1**: C_44_H_54_O_14_·H_2_O, *M* = 824.88, *a* = 10.9602(5) Å, *b* = 15.0409(7) Å, *c* = 25.4717(11) Å, *α* = 90°, *β* = 90°, *γ* = 90°, *V* = 4199.0(3) Å3, *T* = 100.(2) K, space group P212121, *Z* = 4, *μ*(Cu Kα) = 0.815 mm^−1^, 39,527 reflections measured, and 7952 independent reflections (*Rint* = 0.1415). The final *R_1_* values were 0.0531 (*I* > 2σ(*I*)). The final *wR(F^2^)* values were 0.1207 (*I* > 2σ(*I*)). The final *R_1_* values were 0.0681 (all data). The final *wR(F^2^)* values were 0.1252 (all data). The goodness of fit on *F^2^* was 1.119, and the Flack parameter = 0.11(6).

### 2.3. Bioactivity of the Compounds Towards Autophagic Flux

**Figure 6:**
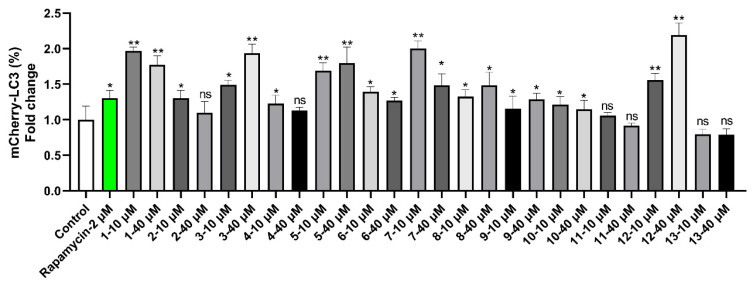
Flow cytometry analysis shows the bioactivity of compounds **1**–**13** towards autophagic flux. Rapamycin (2 μM) was used as the control. The results are presented as the mean ± SD (*n* = 3): * *p* < 0.05, ** *p* < 0.01, ns, not significant, compared to control.

The bioactivity of all the compounds towards autophagic flux was evaluated using HM mCherry-GFP-LC3 cells by flow cytometry (Figure 6). It is noteworthy that our findings revealed that compounds **1**−**3**, **5**−**10**, and **12** significantly increased autophagic flux, particularly for compounds **1** and **12**, which exhibited the most promising activity in this regard. In contrast, compounds **2**, **4**, **11**, and **13** did not significantly influence autophagic flux and, in fact, exhibited no significant effect on autophagic flux. The structure–activity relationships of these jatrophane diterpenoids and lathyrane diterpenoids were briefly analyzed. Compound **12** exhibited significantly activated autophagic flux compared to compounds **2**, **4**, and **11**, which indicated that ring A, containing ketone functionality and an acetyl group at C-14 in *R* configuration, completely lost activity. When considering **1**, **2**, **4**, and **11** specifically, compound **1** showed a relatively more potent activated autophagic flux, suggesting that the presence of both an isobutyryl group at C-7 and a benzoyl group at C-9 might contribute to the resultant activated autophagic flux. A comparison of the activated autophagic flux activity of **6** with those of **7** indicated that the presence of an acetyl group at C-2 may contribute to the higher activity. The activated autophagic flux of **8** and **9** suggested that the isobutyryl group at C-7 might cause a decrease in activity. Regarding **5** and **13** specifically, compound **5** demonstrated relatively more potent activated autophagic flux than **13**, implying that the presence of a (2*E*,4*Z*)-2,4-decadienoic acid ester group at C-7 might contribute to the observed activated activities.

### 2.4. Blood–Brain Barrier (BBB) Permeation

In the development of effective therapeutic drug candidates for neurodegenerative diseases, assessing the permeability of molecules across the blood–brain barrier (BBB) plays an important role. We employed a parallel artificial membrane permeability assay tailored for the blood–brain barrier (PAMPA-BBB) to ascertain the brain penetration capability of compounds **1** and **12**, utilizing three commercially available drugs, carbamazepine, propranolol, and furosemide, as references. The result of the PAMPA-BBB assay (Table 3) indicates that compounds **1** and **12** have excellent BBB permeability, with log*Pe* values of −4.853 and −5.017, respectively.

## 3. Discussion

Previous research results from our research group indicate that jatrophane diterpenes can activate autophagy and induce lysosomal biogenesis. In vivo studies have shown that these compounds can clear amyloid-β in the brains of mice, suggesting that these compounds are an important source for the treatments for neurodegenerative diseases [14,15]. To identify more Euphorbia diterpenoids, which could induce and activate autophagy and lysosomal biogenesis from the plants of the genus *Euphorbia*, we studied the chemical composition of *E. peplus*. Jatrophane diterpenoids predominantly exist as polyesters, sharing a key 5/12 bicyclic pentadecane framework. Additional structural variations involve a range of oxygenated substituents and stereochemical attributes within their inherently flexible 5/12 bicyclic scaffold. Consequently, X-ray single crystal diffraction, particularly utilizing Cu Kα radiation, is frequently employed in structural analysis to precisely delineate their structures. Notably, the absolute configuration of compound **1** was unequivocally established through single-crystal X-ray diffraction analysis. Furthermore, the comparison of electronic circular dichroism (ECD) spectra for compounds **1**–**4** suggests they had the same absolute configuration.

Autophagy represents a conserved intracellular degradation pathway whereby unwanted or damaged organelles and misfolded proteins are delivered to lysosomes for breakdown. Given the pivotal role of autophagy defects in the pathogenesis of AD, the upregulation of autophagy has been proposed as a potential strategy for the prevention of this disease. The present study was designed to test the effect of compounds **1** and **12** on autophagy. The evaluation of small molecule permeability through the blood–brain barrier (BBB) is crucial for the development of effective drugs targeting neurodegenerative diseases. To investigate the ability of these jatrophane diterpenoids to penetrate the blood–brain barrier, a future study will be conducted to test the ability of compounds **1** and **12** to activate autophagy in vivo and in vitro in order to treat Alzheimer’s disease. The present study also measured the brain penetration capability of compounds **1** and **12** using a tailored assay for the blood–brain barrier (PAMPA-BBB), which will provide a theoretical basis for conducting subsequent animal experiments.

In conclusion, four new jatrophane-type and one new lathyrane-type diterpenoid were isolated from the plants of *Euphorbia peplus*, along with eight known diterpenoids. The structures of these diterpenoids were elucidated through comprehensive NMR investigations, HRESIMS, and single-crystal X-ray diffraction analyses. Notably, Compounds **1**–**3**, **5**–**10**, and **12** significantly increase autophagic flux, and **1** and **12** exhibited the most promising activity in this regard. Moreover, PAMPA-BBB assays indicated that compounds **1** and **12** had the ability to penetrate the blood–brain barrier (BBB) through the process of transcellular passive diffusion. This study enriched the structural diversity of diterpenes and provided promising target molecules for the treatment of neurodegenerative diseases.

## 4. Materials and Methods

### 4.1. General Experimental Procedures

Optical rotations were measured on a Jasco P-1020 polarimeter (Jasco, Tokyo, Japan). UV spectra were obtained using a Shimadzu UV-2401A (Shanghai, China). IR spectra were recorded on a Bruker Tensor-27 infrared spectrophotometer with KBr disks. NMR spectra were collected on a Bruker Avance III 600 spectrometer (Bruker, Karlsruhe, Germany) using TMS as an internal standard. Waters Autospec Premier P776 mass spectrometers (Waters, Milford, MA, USA) were employed for HRESIMS measurements. Semipreparative HPLC was performed on an Agilent 1200 liquid chromatograph (Aglient, Santa Clara, CA, USA) with a Waters X-Bridge C18 (4.6 × 250 mm) column. Fractions were monitored by thin-layer chromatography (TLC, HSGF_254_, Yantai Jiangyou silica Gel Development Co., Ltd., Yantai, China) and visualized by spraying with a Vanillin chromogenic agent (Chongqi, China). For column chromatography (CC), silica gel (90–150 μm, Qingdao Marine Chemical Ltd., Qingdao, China), Sephadex LH-20 gel (40–70 μm, Amersham Pharmacia Biotech AB, Uppsala, Sweden), and Lichroprep RP-C18 gel (40–63 μm, Merck, Darmstadt, Germany) were utilized. TLC spots were visualized under UV light and by dipping into 5% H_2_SO_4_ in EtOH, followed by heating.

### 4.2. Plant Material

The whole plants of *E. peplus*, identified by Prof. Shi-Jun Hu (Kunming Institute of Botany, Chinese Academy of Sciences), were collected in June 2022 from Guiyang, Guizhou Province, China. A voucher specimen (NODJ 20220713) has been deposited at the Laboratory of Guizhou Medical University.

### 4.3. Extraction and Isolation

The air-dried and powdered plant material (50.0 kg) was extracted with MeOH (3 × 40 L) under reflux three times (4, 3, and 3 h), respectively. The combined MeOH extracts were concentrated under a vacuum to obtain the crude residue (5.32 kg), which was suspended in water and then partitioned with EtOAc. The EtOAc portion (1728 g) was applied to a silica gel column using PE–EtOAc (50:1→5:1, *v*/*v*) and CH_2_Cl_2_–MeOH (20:1→1:1, *v*/*v*) to obtain seven fractions (Fr. 1–Fr. 6). Fr. 3 (163 g) was then separated over a MCI-gel column (MeOH–H_2_O, 5:5→10:0, *v*/*v*) to obtain five fractions (Fr. 3A–Fr. 3E). Fr. 3D (49 g) was chromatographed over a C18 silica gel column, eluted with a gradient of MeOH–H_2_O (6:4→10:0, *v*/*v*) to afford five subfractions (Fr. 3A1–Fr. 3A5). Fr. 3A3 (3.2 g) was applied to Sephadex LH-20 (MeOH) and semipreparative HPLC (70% MeOH in water) to obtain **6** (12.4 mg, *t*_R_ = 20.4 min) and **3** (14.2 mg, *t*_R_ = 25.6 min). Fr. 3A4 (2.9 g) was purified by silica gel column (CHCl_2_–MeOH, 15:1, *v*/*v*) and then Sephadex LH-20 (MeOH) to **1** (9.5 mg), **9** (12.7 mg), and **11** (16.2 mg). Compounds **4** (10.5 mg) and **12** (13.1 mg) were obtained from Fr. 3A5 (2.5 g) by a silica gel column eluted with CHCl_2_–MeOH (15:1, *v*/*v*) and Sephadex LH-20 (MeOH). Fr. 3E (37 g) was chromatographed on a C18 silica gel column eluted with a gradient of MeOH–H_2_O (6:4→10:0, *v*/*v*) to afford four subfractions (Fr. 3E1–Fr. 3E4). Fr. 3E2 (1.2 g) was applied to Sephadex LH-20 (MeOH) and semipreparative HPLC (73% MeOH in water) to obtain **5** (9.7 mg, *t*_R_ = 23.4 min) and **13** (11.5 mg, *t*_R_ = 26.5 min). Fr. 3E3 (1.5 g) was purified by silica gel column (CHCl_2_–MeOH, 10:1, *v*/*v*) and then Sephadex LH-20 (MeOH) to **7** (14.0 mg) and Fr. 3E3a. Fr. 3E3a (67 mg) was purified by semi-preparative HPLC (67% MeOH in water) to obtain **10** (10.9 mg, *t*_R_ = 22.0 min) and **12** (8.7 mg, *t*_R_ = 24.7 min). Fr. 3E4 (1.3 g) was applied to Sephadex LH-20 (MeOH) to yield **8** (19.8 mg).

Euphjatrophane H (**1**)

Colorless cube crystal (MeOH), mp 168.6–169.8 °C; ${\left[\alpha \right]}_{D}^{\mathrm{24}}$ +122.4 (c 0.1, MeOH); UV (MeOH) λ_max_ (log ε) 195 (5.05); CD (MeOH) λ(∆ε) 195 (−5.51), 200 (41.46), 216 (1.29), 237 (14.00); IR (KBr) *ν*_max_ 3589, 2972, 1729, 1601, 1451, 1376, 1234, 1112, 1024 cm^−1^; ^1^H and ^13^C NMR data, see Table 1; HRESIMS *m*/*z* 829.3407 [M + Na]^+^ (calculated for C_44_H_54_O_14_Na, 829.3406).

Euphjatrophane I (**2**)

White amorphous powder; ${\left[\alpha \right]}_{D}^{\mathrm{24}}$ +73.3 (c 0.132, MeOH); UV (MeOH) λ_max_ (log ε) 195 (4.80); CD (MeOH) λ(∆ε) 195 (0.56), 202 (9.65), 215 (3.90), 230 (3.30); IR (KBr) *ν*_max_ 3443, 2970, 1728, 1591, 1374, 1276, 1235, 1114, 1024 cm^−1^; ^1^H and ^13^C NMR data, see Table 1; HRESIMS *m*/*z* 794.3390 [M + H]^+^ (calculated for C_42_H_52_NO_14_, 794.3382).

Euphjatrophane J (**3**)

White amorphous powder; ${\left[\alpha \right]}_{D}^{\mathrm{24}}$ +68.3 (c 0.12, MeOH); UV (MeOH) λ_max_ (log ε) 195 (4.86); CD (MeOH) λ(∆ε) 195 (6.16), 201 (24.52), 214 (3.81), 230 (6.43); IR (KBr) *ν*_max_ 3425, 2928, 1727, 1660, 1373, 1278, 1115, 1068, 1024 cm^−1^; ^1^H and ^13^C NMR (CDCl_3_) data, see Table 1; HRESIMS *m*/*z* 766.3435 [M + H]^+^ (calculated for C_41_H_52_NO_13_, 766.3433).

Euphjatrophane K (**4**)

White amorphous powder; ${\left[\alpha \right]}_{D}^{\mathrm{24}}$ +163.1 (c 0.2, MeOH); UV (MeOH) λ_max_ (log ε) 195 (4.79); CD (MeOH) λ(∆ε) 195 (−12.57), 202 (7.20), 209 (1.45), 229 (5.22); IR (KBr) *ν*_max_ 3437, 2975, 1732, 1592, 1424, 1372, 1276, 1112, 1037 cm^−1^; ^1^H and ^13^C NMR (CD_3_OD) data, see Table 2; HRESIMS *m*/*z* 786.3112 [M + Na]^+^ (calculated for C_41_H_49_NO_13_Na, 786.3102).

Euphjatrophane L (**5**)

Yellow oil; ${\left[\alpha \right]}_{D}^{\mathrm{24}}$ +80.1 (c 0.15, MeOH); UV (MeOH) λ_max_ (log ε) 204 (4.31), 272 (4.39); CD (MeOH) λ(∆ε) 195 (46.25), 232 (−27.66), 272 (32.14); IR (KBr) *ν*_max_ 3438, 2928, 1740, 1622, 1373, 1266, 1064, 1006, 938 cm^−1^; ^1^H and ^13^C NMR (CDCl_3_) data, see Table 2; HRESIMS *m*/*z* 527.3372 [M + H]^+^ (calculated for C_32_H_47_O_6_, 527.3367).

### 4.4. Flow Cytometry Analysis

HM mCherry-GFP-LC3 cells, which stably express a triple fusion protein (red fluorescent protein (mCherry), green fluorescent protein (GFP), and the autophagy marker LC3, were created in our previous studies [14,15]. This triple-fusion protein is capable of directly reflecting the strength of autophagy flux. In the absence of autophagy, these cells exhibit yellow fluorescence due to the co-expression of red mCherry and green GFP. When autophagy is active, the process of autophagy leads to the fusion of autophagosomes and lysosomes, resulting in the formation of autolysosomes. The subsequent quenching of the acidic lysosomal environment causes the fluorescence of acid-sensitive GFP to be reduced, while mCherry remains unaffected. Consequently, the autolysosomes manifest red fluorescence. Consequently, the red fluorescence observed in the cells can be used as a marker for the formation of autolysosomes. The extent of red fluorescence can be used as an indicator of the efficiency of the flux from the autophagosome to the autolysosome [21]. The bioactivity of all compounds on autophagic flux was evaluated using HM mCherry-GFP-LC3 cells by flow cytometry [15]. In summary, the HM mCherryGFP-LC3 cells were cultured in 12-well plates for 24 h in DMEM (Dulbecco’s modified Eagle medium) supplemented with 10% fetal bovine serum (Gibco-BRL, 10099–141, Gaithersburg, MD, USA) in a 37 ◦C incubator with 5% CO_2_ at 95% humidity. Subsequently, the compounds were introduced directly to the culture medium, resulting in final concentrations of 10 µM and 40 µM. Rapamycin was used as a positive control. Twenty-four hours following the administration of the treatment, the cells were harvested and fixed using 4% paraformaldehyde (PFA). This was followed by a flow cytometry test, which was conducted in order to determine whether autophagic flux had been altered. The data were analyzed using the FlowJo software v10 (BD Biosciences, Franklin Lakes, NJ, USA).

### 4.5. PAMPA-BBB Assay

We used the parallel artificial membrane permeability assay for BBB (PAMPA-BBB) to determine the brain permeability of compounds **1** and **12** [22]. The compounds **1** and **12** were prepared with methyl alcohol at the concentration of 4 mg/mL. These were diluted with PBS (Phosphate Buffer Saline; pH = 7.4) to obtain the donor solutions 100 μg/mL PAMPA “sandwich”, consisting of a donor 96-well microliter plate and matching filter plate coated with 5 μL of 2% lecithin/dodecane. Compounds **1** and **12** were added to the wells (150 μL/well) of the pre-coated filter, and PBS was added to the wells (300 μL/well) of the receiver plate. The filter plate was coupled with the receiver plate, and the plate couple was incubated in the thermostat at 37 °C for 16 h. After incubation, the plates were separated, and the samples from each well of both the filter plate and the receiver plate were sucked out and analyzed by HPLC. See Supplementary Materials (Figures S1–S74).

### 4.6. Statistical Analysis

Data analyses were carried out by using GraphPad Prism 8 (GraphPad Software, Inc., La Jolla, CA, USA). The one-way ANOVA (analysis of variance) was performed using Dunnett’s post hoc test for comparison between the treated group and control group, and the values were expressed as mean ± standard deviation (SD). It is considered to be statistically significant if *p*-value < 0.05; *, *p* < 0.05; **, *p* < 0.01; ***, *p* < 0.001.

## Figures and Tables

**Table 1:** ^1^H (500 MHz) and ^13^C (125 MHz) NMR data of compounds **1**−**3** in CDCl_3_.

	1		2		3	
No.	*δ*_H_ (*J* in Hz)	*δ*_C_	*δ*_H_ (*J* in Hz)	*δ*_C_	*δ*_H_ (*J* in Hz)	*δ*_C_
1	2.82, d (14.3)	50.0	2.83, overlap	49.7	2.68, overlap	50.7
	2.10, d (14.3)		2.06, overlap		2.02, overlap	
2		88.5		88.5		88.0
3	5.92, d (5.8)	80.5	5.94, d (5.6)	80.7	5.88, d (5.5)	81.1
4	3.66, dd (5.8, 3.5)	44.8	3.75, dd (5.6, 3.6)	44.9	3.48, dd (9.6, 4.8)	44.5
5	5.79, d (3.5)	71.9	5.80, d (3.6)	72	5.79, d (3.8)	71.9
6		144.3		143.8		144.5
7	5.35, s	68.2	5.35, s	68.7	5.39, s	68.2
8	4.12, d (11.2)	70.4	4.15, d (10.8)	70.2	4.17, d (10.2)	70.2
9	5.03, s	86.4	5.09, s	86.6	5.06, s	87.1
10		40.4		40.4		40.4
11	6.15, d (16.0)	134.0	6.16, d (15.9)	133.9	6.03, br s	132.5
12	5.66, dd (16.0, 9.6)	131.2	5.68, dd (16.0, 9.6)	131.6	6.02, br s	132.3
13	2.87, m	37.5	2.85, m	37.6	2.66, m	37.7
14	5.14, s	79.3	5.14, s	79.4	3.66, s	78.6
15		84.3		84.4		85.5
16	1.49, s	23.3	1.48, s	23.6	1.36, s	23.3
17	4.83, br s	109.0	4.78, br s	109.1	4.86, br s	109.1
	4.45, br s		4.46, br s		4.49, br s	
18	1.07, s	27.5	1.07, s	27.3	1.06, s	27.6
19	1.36, s	23.2	1.36, s	23.1	1.51, s	22.7
20	1.17, d (7.0)	23.6	1.18, d (7.0)	23.6	1.25, d (6.9)	24.5
OAc-2						
C=O		170.8		171.1		170.8
	2.18, s	22.4	2.21, s	22.5	2.19, s	22.5
OAc-5						
C=O		168.5		168.6		168.5
	2.01, s	21.1	2.10, s	20.6	1.99, s	20.9
OAc-14						
C=O		170.5		170.5		
	2.10, s	20.5	2.03, s	21.1		
OBz-3						
C=O		164.9		164.9		164.7
1′		129.0		130		130.0
2′,6′	8.05, d (8.2)	129.7	8.07, d (8.2)	129.7	8.06, d (8.2)	129.6
3′,5′	7.42, t (7.8)	128.4	7.42, t (7.8)	128.4	7.46, t (7.8)	128.5
4′	7.55, t (7.4)	133.1	7.56, t (7.4)	133.2	7.58, t (7.4)	133.2
OiBu/OPrp-7						
C=O		174.2		171.5		174.4
1′′	1.92, m	33.5	1.92, m	27.1	1.97, m	33.6
2′′	0.91, d (7.0)	19.1	1.65, m		0.48, d (6.8)	17.4
3′′	0.41, d (6.8)	17.2	0.60, t (7.5)	8.2	0.94, d (7.0)	19.1
ONic/OBz-9						
C=O		167.2		165.7		165.9
1′′′		130.1	9.30, d (1.4)	151.5	9.28, d (1.8)	151.5
2′′′	8.09, d (8.2)	130.3		125		125.1
3′′′	7.45, m	128.5	8.35, dt (8.0, 1.6)	137.4	8.35, dt (8.0, 4.8)	137.5
4′′′	7.58, m	133.6	7.40, dd (8.0, 4.8)	123.3	7.41, dd (8.0, 4.8)	123.4
5′′′			8.82, dd (4.9, 1.8)	154.1	8.82, dd (4.9, 1.7)	154.0
OH-8	3.13 d (11.2)					
OH-15	3.48, s		3.65, s		3.98, s	

**Table 2:** ^1^H and ^13^C NMR data of compounds **4** and **5**.

	4 *^a^*		5 *^b^*	
No.	*δ*_H_ (*J* in Hz)	*δ*_C_	*δ*_H_ (*J* in Hz)	*δ*_C_
1	2.13, d (15.0)	50.0	2.61, dd (14.0, 10.2)	40.8
	3.21, d (15.0)		2.23, t (10.4)	
2		90.9	2.15, m	40.6
3	6.11, d (5.6)	79.7	3.83, dd (8.0, 4.8)	81.6
4	3.77, dd (5.8, 2.8)	48.9	2.62, dd (13.8, 3.6)	48.9
5	5.57, s	69.7	6.05, d (13.0)	121.2
6		146.3		143.4
7	4.36, s	69.8	4.89, dd (13.8, 3.6)	76.8
8	5.08, s	70.7	2.41, dt (16.4, 4.0)	33.2
9	5.14, s	82.2	1.83, m	30.2
10		41.7	1.14, m	24.3
11	6.24, d (15.8)	137.2	1.29, overlap	29.1
12	5.60, dd (15.8, 10.2)	132.1	6.58, d (13.6)	145.3
13	4.12, dd (9.6, 6.6)	45.4		133.1
14		212.5		194.7
15		90.9		96.3
16	1.52, s	22.1		18.3
17	4.96, br s	111.5	1.08, d (8.2)	18.8
	4.88, br s		1.56, s	
18	1.26, s	22.5	1.19, s	29.0
19	1.03, s	26	1.09, s	16.2
20	1.29, d (6.6)	19.2	1.84, s	12.2
OAc-8				
C=O		170.3		
	1.41, s	19.9		
OAc-15				
C=O		172		169.7
	2.30, s	22	2.04, s	21.7
OBz-3				
C=O		166.4		
1′		130.8		
2′,6′	8.08, d (8.2)	130.5		
3′,5’	7.47, t (7.8)	129.1		
4’	7.61, t (7.4)	133.9		
OiBu-7				
C=O		177.0		
1’′	2.56, m	34.6		
2’′	1.10, d (7.0)	18.9		
3’′	1.14, d (6.8)	18.7		
ONic-9				
C=O		164.5		
1′′′	9.22, d (2.2)	151.2		
2′′′		126.4		
3′′′	8.49, d (8.4)	138.8		
4′′′	7.65, dd (8.0, 4.8)	124.9		
5′′′	8.86, dd (5.0, 1.6)	154.4		
OR-7				
1′′′′				166.9
2′′′′			5.89, overlap	120.8
3′′′′			7,63, dd (18.4, 14.0)	140.2
4′′′′			6.12, t (13.4)	126.3
5′′′′			5.88, overlap	142.3
6′′′′			1.48, dd (13.8, 9.6)	29.1
			1.42, t (8.8)	
7′′′′			2.26, m	28.3
8′′′′			1.28, overlap	31.4
9′′′′			1.30, overlap	22.5
10′′′′			0.88, t (7.8)	14.0

*^a^* Data measured at 400 MHz and 100 MHz in CD_3_OD; *^b^* data measured at 600 MHz and 150 MHz in CDCl_3_.

**Table 3:** Permeability (log*Pe*) in the PAMPA-BBB assay for commercial drugs and compounds **1** and **12** with prediction of their penetration to the CNS.

Compounds	log*Pe*	Prediction	Compounds	log*Pe*	Prediction
Carbamazepine	−3.782 ± 0.816	CNS ++	**1**	−4.853 ± 0.076	CNS ++
Propranolol	−3.593 ± 0.089	CNS ++	**12**	−5.017 ± 0.122	CNS +
Furosemide	−6.367 ± 0.562	CNS −			

− is not detected in acceptor or log*Pe* < −6.0, + is log*Pe* > −6.0, ++ is log*Pe* > −5.0.

## Data Availability

The data underlying this study are available in the published article and its online Supplementary Materials.

## Supplementary Materials

The supporting information can be downloaded at https://www.mdpi.com/article/10.3390/ijms26010299/s1.
