# Supplementary material for: Macrocyclic Diterpenoids from Euphorbia peplus Possessing Activity Towards Autophagic Flux
Source: Int J Mol Sci. 2024 Dec 31;26(1):299. doi: 10.3390/ijms26010299 (PMC11719499; doi:10.3390/ijms26010299)
Supplement: Supplementary file 1 [file ijms-26-00299-s001.zip › Supplementary Information.pdf]

Macrocyclic Diterpenoids from *Euphorbia peplus* possessing activity towards autophagic flux

Lu Chen [1], Lulan Liu [1], Yingyao Li [2], Shipeng Guan [2], Lingling Fan [1], Xujie Qin [2], Yingtong Di [2], Lei Tang [1], Rongcan Luo [3,*] and Ying Yan [1,*]

Contents

Original spectroscopic data of new compounds 3
- **Figure S1. ^{1}H NMR (500 MHz) spectrum of 1 in $CDCl_3$.** 3
- **Figure S2. ^{13}C NMR (125 MHz) spectrum of 1 in $CDCl_3$.** 3
- **Figure S3. HSQC (500 MHz) spectrum of 1 in $CDCl_3$.** 4
- **Figure S4. HMBC (500 MHz) spectrum of 1 in $CDCl_3$.** 4
- **Figure S5. ^{1}H-^{1}H COSY (500 MHz) spectrum of 1 in $CDCl_3$.** 5
- **Figure S6. ROESY (500 MHz) spectrum of 1 in $CDCl_3$.** 5
- **Figure S7. HRESIMS spectrums of 1.** 6
- **Figure S8. IR spectrum of 1.** 7
- **Figure S9. UV spectrum of 1.** 7
- **Figure S10. CD spectrum of 1.** 8
- **Figure S11. ^{1}H NMR (500 MHz) spectrum of 2 in $CDCl_3$.** 8
- **Figure S12. ^{13}C NMR (125 MHz) spectrum of 2 in $CDCl_3$.** 9
- **Figure S13. HSQC (500 MHz) spectrum of 2 in $CDCl_3$.** 9
- **Figure S14. HMBC (500 MHz) spectrum of 2 in $CDCl_3$.** 10
- **Figure S15. ^{1}H-^{1}H COSY (500 MHz) spectrum of 2 in $CDCl_3$.** 10
- **Figure S16. ROESY (500 MHz) spectrum of 2 in $CDCl_3$.** 11
- **Figure S17. HRESIMS spectrums of 2.** 12
- **Figure S18. IR spectrum of 2.** 13
- **Figure S19. UV spectrum of 2.** 13
- **Figure S20. CD spectrum of 2.** 14
- **Figure S21. ^{1}H NMR (500 MHz) spectrum of 3 in $CDCl_3$.** 14
- **Figure S22. ^{13}C NMR (125 MHz) spectrum of 3 in $CDCl_3$.** 15
- **Figure S23. HSQC (500 MHz) spectrum of 3 in $CDCl_3$.** 15
- **Figure S24. HMBC (500 MHz) spectrum of 3 in $CDCl_3$.** 16
- **Figure S25. ^{1}H-^{1}H COSY (500 MHz) spectrum of 3 in $CDCl_3$.** 16
- **Figure S26. ROESY (500 MHz) spectrum of 3 in $CDCl_3$.** 17
- **Figure S27. HRESIMS spectrums of 3.** 18
- **Figure S28. IR spectrum of 3.** 19
- **Figure S29. UV spectrum of 3.** 19
- **Figure S30. CD spectrum of 3.** 20
- **Figure S31. ^{1}H NMR (400 MHz) spectrum of 4 in CD_3OD.** 20

Figure S32. ^{13}C **NMR (100 MHz) spectrum of 4 in** CD_3OD**.** 21
Figure S33. HSQC (400 MHz) spectrum of 4 in CD_3OD**.** 21
Figure S34. HMBC (400 MHz) spectrum of 4 in CD_3OD**.** 22
Figure S35. ^{1}H-^{1}H **COSY (400 MHz) spectrum of 4 in** CD_3OD**.** 22
Figure S36. ROESY (400 MHz) spectrum of 4 in CD_3OD**.** 23
Figure S37. HRESIMS spectrums of 4. 23
Figure S39. UV spectrum of 4. 24
Figure S40. CD spectrum of 4. 25
Figure S41. ^{1}H **NMR (600 MHz) spectrum of 5 in** $CDCl_3$**.** 25
Figure S42. ^{13}C **NMR (150 MHz) spectrum of 5 in** $CDCl_3$**.** 26
Figure S43. HSQC (600 MHz) spectrum of 5 in $CDCl_3$**.** 26
Figure S44. HMBC (600 MHz) spectrum of 5 in $CDCl_3$**.** 27
Figure S45. ^{1}H-^{1}H **COSY (600 MHz) spectrum of 5 in** $CDCl_3$**.** 27
Figure S46. ROESY (600 MHz) spectrum of 5 in $CDCl_3$**.** 28
Figure S47. HRESIMS spectrums of 5. 29
Figure S49. UV spectrum of 5. 30
Figure S50. CD spectrum of 5. 31
Original spectroscopic data of known compounds 31
Figure S51. ^{1}H **NMR (400 MHz) spectrum of 6 in** $CDCl_3$**.** 31
Figure S52. ^{13}C **NMR (100 MHz) spectrum of 6 in** $CDCl_3$**.** 32
Figure S53. ESIMS spectrum of 6. 32
Figure S54. ^{1}H **NMR (400 MHz) spectrum of 7 in** CD_3OD**.** 33
Figure S55. ^{13}C **NMR (100 MHz) spectrum of 7 in** CD_3OD**.** 33
Figure S56. ESIMS spectrum of 7. 34
Figure S57. ^{1}H **NMR (400 MHz) spectrum of 8 in** $CDCl_3$**.** 34
Figure S58. ^{13}C **NMR (100 MHz) spectrum of 8 in** $CDCl_3$**.** 35
Figure S59. ESIMS spectrum of 8. 35
Figure S60. ^{1}H **NMR (400 MHz) spectrum of 9 in** $CDCl_3$**.** 36
Figure S61. ^{13}C **NMR (100 MHz) spectrum of 9 in** $CDCl_3$**.** 36
Figure S62. ESIMS spectrum of 9. 37
Figure S63. ^{1}H **NMR (400 MHz) spectrum of 10 in** $CDCl_3$**.** 37
Figure S64. ^{13}C **NMR (100 MHz) spectrum of 10 in** $CDCl_3$**.** 38
Figure S65. ESIMS spectrum of 10. 38
Figure S66. ^{1}H **NMR (400 MHz) spectrum of 11 in** $CDCl_3$ 39
Figure S67. ^{13}C **NMR (100 MHz) spectrum of 11 in** $CDCl_3$ 39
Figure S68. ESIMS spectrum of 11. 40
Figure S69. ^{1}H **NMR (400 MHz) spectrum of 12 in** $CDCl_3$**.** 40
Figure S70. ^{13}C **NMR (100 MHz) spectrum of 12 in** $CDCl_3$**.** 41
Figure S71. ESIMS spectrum of 12. 41
Figure S72. ^{1}H **NMR (600 MHz) spectrum of 13 in** $CDCl_3$**.** 42
Figure S73. ^{13}C **NMR (150 MHz) spectrum of 13 in** $CDCl_3$**.** 42
Figure S74. ESIMS spectrum of 13. 43

Original spectroscopic data of new compounds

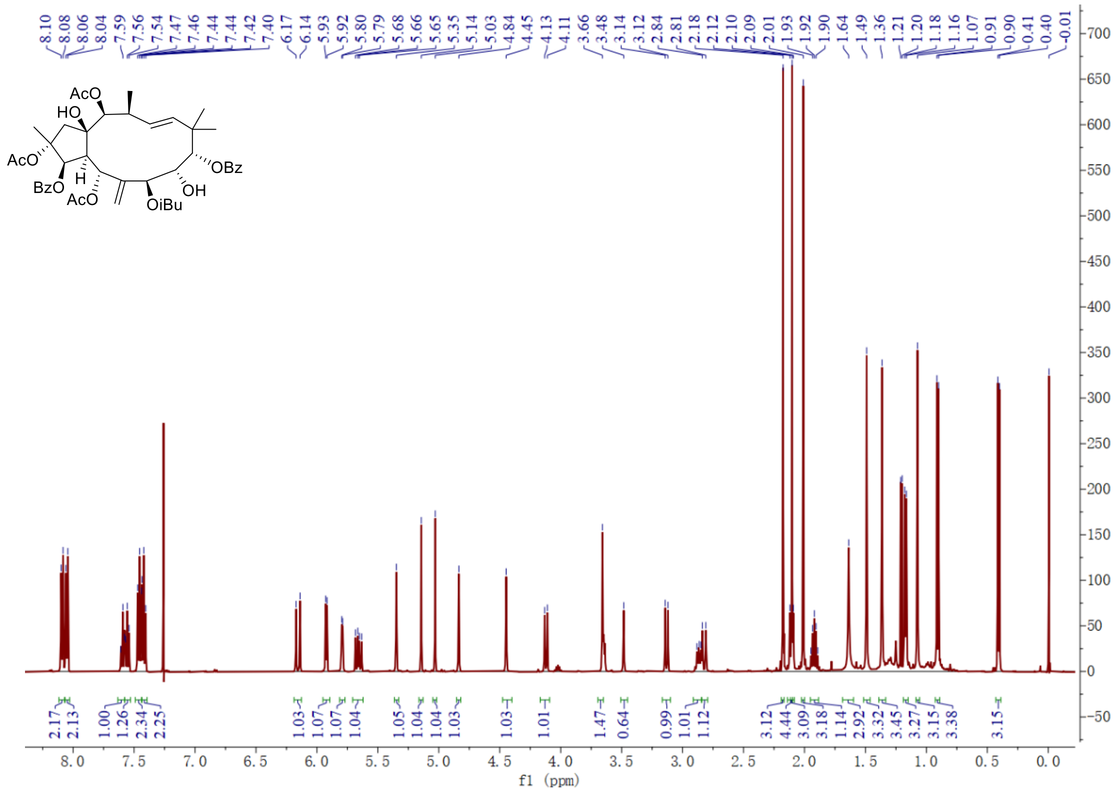

Figure S1. **^{1}H NMR (500 MHz) spectrum of 1 in $CDCl_3$.**

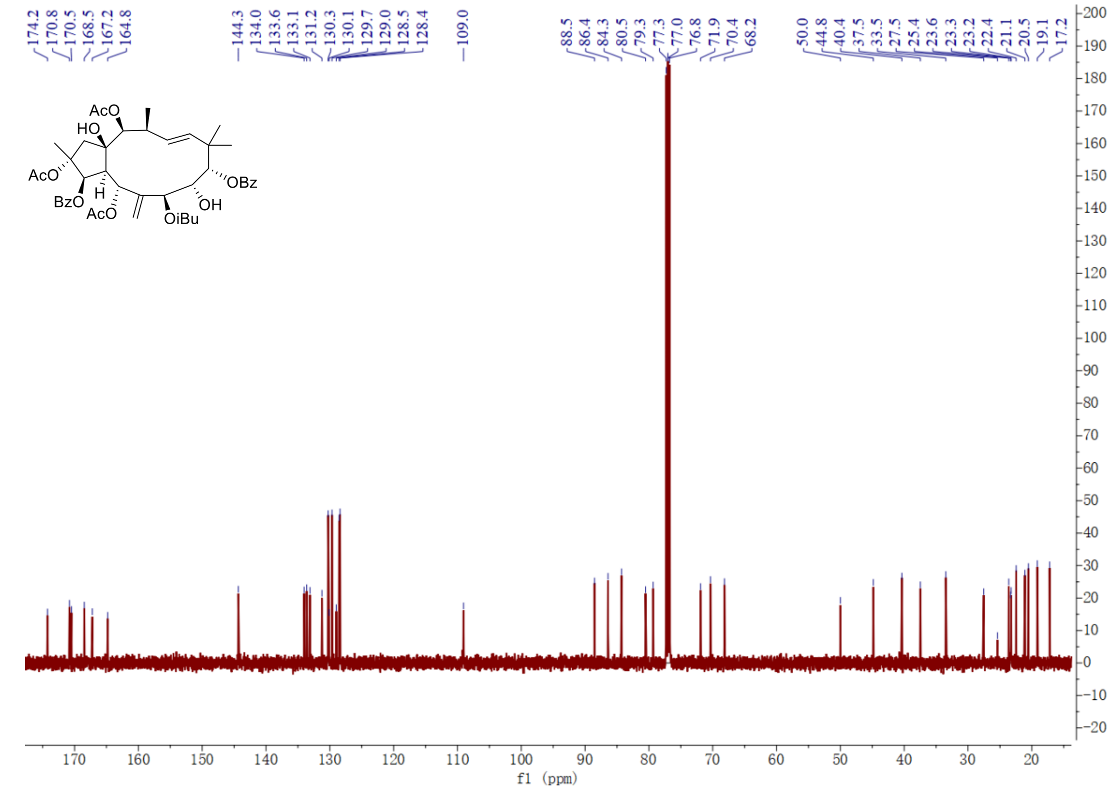

Figure S2. **^{13}C NMR (125 MHz) spectrum of 1 in $CDCl_3$.**

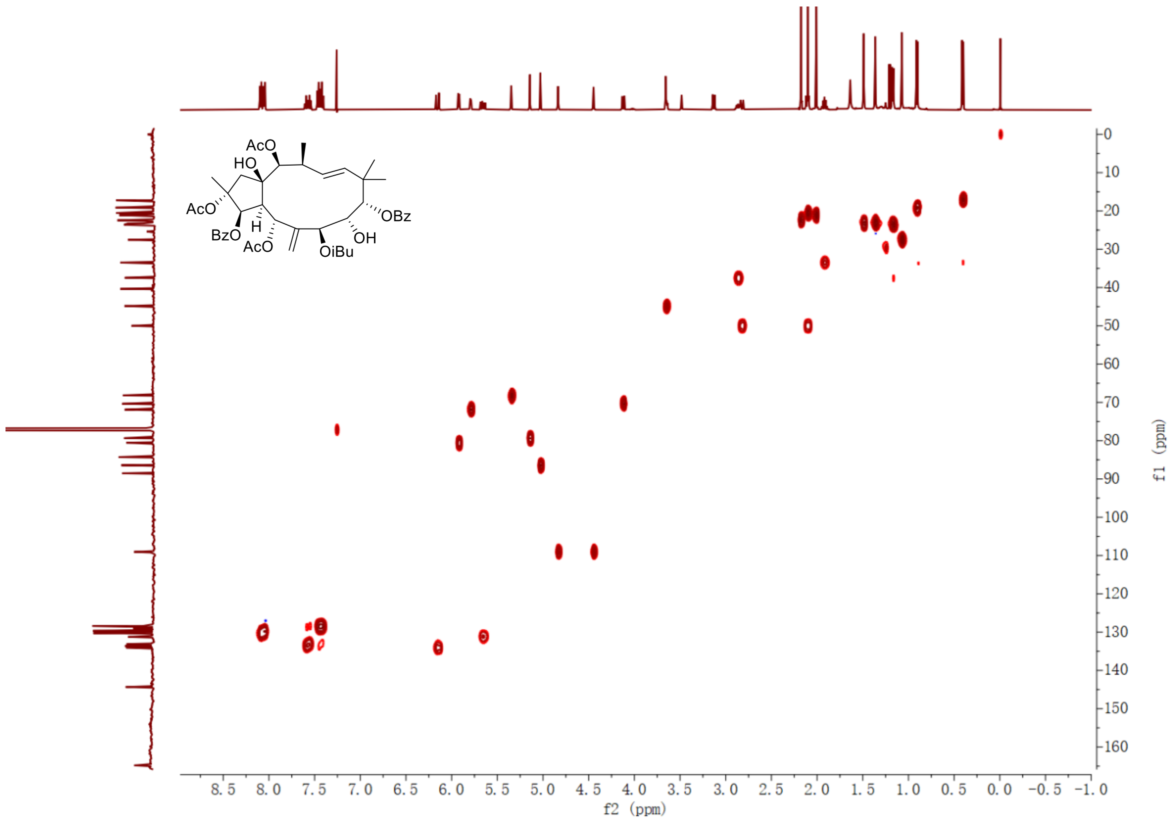

Figure S3. HSQC (500 MHz) spectrum of 1 in $CDCl_3$.

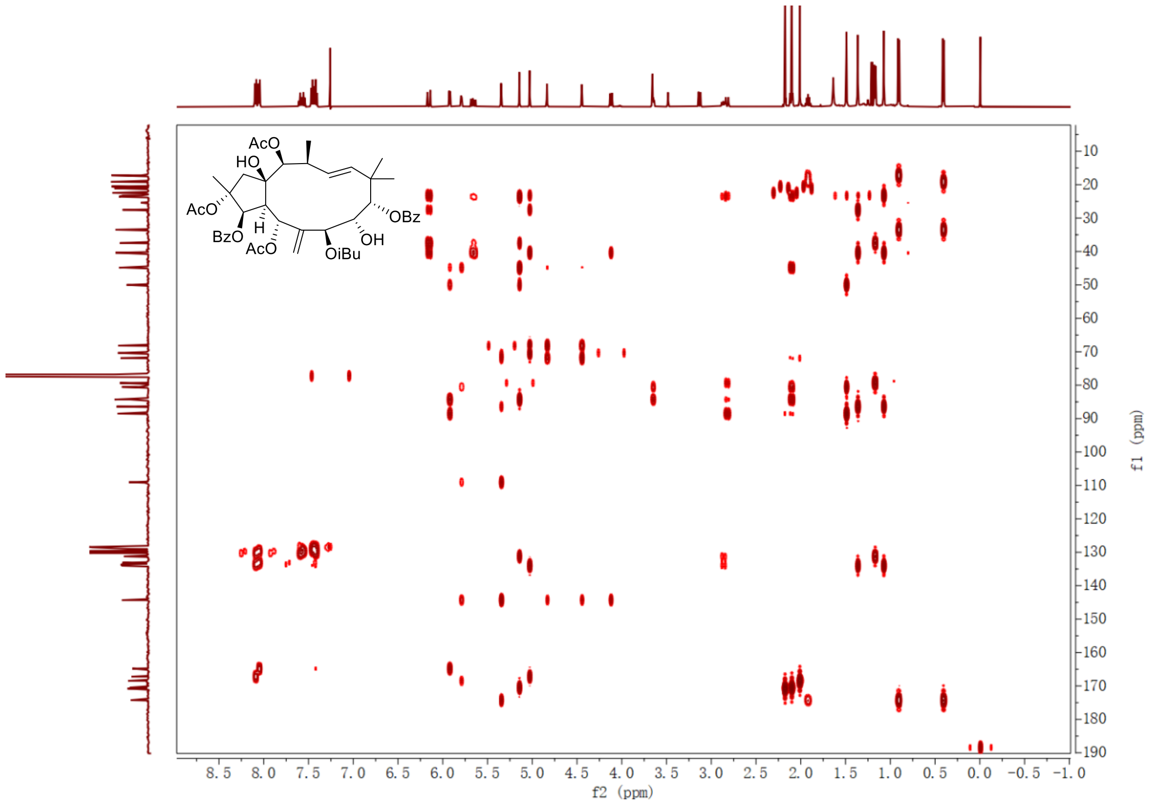

Figure S4. HMBC (500 MHz) spectrum of 1 in $CDCl_3$.

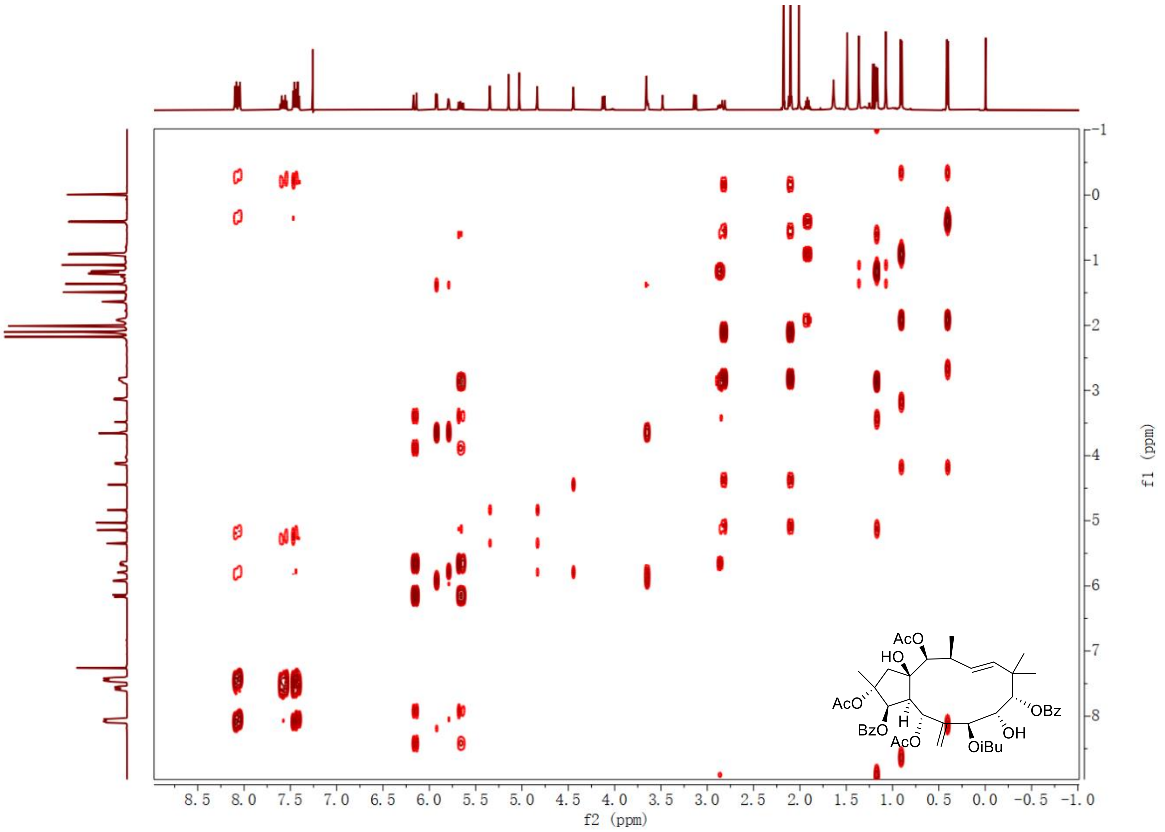

Figure S5. ^{1}H-^{1}H **COSY (500 MHz) spectrum of 1 in** $CDCl_3$**.**

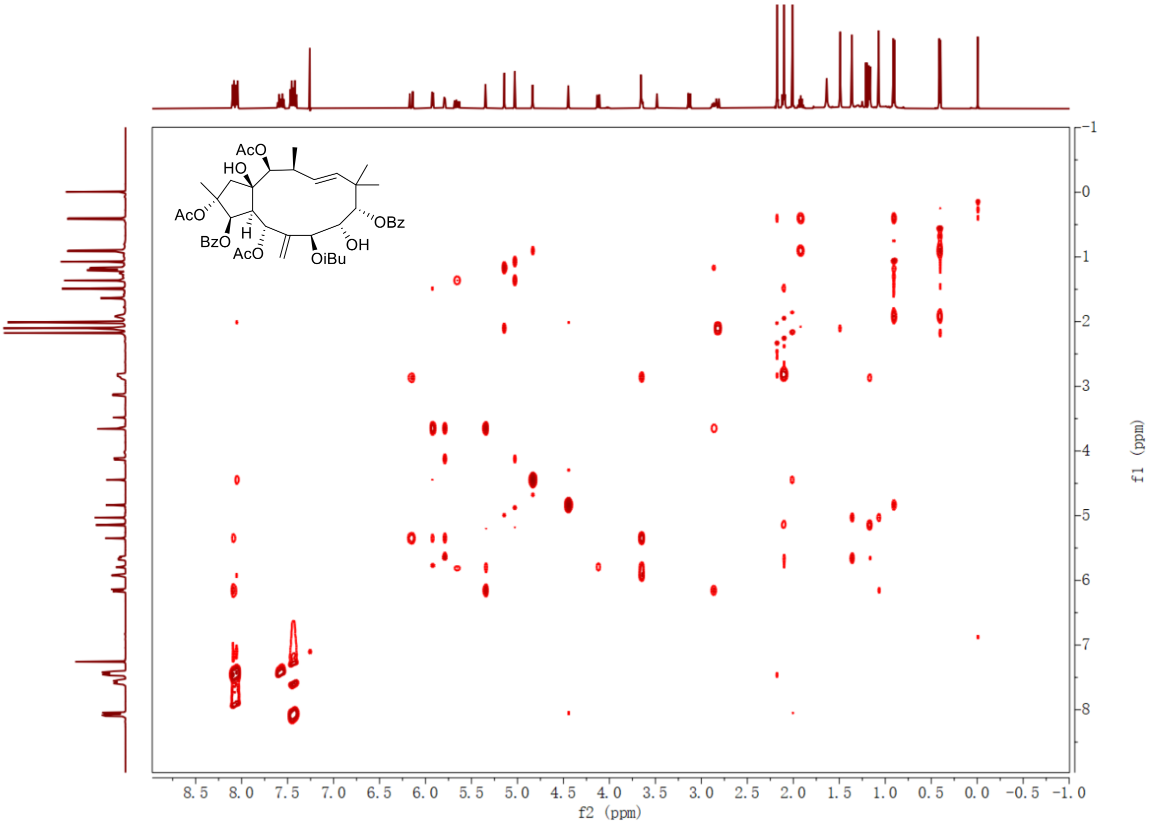

Figure S6. ROESY (500 MHz) spectrum of 1 in $CDCl_3$**.**

Qualitative Analysis Report

**Data Filename**	HLYY-3-4-2.d	**Sample Name**	HLYY-3-4-2
**Sample Type**	Sample	**Position**	P1-A7
**Instrument Name**	Instrument 1	**User Name**	
**Acq Method**	s.m	**Acquired Time**	12/11/2020 11:36:39 AM
**IRM Calibration Status**	Success	**DA Method**	Default.m
**Comment**			

Sample Group **Info.**
Acquisition SW 6200 series TOF/6500 series
Version Q-TOF B.05.01 (B5125.2)

User Spectra

Fragmentor Voltage	Collision Energy	Ionization Mode
175	0	ESI

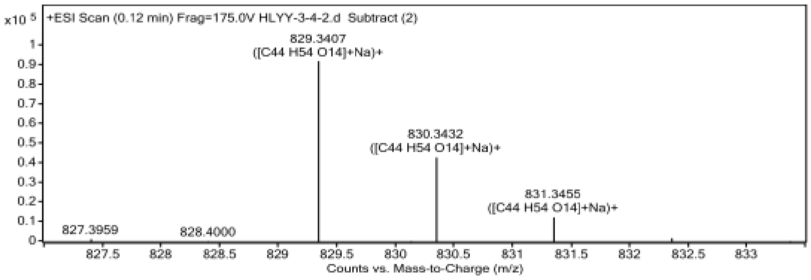

Peak List

m/z	z	Abund	Formula	Ion
823.3512		2632.56		
824.3842	1	45700.43		
825.3868	1	24055.78		
826.3905	1	6469.18		
829.3407	1	92032.35	C44 H54 O14	(M+Na)+
830.3432	1	43331.44	C44 H54 O14	(M+Na)+
831.3455	1	12691.46	C44 H54 O14	(M+Na)+
845.314	1	10581		
846.3163	1	5647.55		
852.4172	1	3094.16		

Formula Calculator Element Limits

Element	Min	Max
C	3	120
H	0	240
O	0	30

Formula Calculator Results

Formula	CalculatedMass	CalculatedMz	Mz	Diff. (mDa)	Diff. (ppm)	DBE
C44 H54 O14	806.3514	829.3406	829.3407	-0.10	-0.12	18.0000

Figure S7. HRESIMS spectrums of 1.

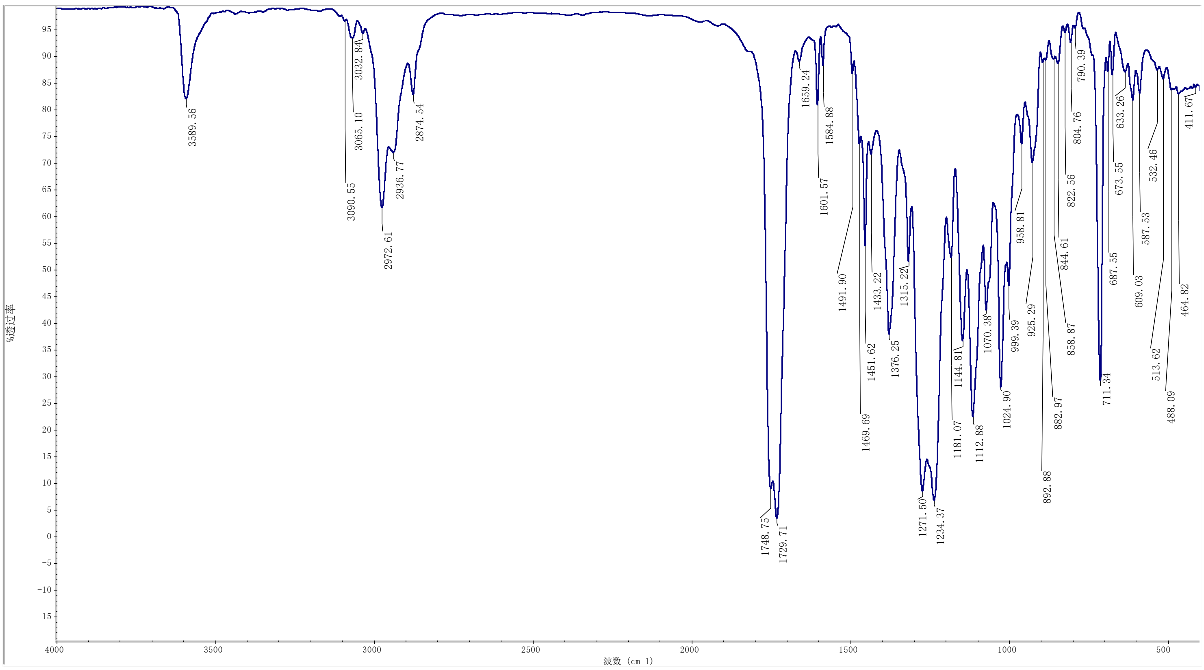

Figure S8. IR spectrum of 1.

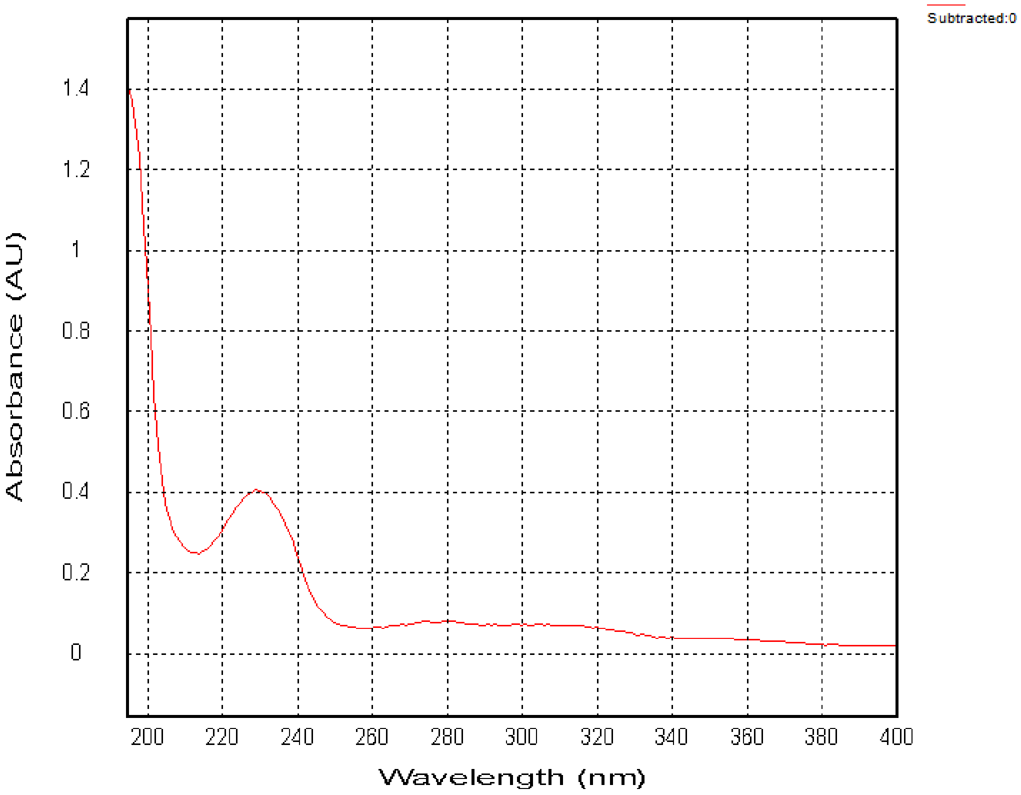

Figure S9. UV spectrum of 1.

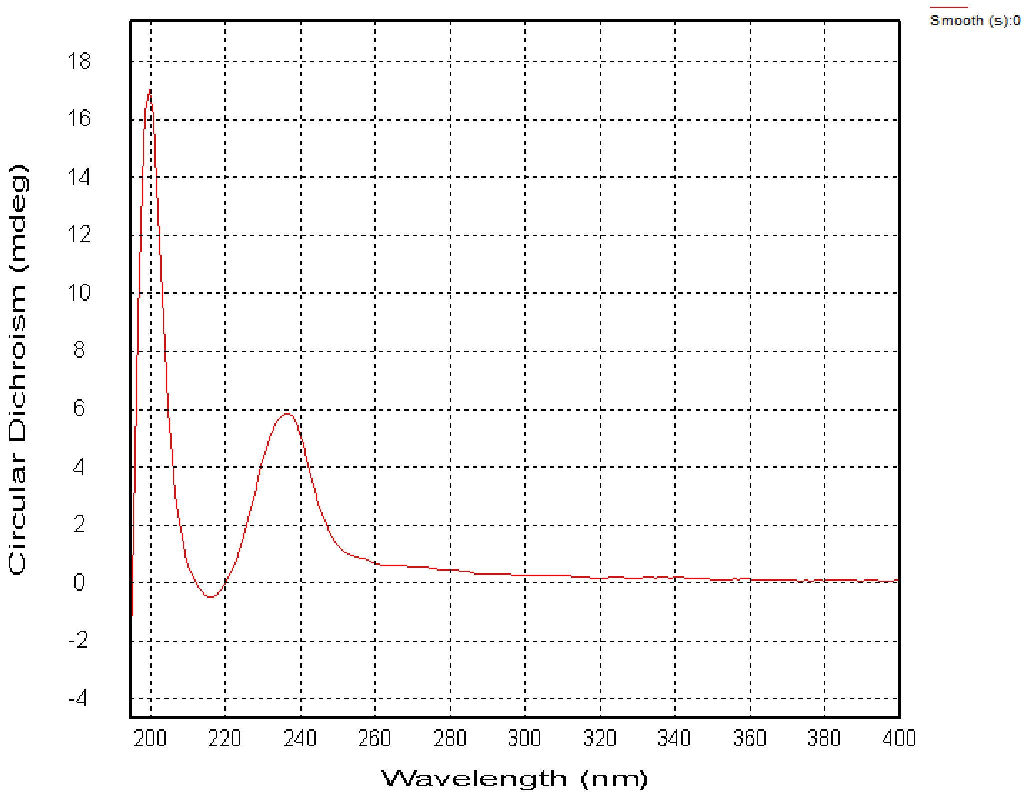

Figure S10. CD spectrum of 1.

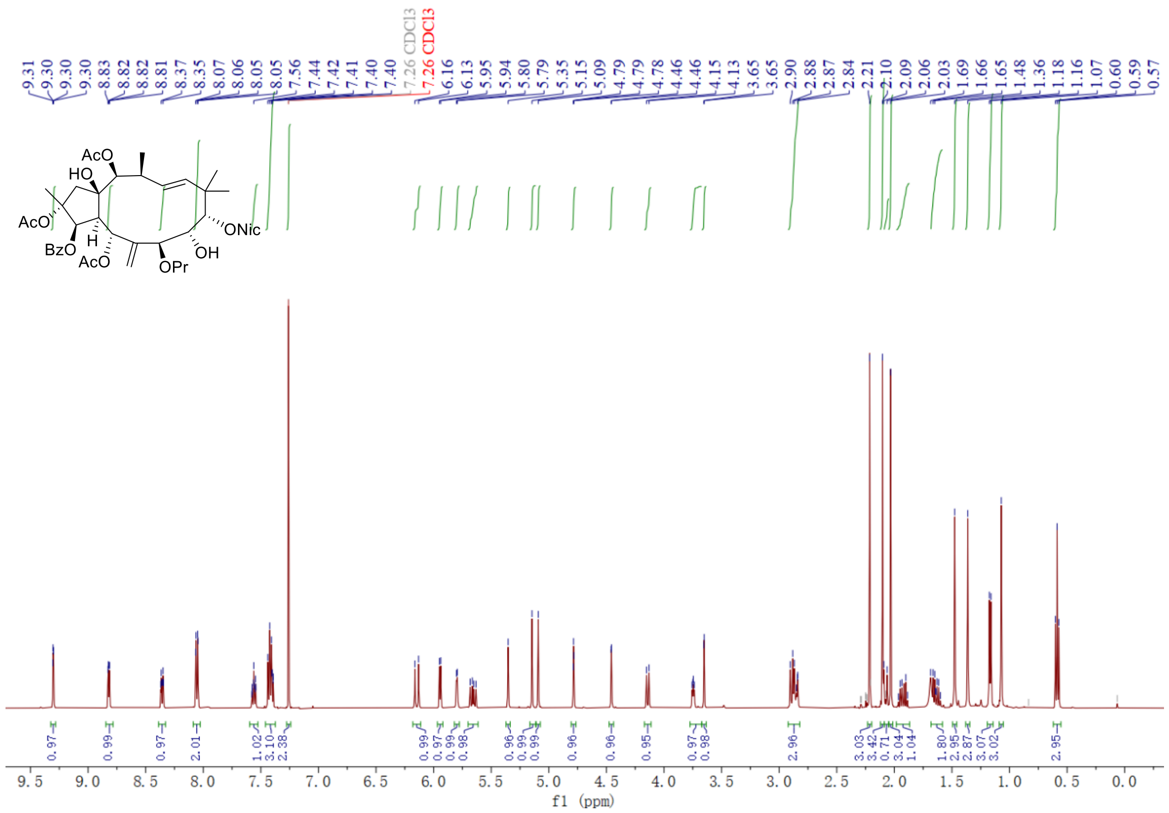

Figure S11. ^{1}H NMR (500 MHz) spectrum of 2 in $CDCl_3$.

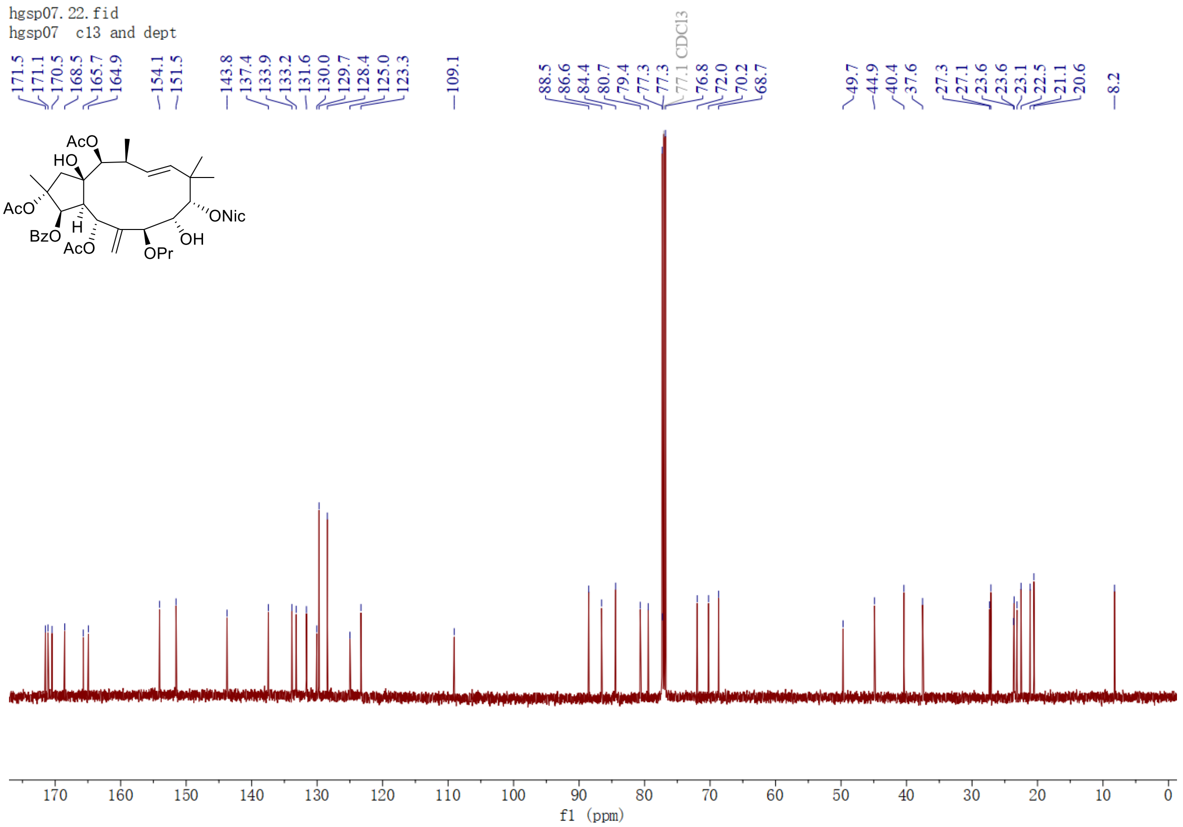

Figure S12. ^{13}C NMR (125 MHz) spectrum of 2 in $CDCl_3$.

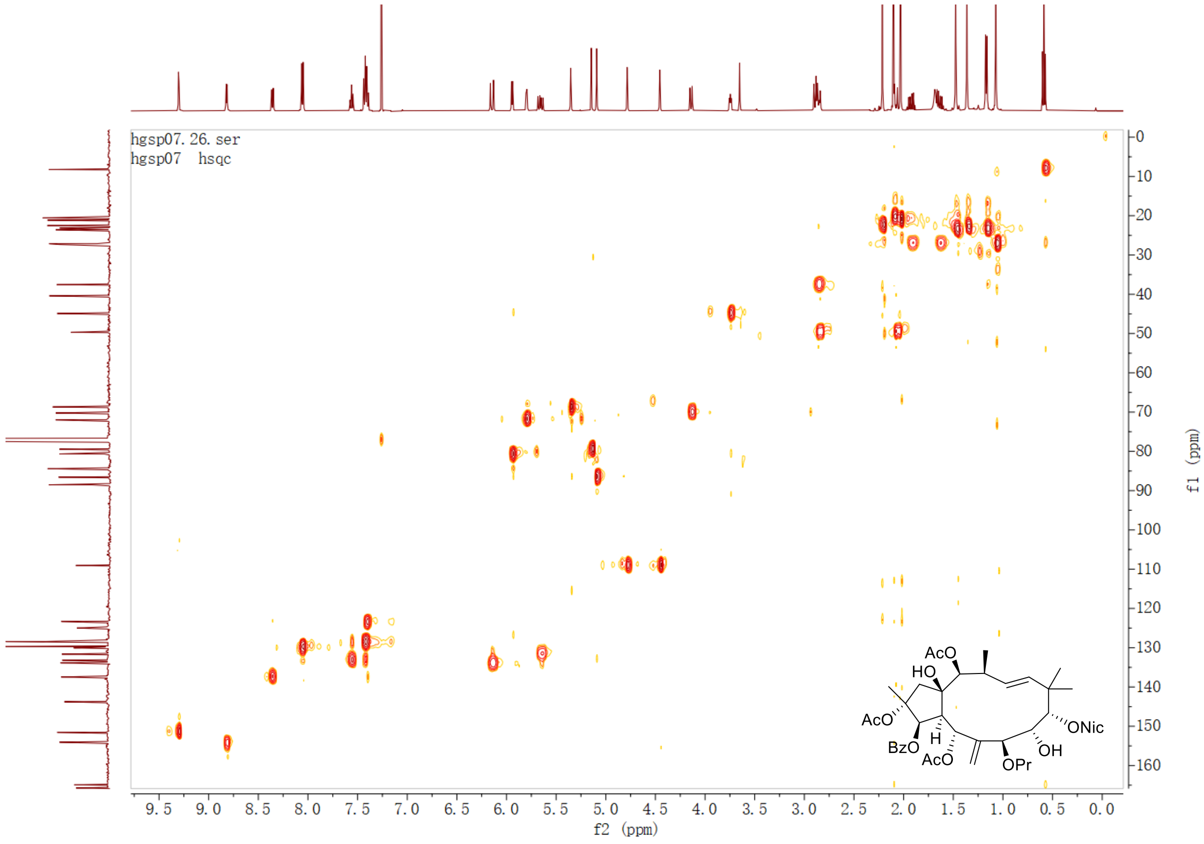

Figure S13. HSQC (500 MHz) spectrum of 2 in $CDCl_3$.

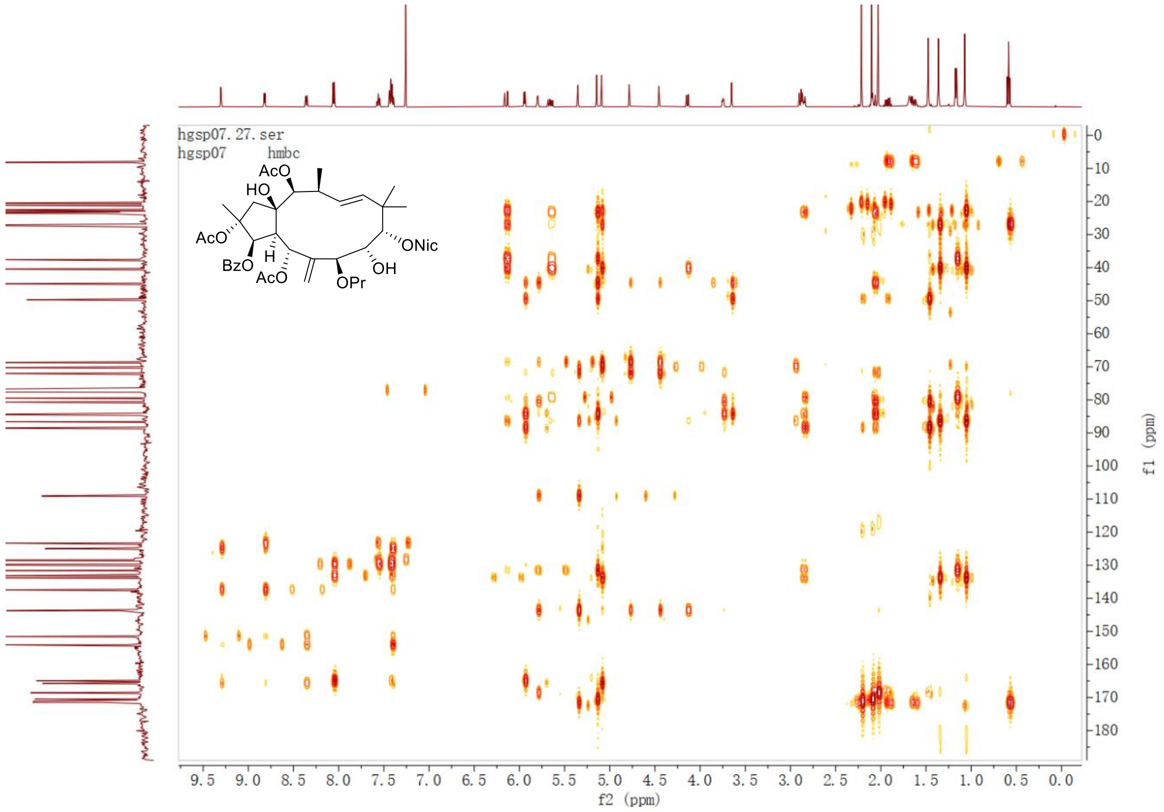

Figure S14. HMBC (500 MHz) spectrum of 2 in $CDCl_3$.

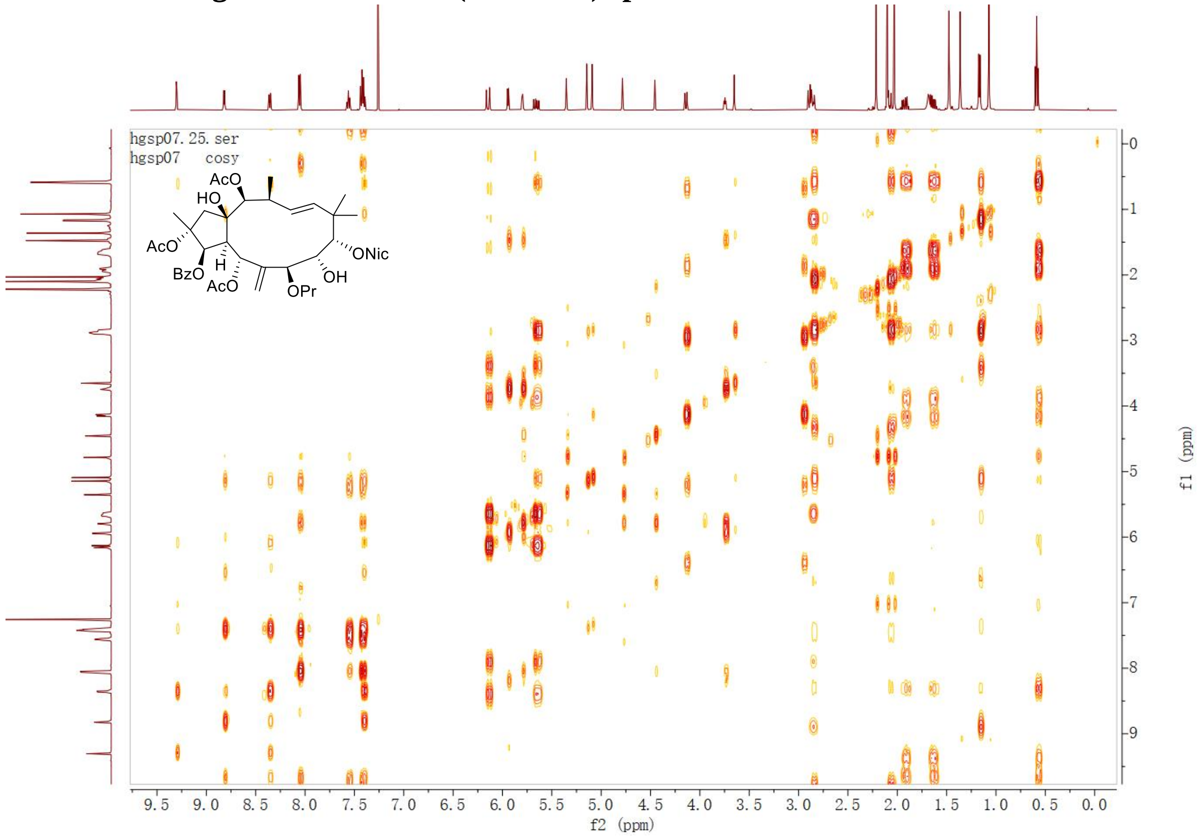

Figure S15. ^1H-^1H COSY (500 MHz) spectrum of 2 in $CDCl_3$.

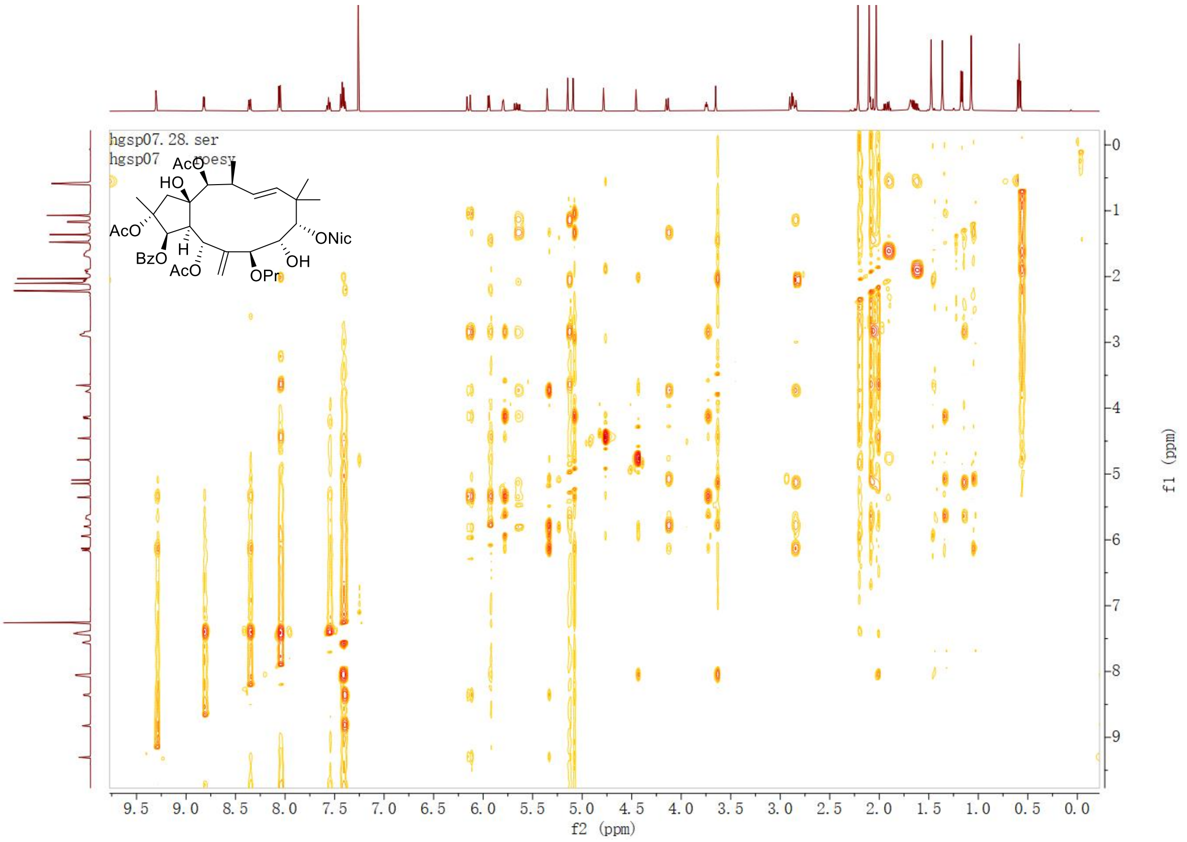

Figure S16. ROESY (500 MHz) spectrum of 2 in $CDCl_3$.

Qualitative Analysis Report

**Data Filename**	h-gsp-07.d	**Sample Name**	h-gsp-07
**Sample Type**	Sample	**Position**	P1-A1
**Instrument Name**	Instrument 1	**User Name**	
**Acq Method**	s.m	**Acquired Time**	2/10/2023 11:16:10 AM
**IRM Calibration Status**	Success	**DA Method**	PCDL.m
**Comment**			

**Sample Group**		**Info.**
**Acquisition SW Version**	6200 series TOF/6500 series Q-TOF B.05.01 (B5125.2)	

User Spectra

Fragmentor Voltage	Collision Energy	Ionization Mode
135	0	ESI

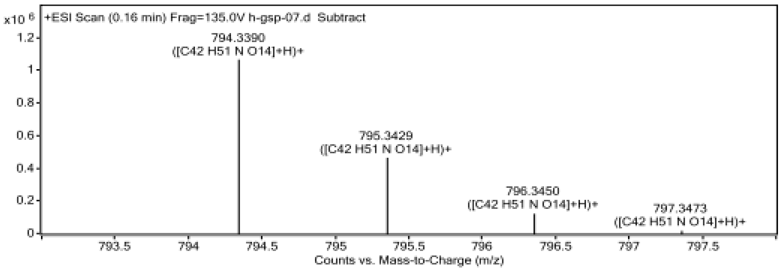

Peak List

m/z	z	Abund	Formula	Ion
706.3441	1	51277.5		
794.339	1	1069011.38	C42 H51 N O14	(M+H)+
795.3429	1	468487.13	C42 H51 N O14	(M+H)+
796.345	1	129950.75	C42 H51 N O14	(M+H)+
816.3211	1	274166.81		
817.3241	1	123371.66		
1609.6541	1	372255.69		
1610.6579	1	335201.84		
1611.6599	1	171037.44		
1612.6626	1	54834.98		

Formula Calculator Element Limits

Element	Min	Max
C	3	60
H	0	150
O	0	20
N	0	3

Formula Calculator Results

Formula	CalculatedMass	CalculatedMz	Mz	Diff. (mDa)	Diff. (ppm)	DBE
C42 H51 N O14	793.3310	794.3382	794.3390	-0.80	-1.01	18.0000

--- End Of Report ---

Figure S17. HRESIMS spectrums of 2.

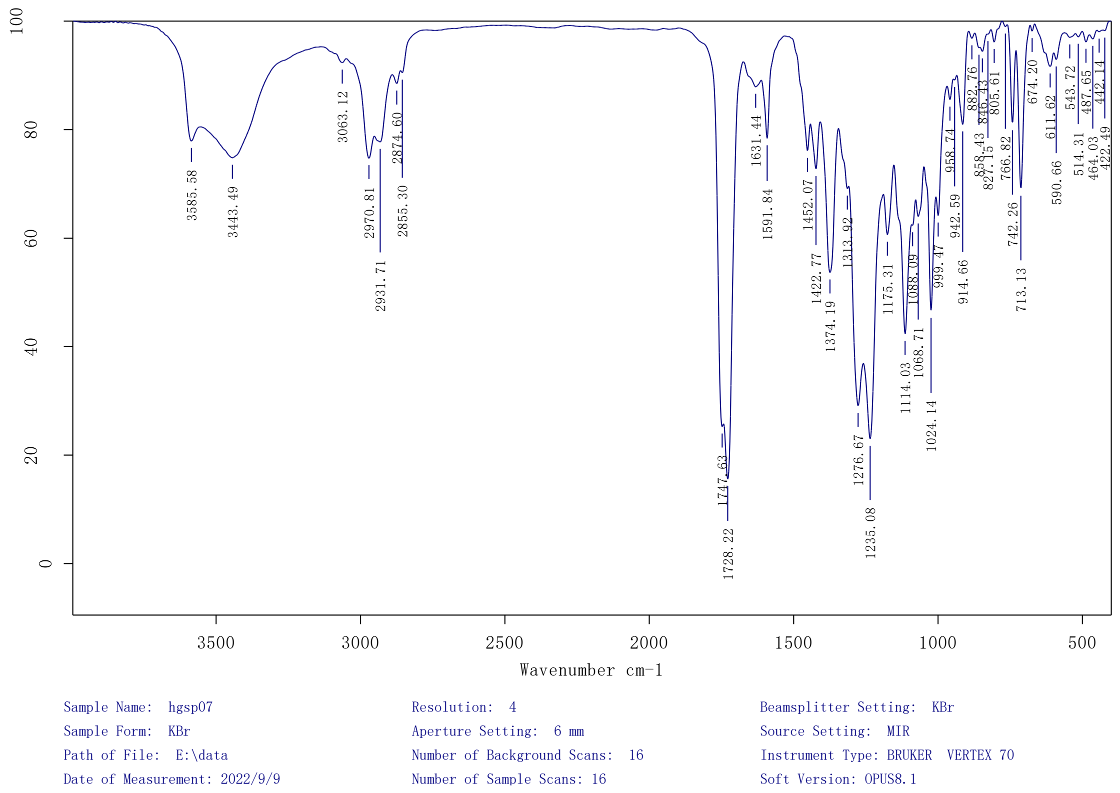

Figure S18. IR spectrum of 2.

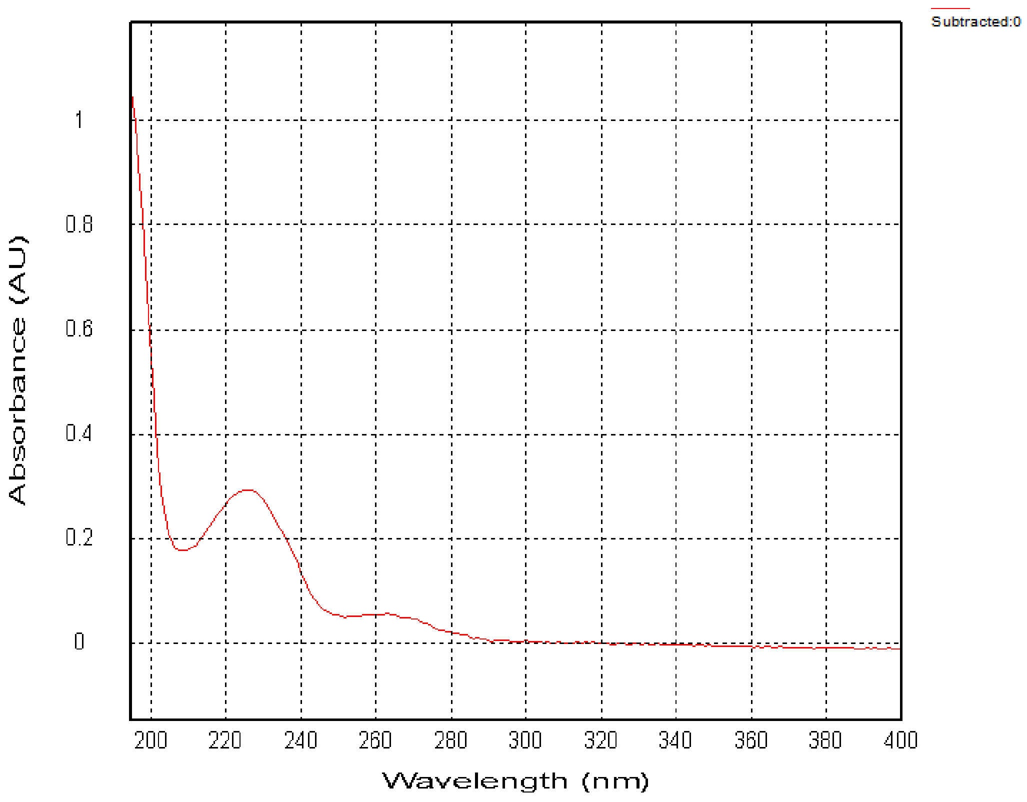

Figure S19. UV spectrum of 2.

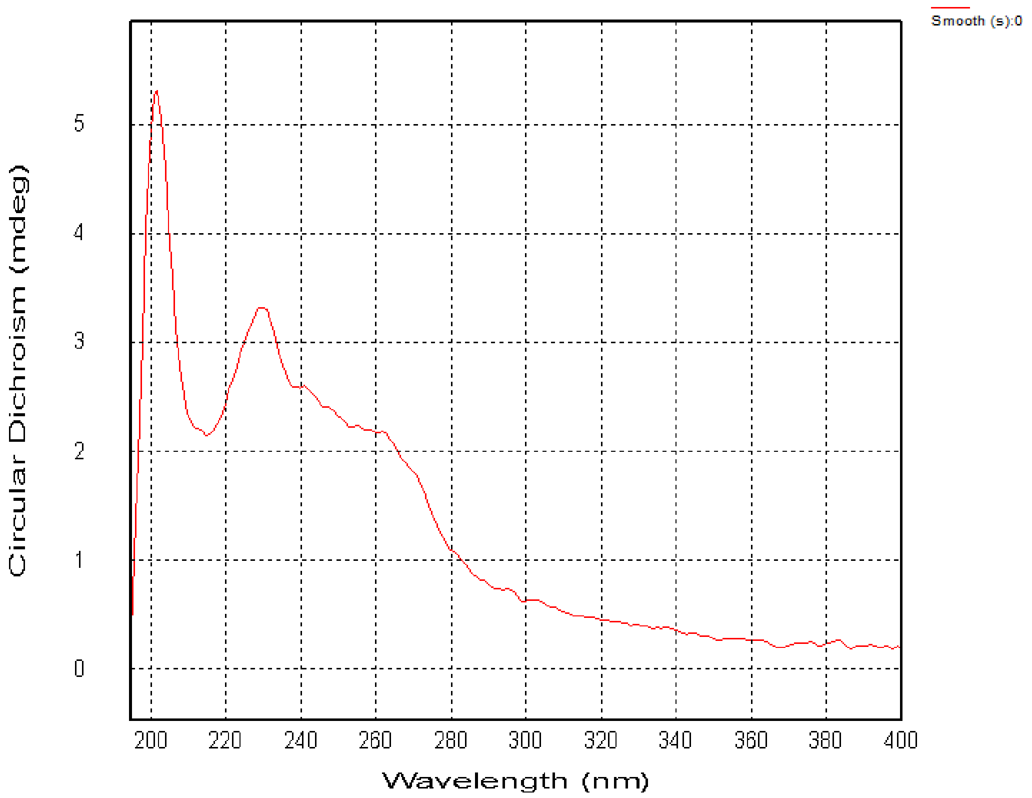

Figure S20. CD spectrum of 2.

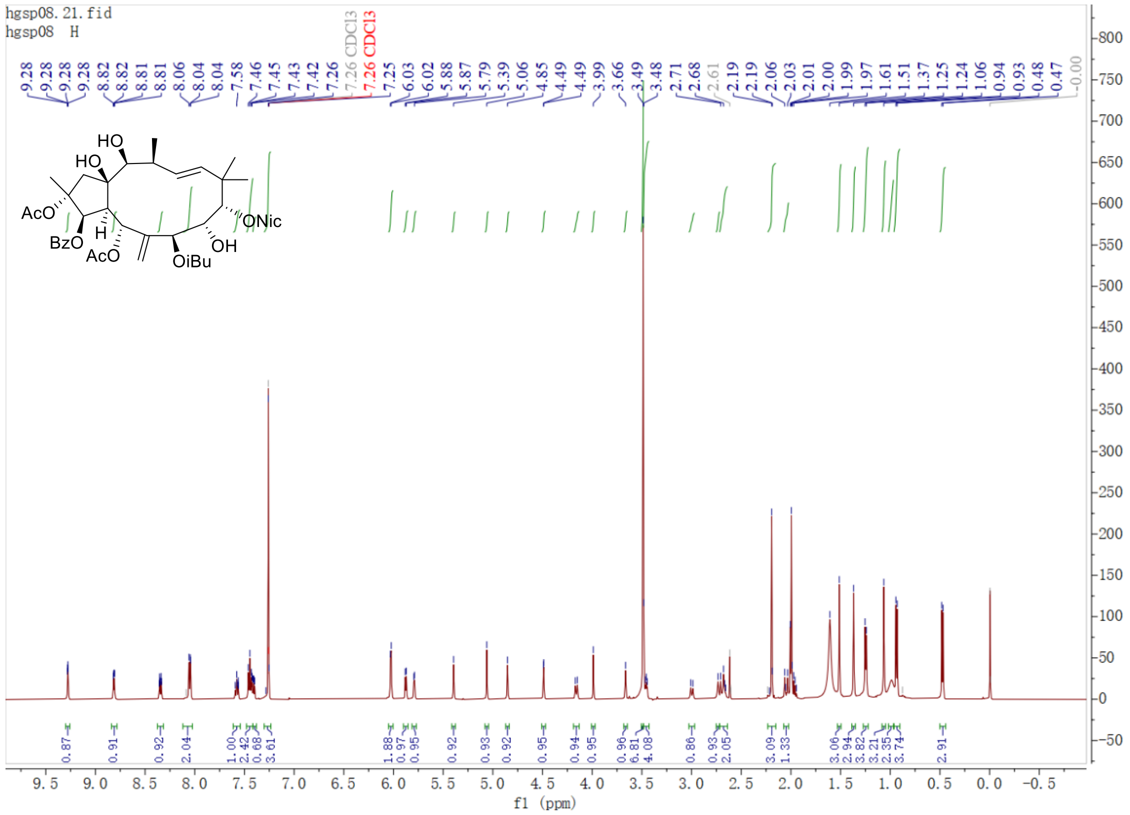

Figure S21. ^{1}H NMR (500 MHz) spectrum of 3 in $CDCl_3$.

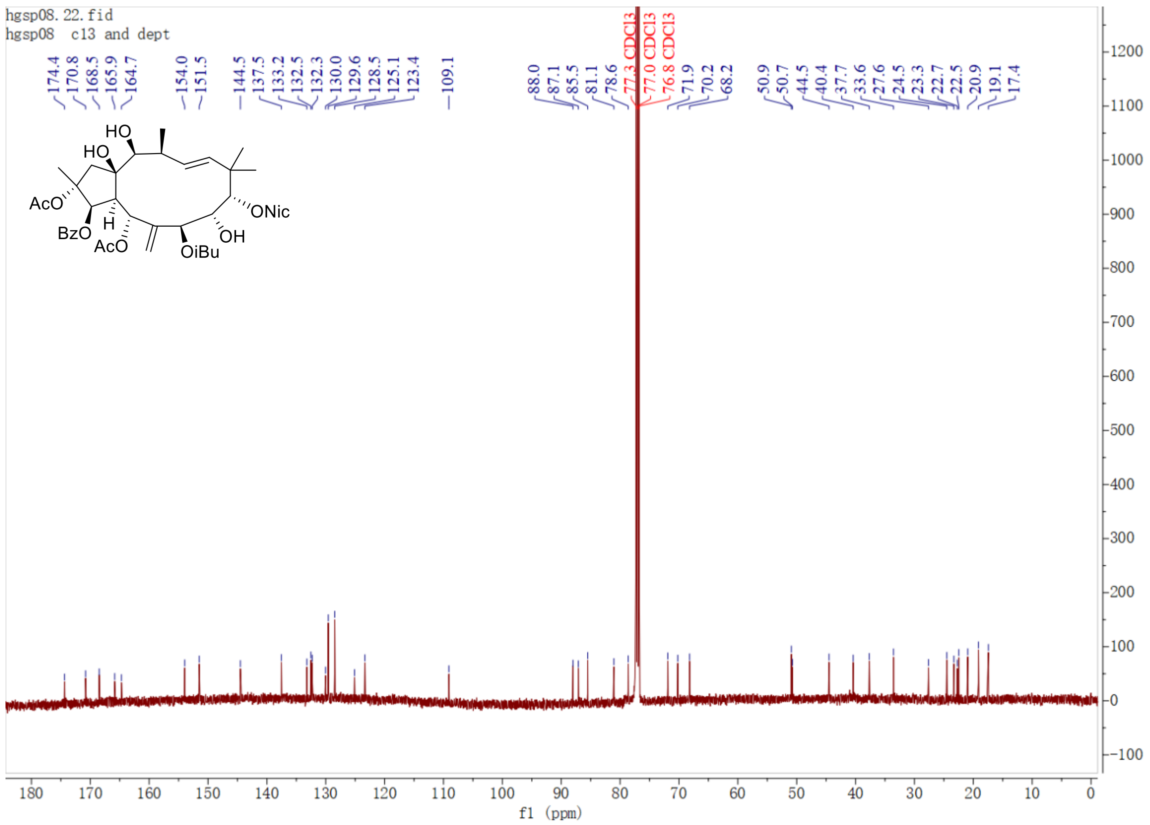

Figure S22. ^{13}**C NMR (125 MHz) spectrum of 3 in CDCl**$_3$**.**

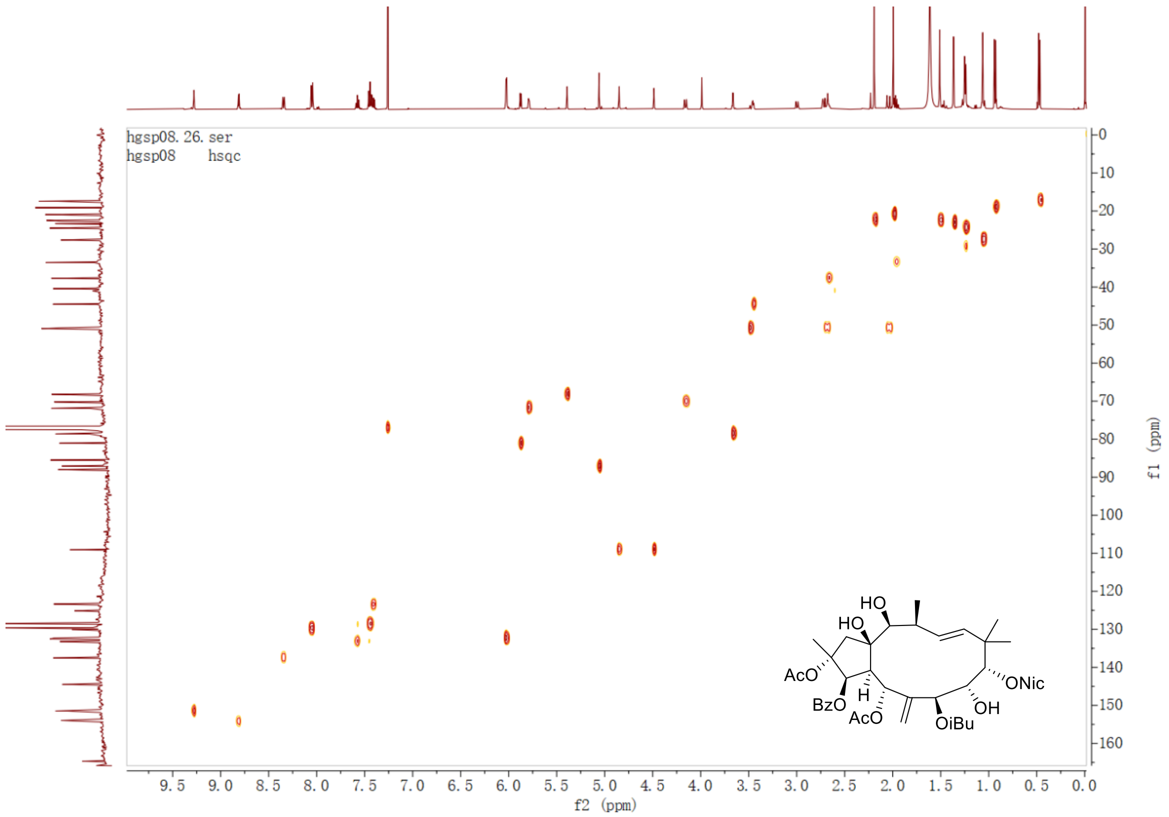

Figure S23. HSQC (500 MHz) spectrum of 3 in CDCl$_3$**.**

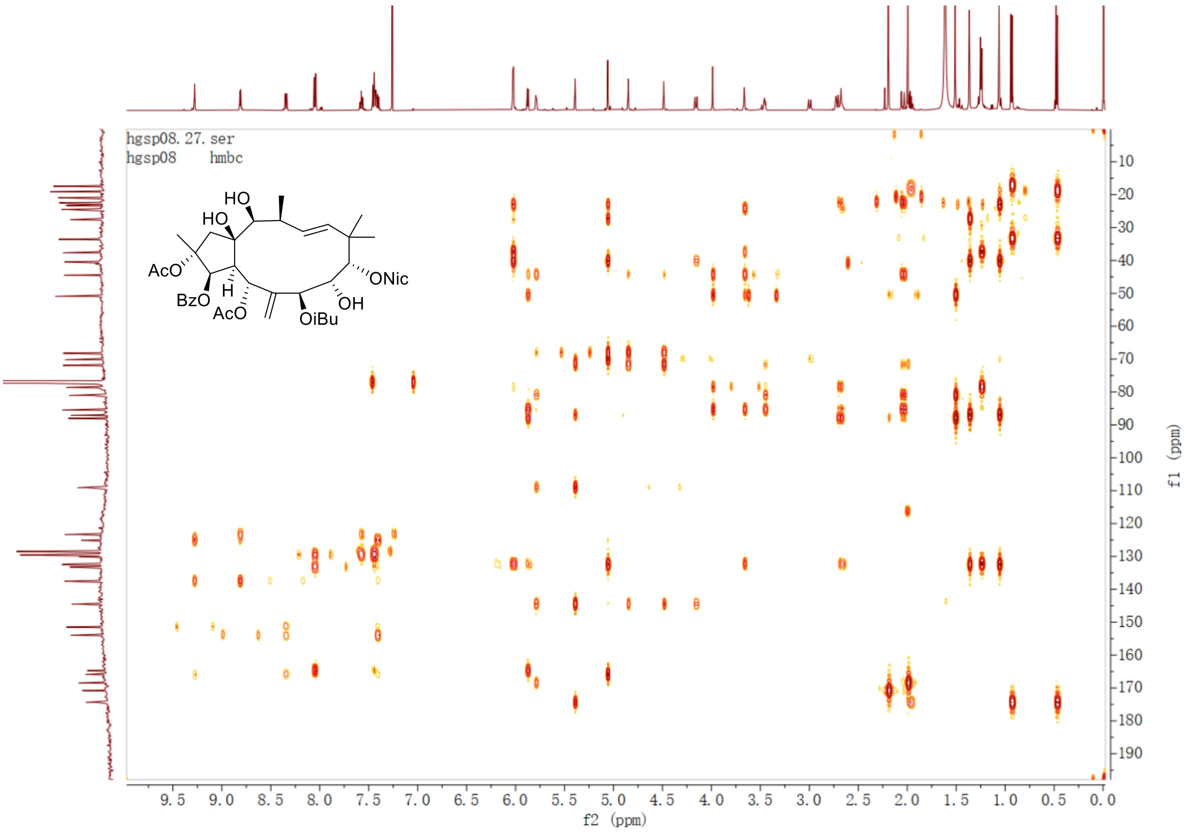

Figure S24. HMBC (500 MHz) spectrum of 3 in $CDCl_3$.

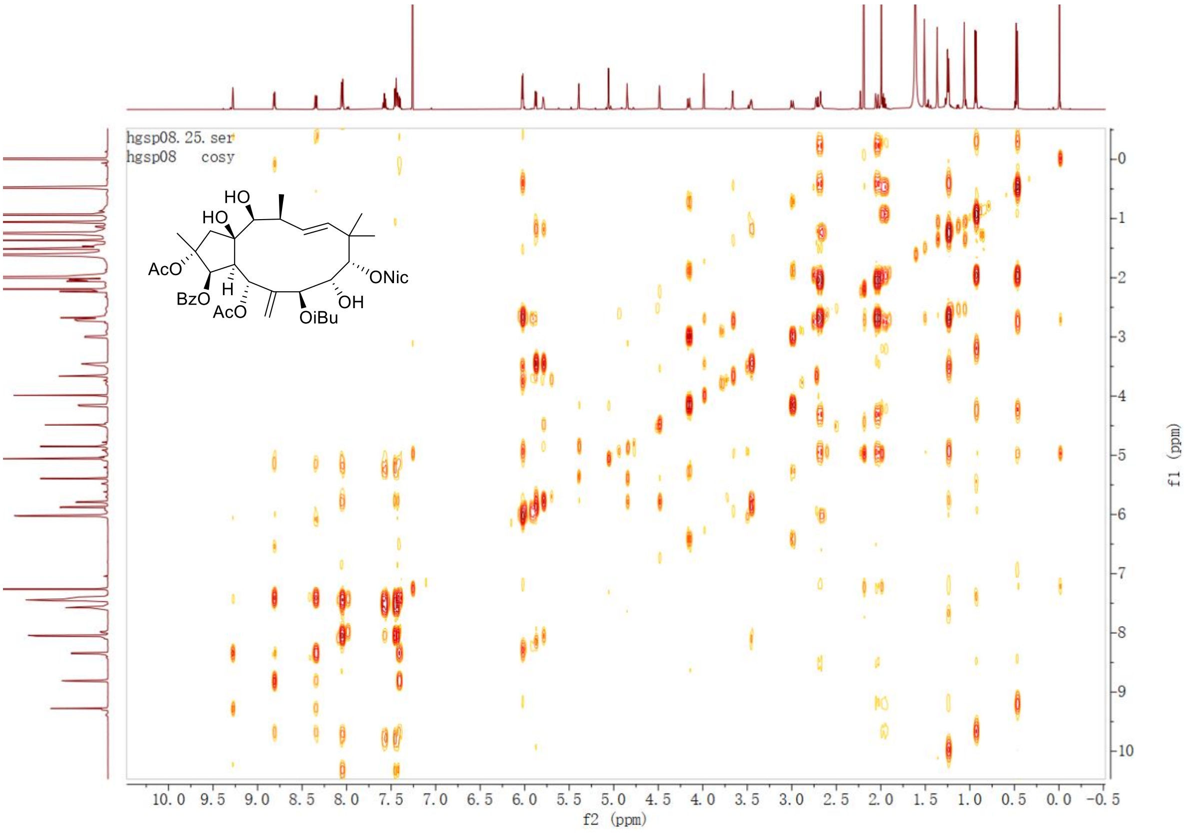

Figure S25. ^{1}H-^{1}H COSY (500 MHz) spectrum of 3 in $CDCl_3$.

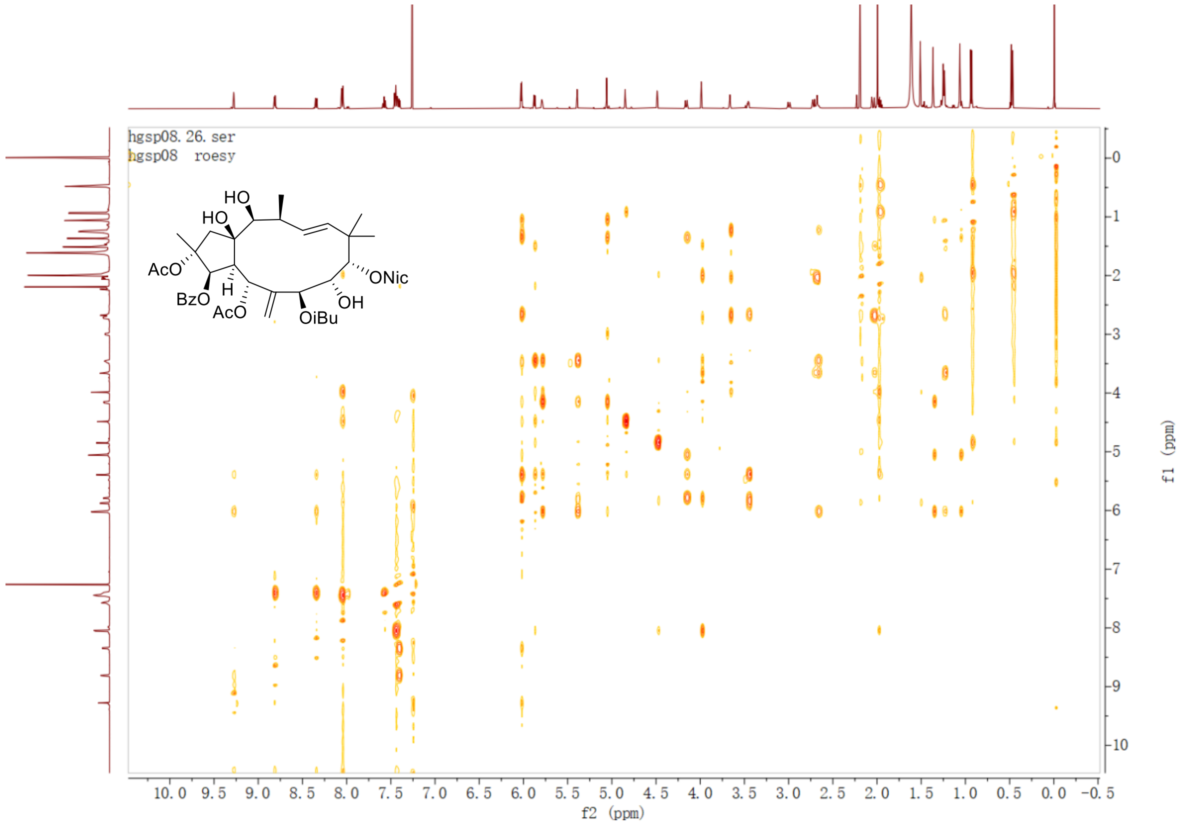

Figure S26. ROESY (500 MHz) spectrum of 3 in $CDCl_3$.

Qualitative Analysis Report

**Data Filename**	h-gsp-08.d	**Sample Name**	h-gsp-08
**Sample Type**	Sample	**Position**	P1-A2
**Instrument Name**	Instrument 1	**User Name**	
**Acq Method**	s.m	**Acquired Time**	2/10/2023 11:17:18 AM
**IRM Calibration Status**	Success	**DA Method**	PCDL.m
**Comment**			

**Sample Group**		**Info.**
**Acquisition SW Version**	6200 series TOF/6500 series Q-TOF B.05.01 (B5125.2)	

User Spectra

Fragmentor Voltage	Collision Energy	Ionization Mode
135	0	ESI

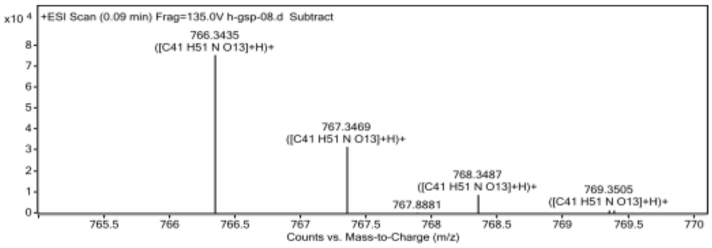

Peak List

m/z	z	Abund	Formula	Ion
724.3331	1	55415.91		
725.3355	1	23768.07		
726.3387	1	6816.42		
746.3147	1	21732.59		
747.3175	1	9219.55		
766.3435	1	75496.04	C41 H51 N O13	(M+H)+
767.3469	1	31761.44	C41 H51 N O13	(M+H)+
768.3487	1	8990.19	C41 H51 N O13	(M+H)+
788.3249	1	51330.78		
789.328	1	22290.99		

Formula Calculator Element Limits

Element	Min	Max
C	3	60
H	0	150
O	0	20
N	0	3

Formula Calculator Results

Formula	CalculatedMass	CalculatedMz	Mz	Diff. (mDa)	Diff. (ppm)	DBE
C41 H51 N O13	765.3360	766.3433	766.3435	-0.20	-0.26	17.0000

--- End Of Report ---

Figure S27. HRESIMS spectrums of 3.

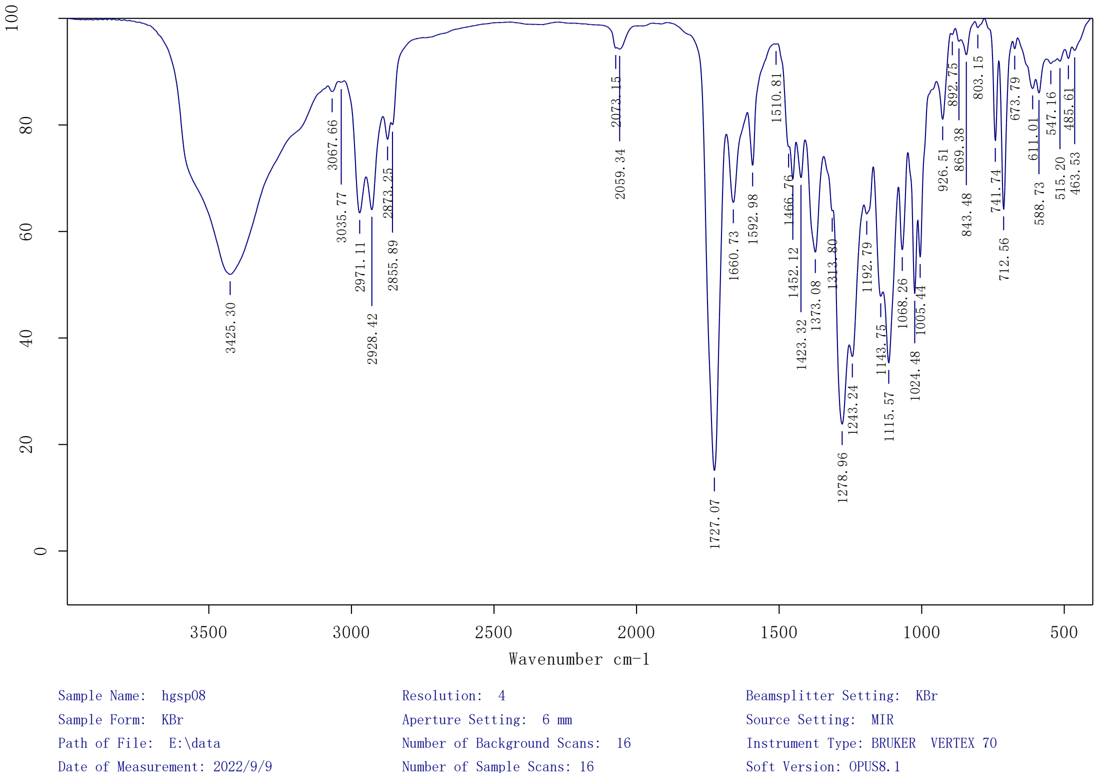

Figure S28. IR spectrum of 3.

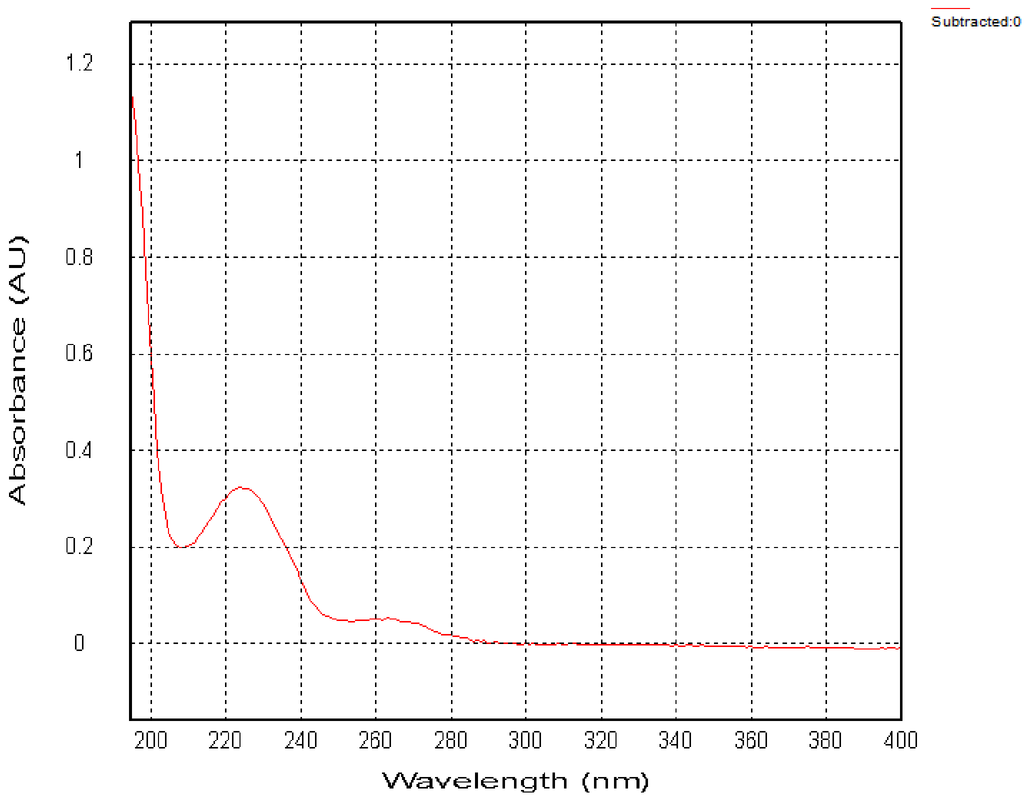

Figure S29. UV spectrum of 3.

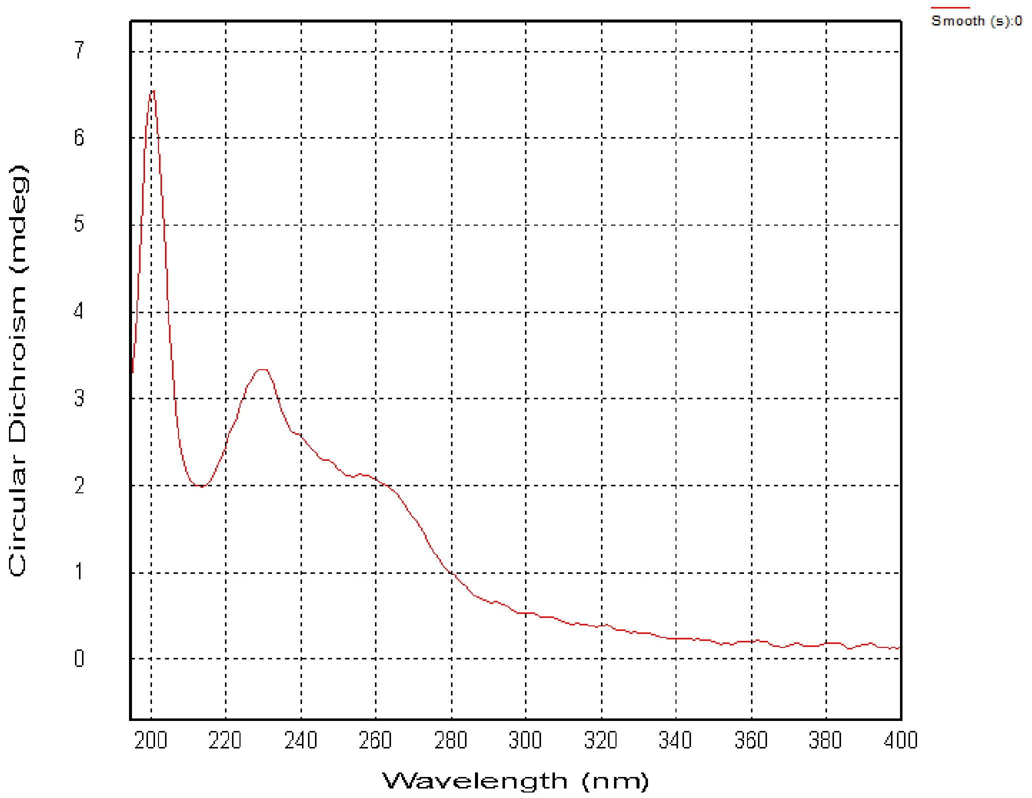

Figure S30. CD spectrum of 3.

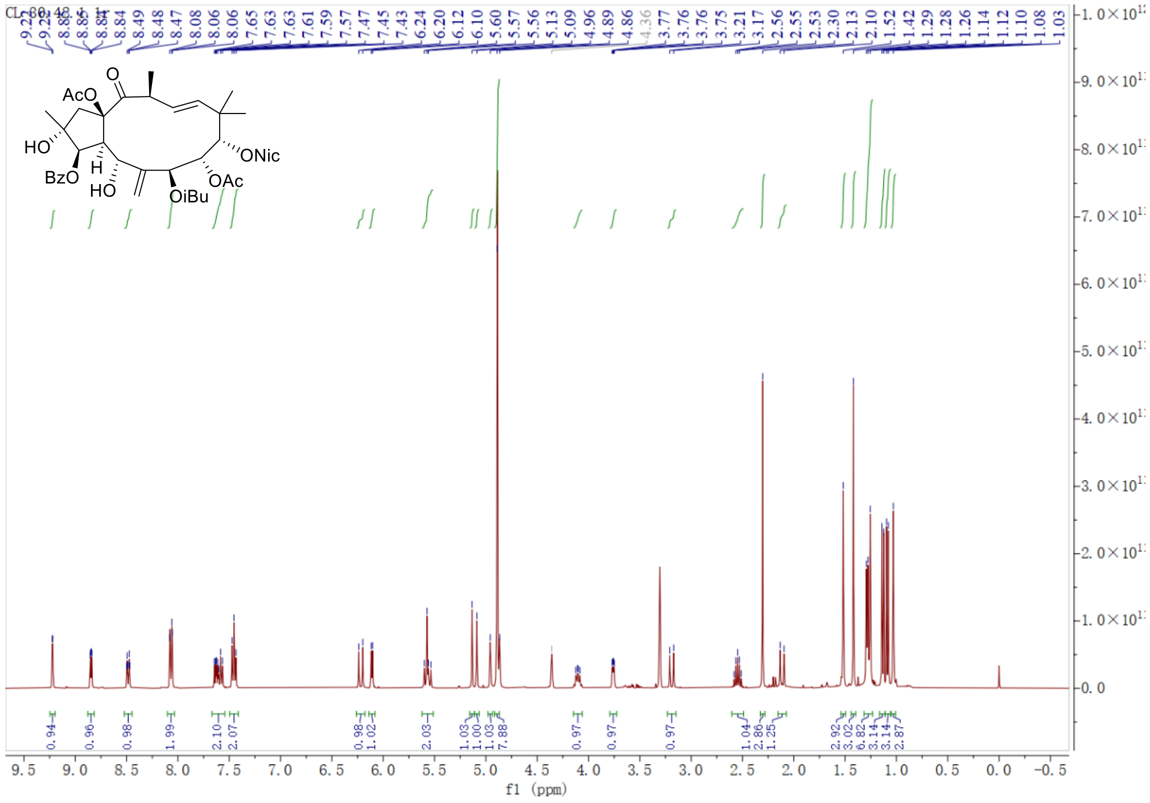

Figure S31. ^{1}H NMR (400 MHz) spectrum of 4 in CD_3OD.

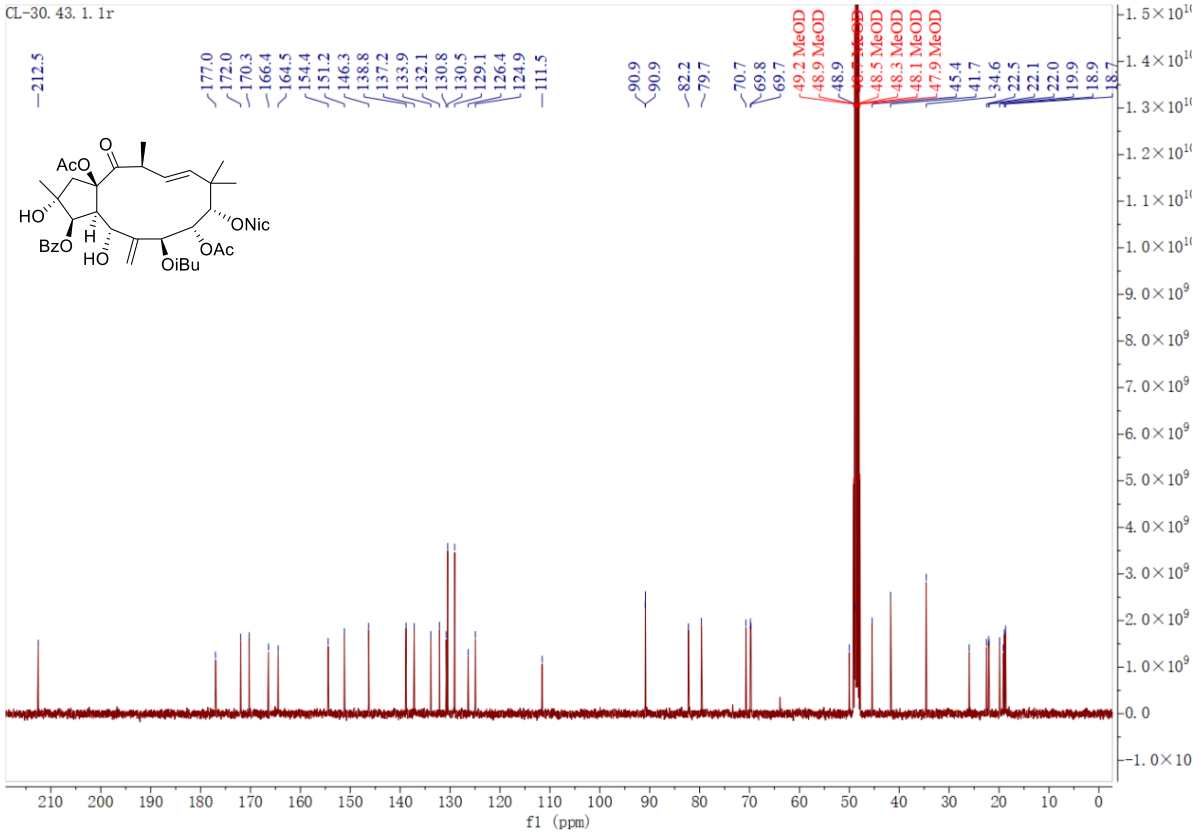

Figure S32. ^{13}**C NMR (100 MHz) spectrum of 4 in** CD_3OD**.**

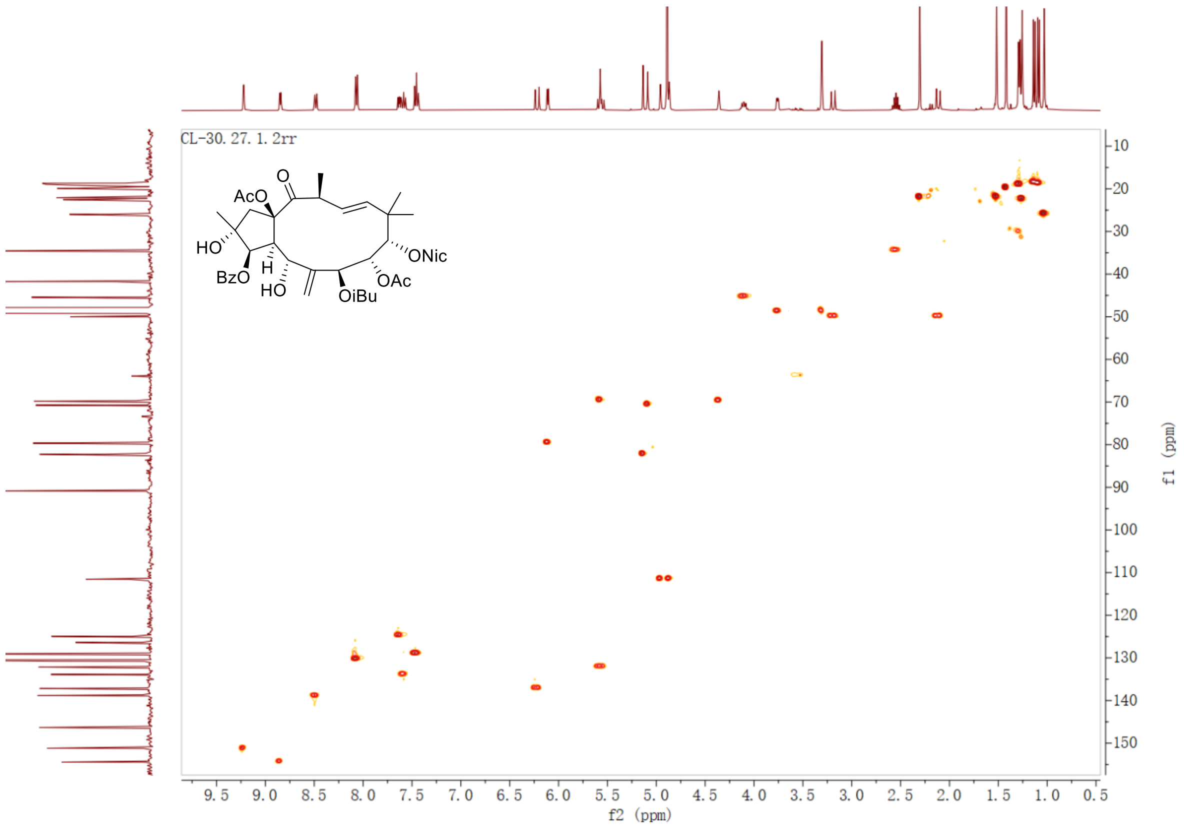

Figure S33. HSQC (400 MHz) spectrum of 4 in CD_3OD**.**

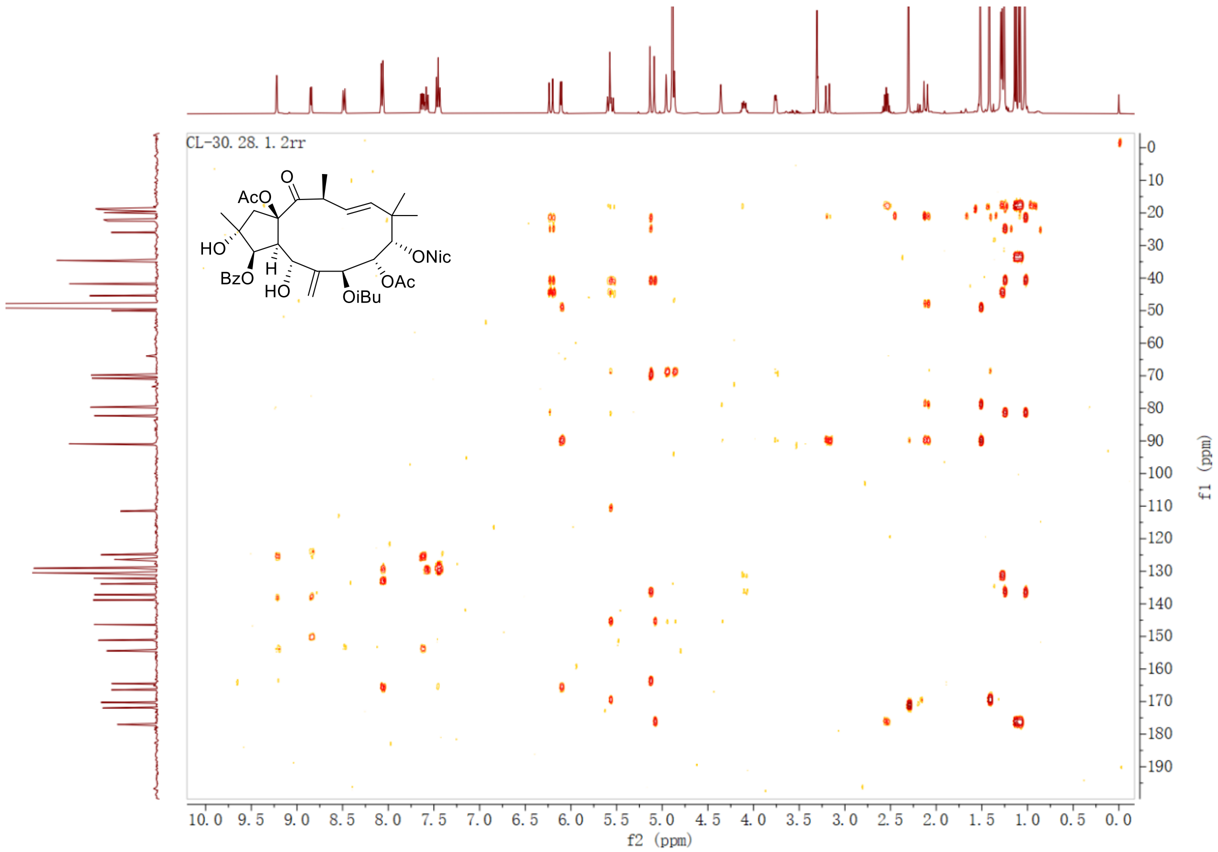

Figure S34. HMBC (400 MHz) spectrum of 4 in CD_3OD.

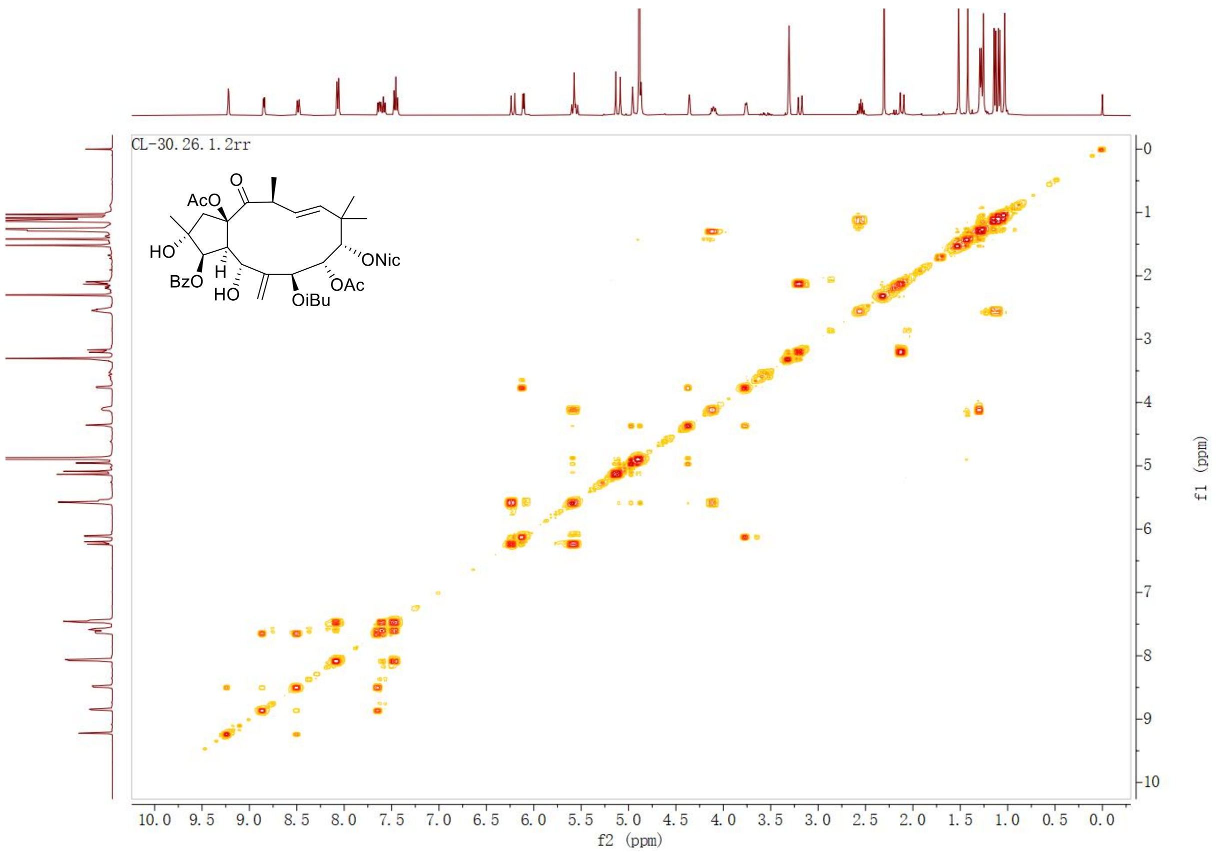

Figure S35. ^1H-^1H COSY (400 MHz) spectrum of 4 in CD_3OD.

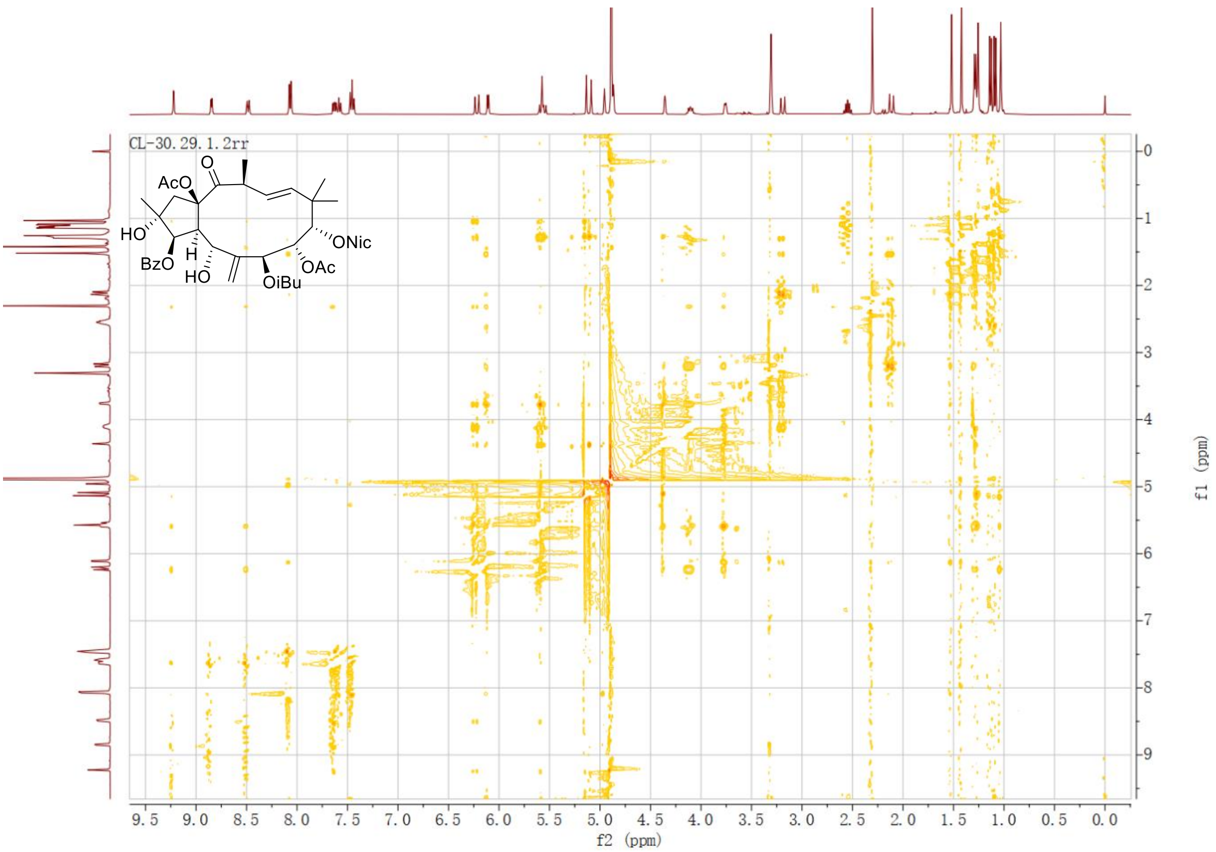

Figure S36. ROESY (400 MHz) spectrum of 4 in CD_3OD.

Elemental Composition Report **Page 1**

Single Mass Analysis
Tolerance = 100.0 PPM / DBE: min = -1.5, max = 100.0
Element prediction: Off
Number of isotope peaks used for i-FIT = 5

Monoisotopic Mass, Even Electron Ions
1 formula(e) evaluated with 1 results within limits (all results (up to 1000) for each mass)
Elements Used:
C: 41-41 H: 48-50 N: 1-1 O: 13-13 Na: 1-1
20241203--CL-30 21 (0.213)
1: TOF MS ES+

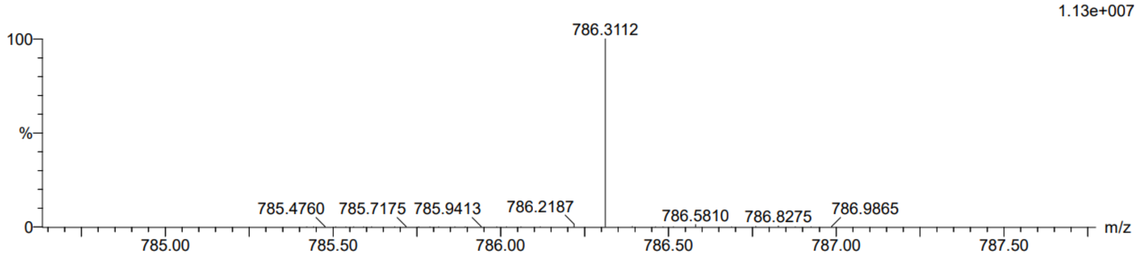

Minimum:				-1.5				
Maximum:		5.0	100.0	100.0				

Mass	Calc. Mass	mDa	PPM	DBE	i-FIT	Norm	Conf(%)	Formula
786.3112	786.3102	1.0	1.3	17.5	228.8	n/a	n/a	C41 H49 N O13 Na

Figure S37. HRESIMS spectrums of 4.

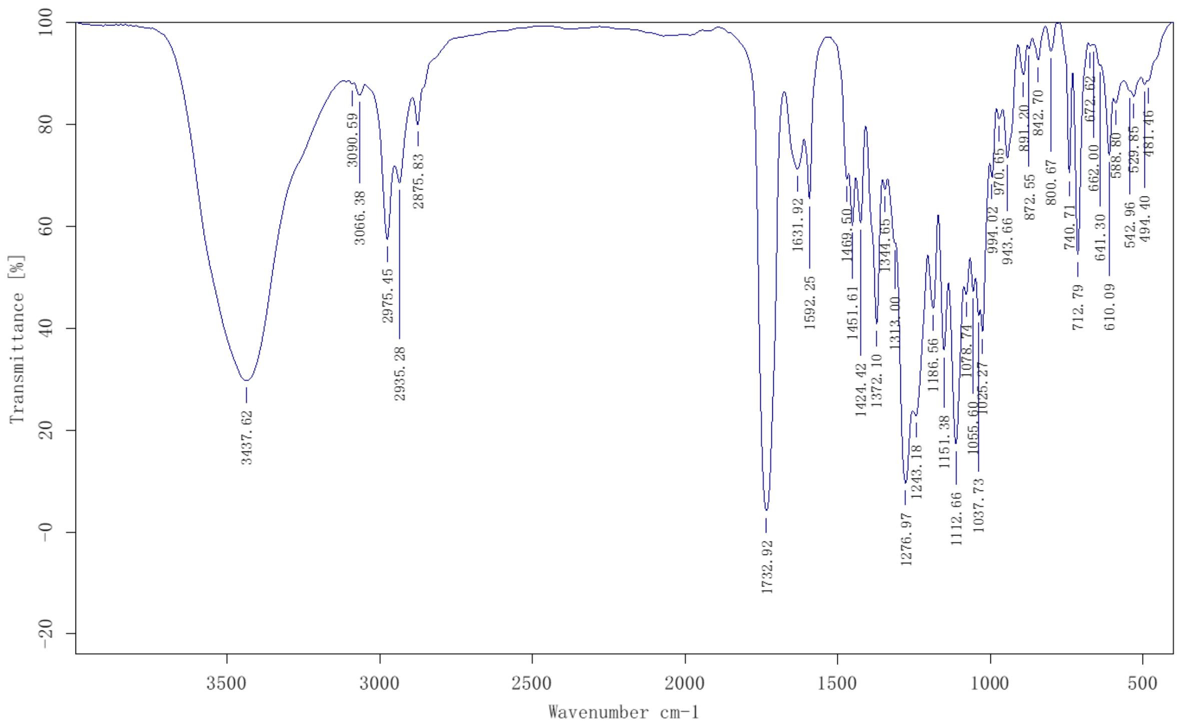

Figure S38. IR spectrum of 4.

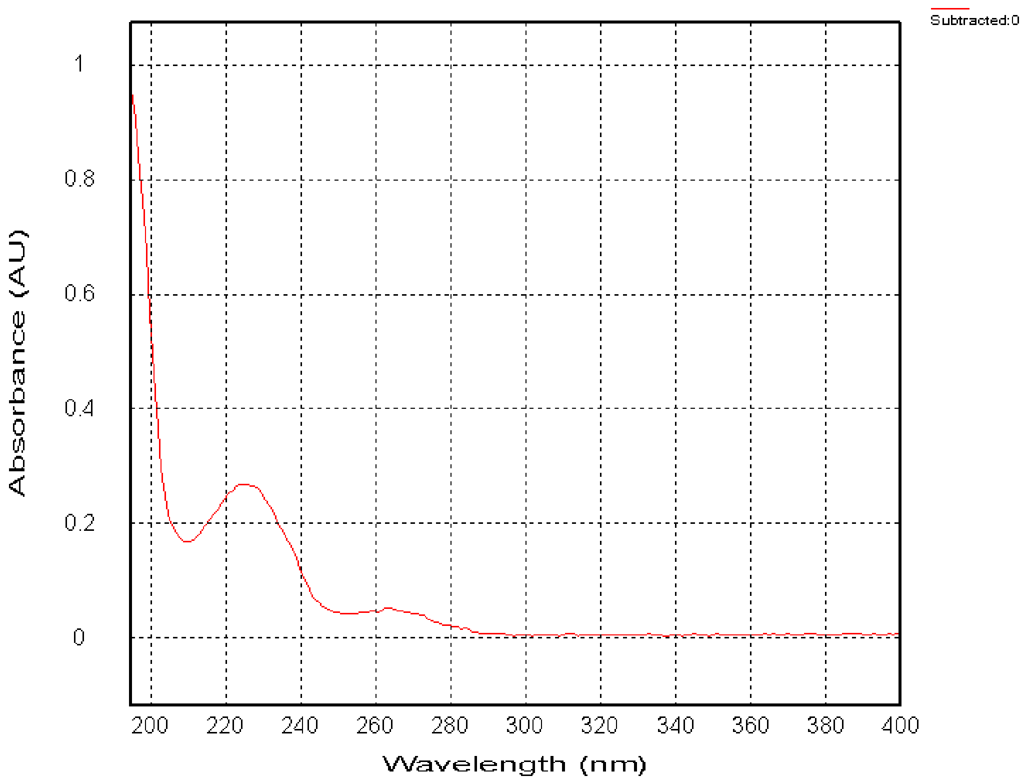

Figure S39. UV spectrum of 4.

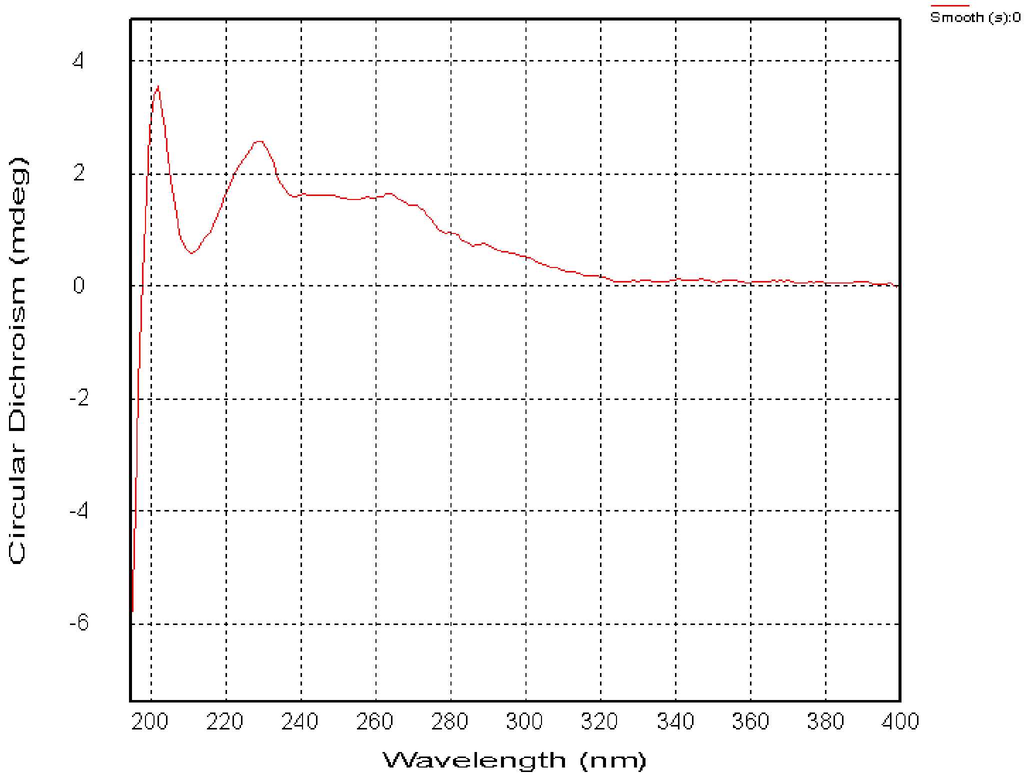

Figure S40. CD spectrum of 4.

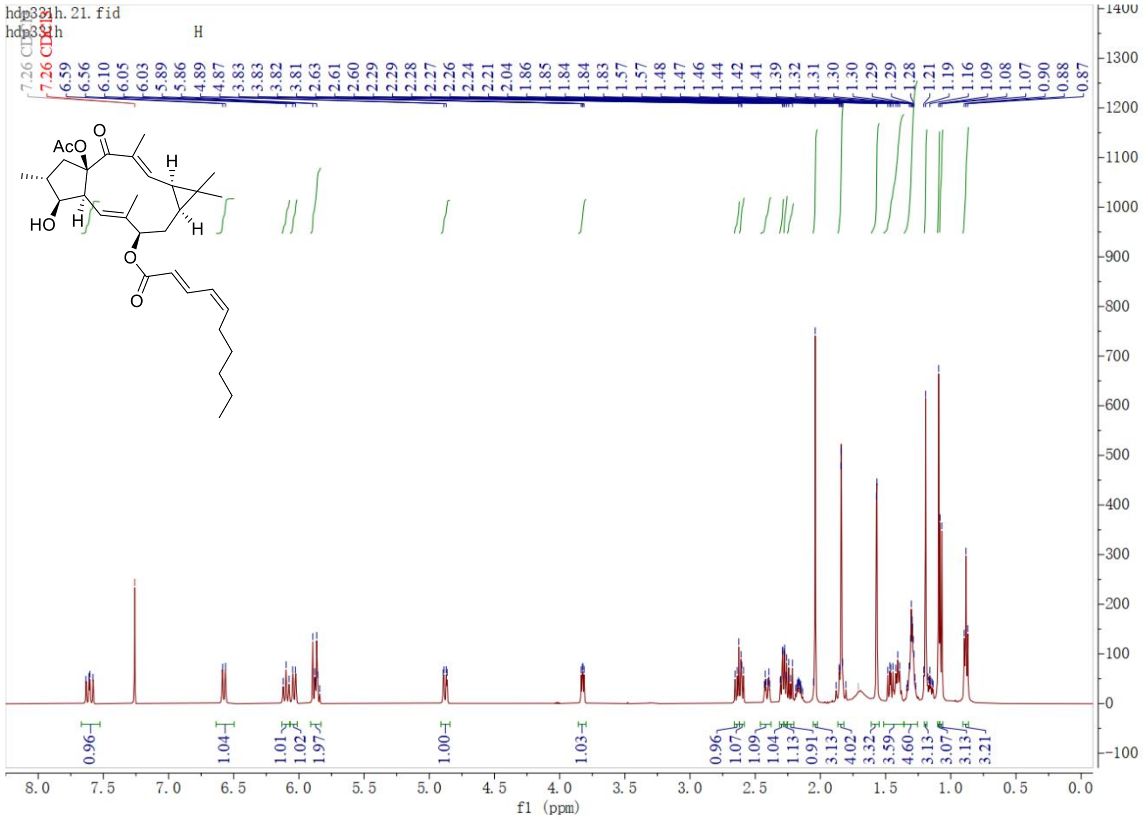

Figure S41. ^{1}H NMR (600 MHz) spectrum of 5 in $CDCl_3$.

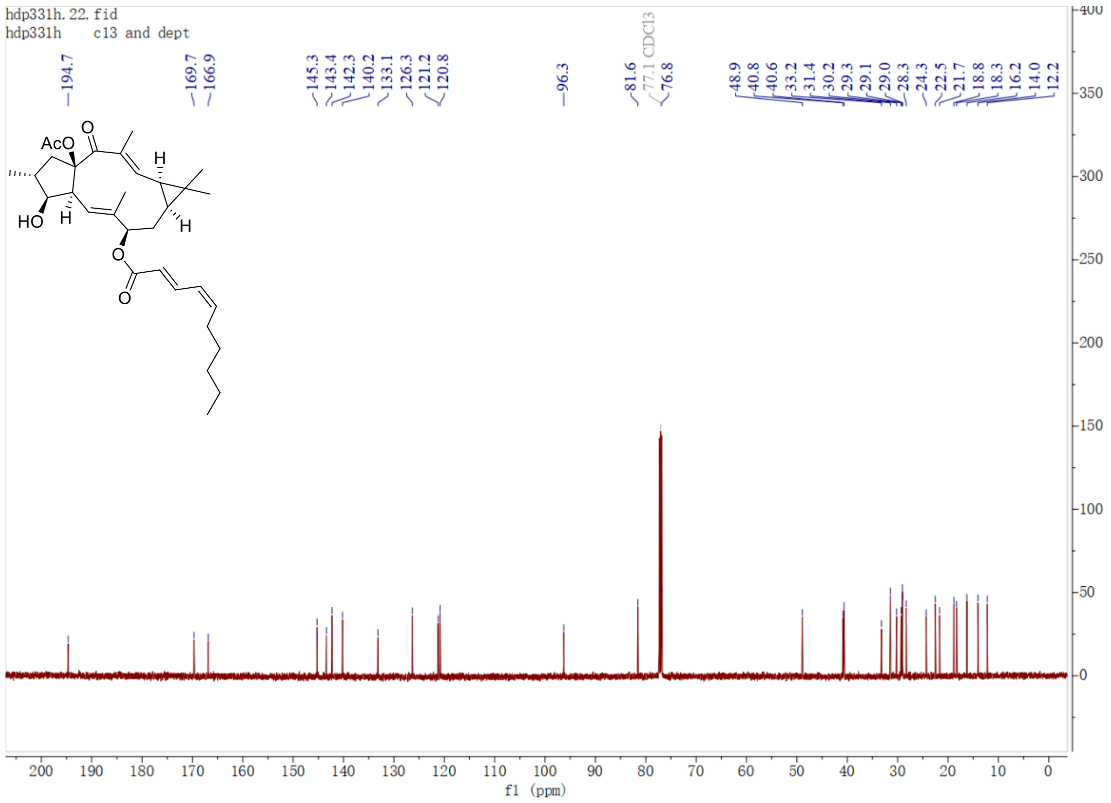

Figure S42. ^{13}C **NMR (150 MHz) spectrum of 5 in $CDCl_3$.**

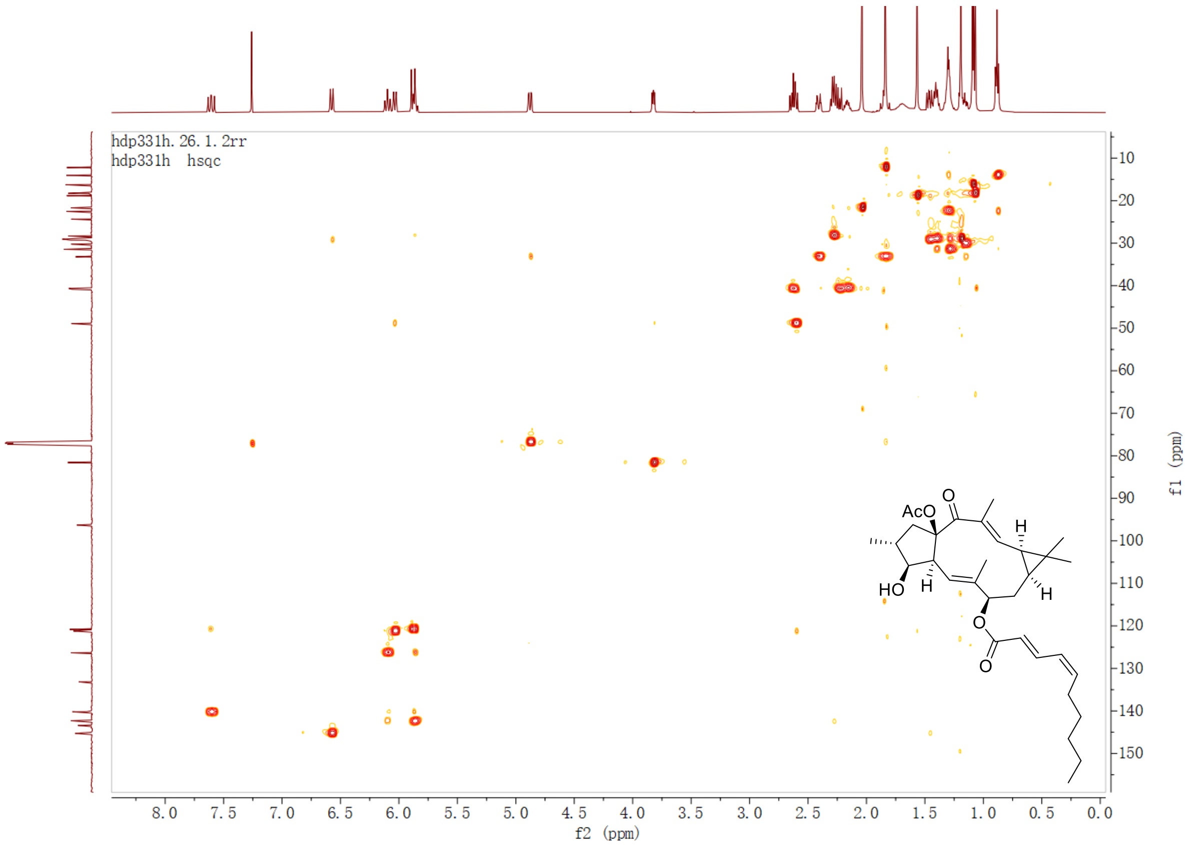

Figure S43. HSQC (600 MHz) spectrum of 5 in $CDCl_3$.

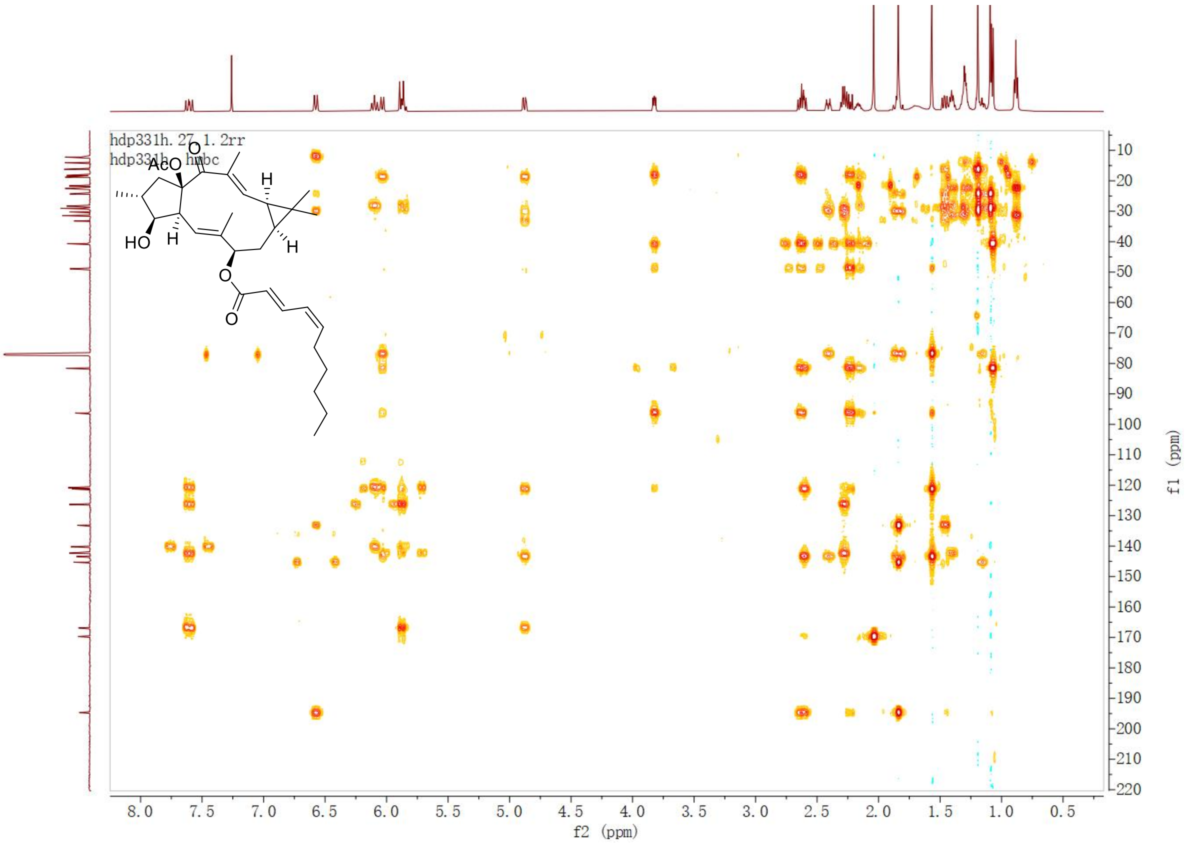

Figure S44. HMBC (600 MHz) spectrum of 5 in $CDCl_3$.

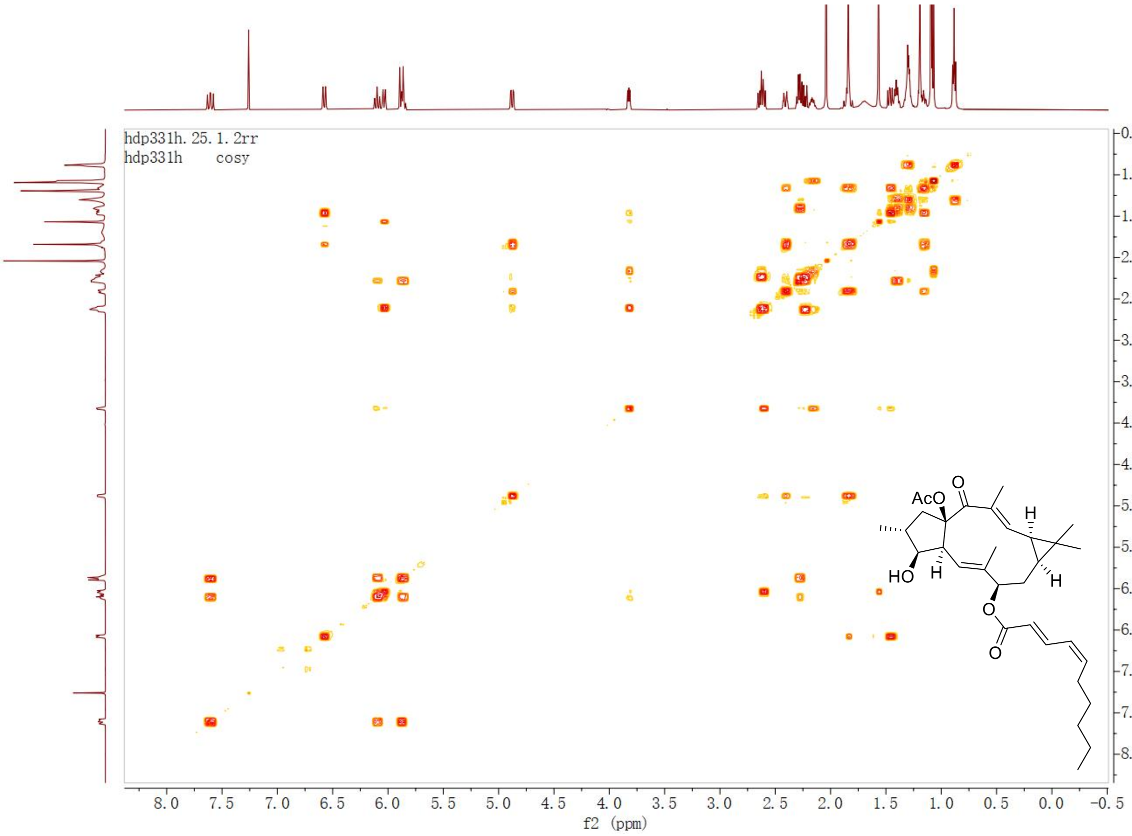

Figure S45. ^{1}H-^{1}H COSY (600 MHz) spectrum of 5 in $CDCl_3$.

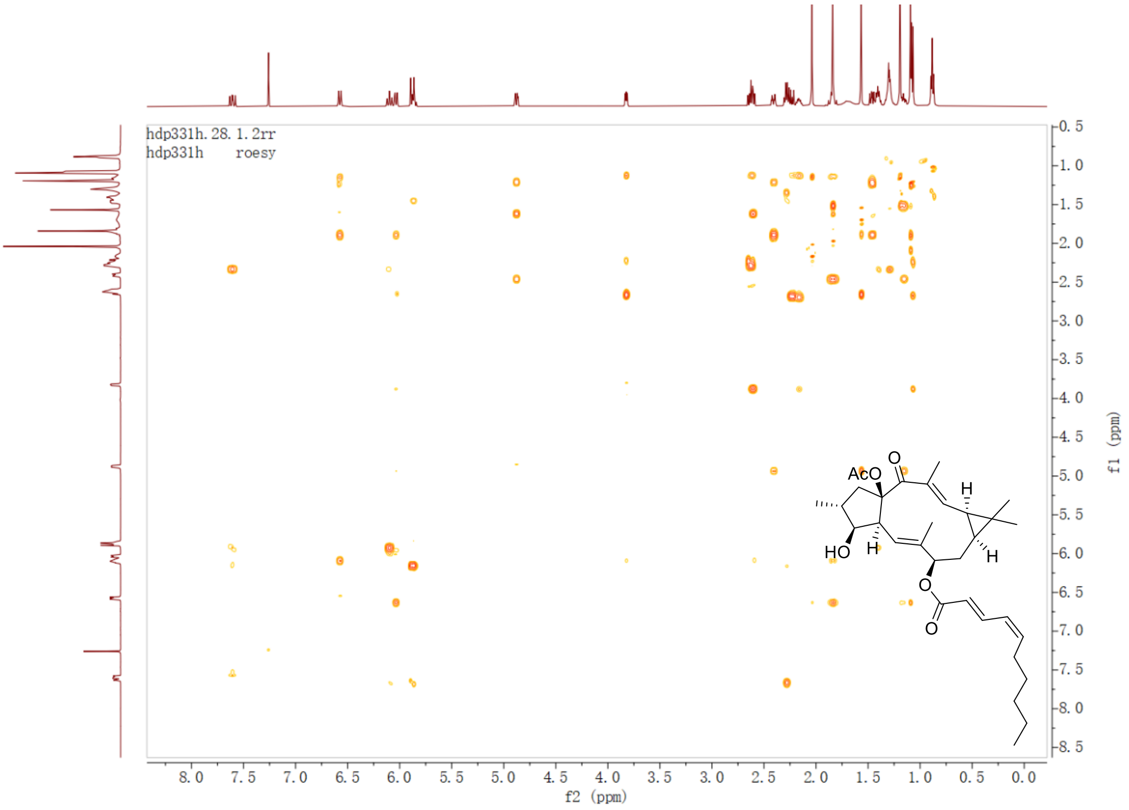

Figure S46. ROESY (600 MHz) spectrum of 5 in $CDCl_3$.

Qualitative Analysis Report

**Data Filename**	zq-12.d	**Sample Name**	zq-12
**Sample Type**	Sample	**Position**	P1-A1
**Instrument Name**	Instrument 1	**User Name**	
**Acq Method**	s.m	**Acquired Time**	11/29/2024 5:59:24 PM
**IRM Calibration Status**	Success	**DA Method**	PCDL.m
**Comment**			

**Sample Group**		**Info.**
**Acquisition SW**	6200 series TOF/6500 series	
**Version**	Q-TOF B.05.01 (B5125.2)	

User Spectra

Fragmentor Voltage	Collision Energy	Ionization Mode
135	0	ESI

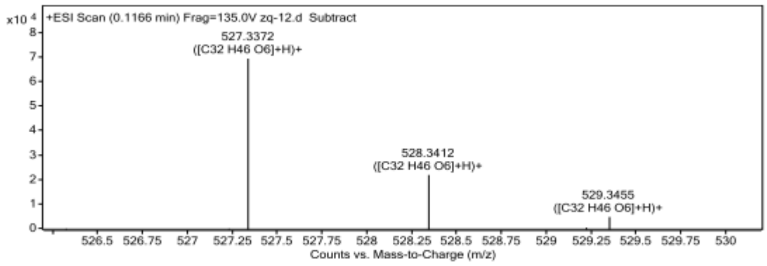

Peak List

m/z	z	Abund	Formula	Ion
299.2009	1	22550.65		
527.3372	1	69600.47	C32 H46 O6	(M+H)+
528.3412	1	22362.63	C32 H46 O6	(M+H)+
543.3329	1	5479.95		
549.3194	1	23834.82		
550.3222	1	7903.65		
565.2981	1	6642.27		
698.4245	1	5856.83		
1075.6488	1	8356.03		
1076.6513	1	5862.95		

Formula Calculator Element Limits

Element	Min	Max
C	3	50
H	0	150
O	0	20

Formula Calculator Results

Formula	CalculatedMass	CalculatedMz	Mz	Diff. (mDa)	Diff. (ppm)	DBE
C32 H46 O6	526.3294	527.3367	527.3372	-0.50	-0.95	10.0000

Figure S47. HRESIMS spectrums of 5.

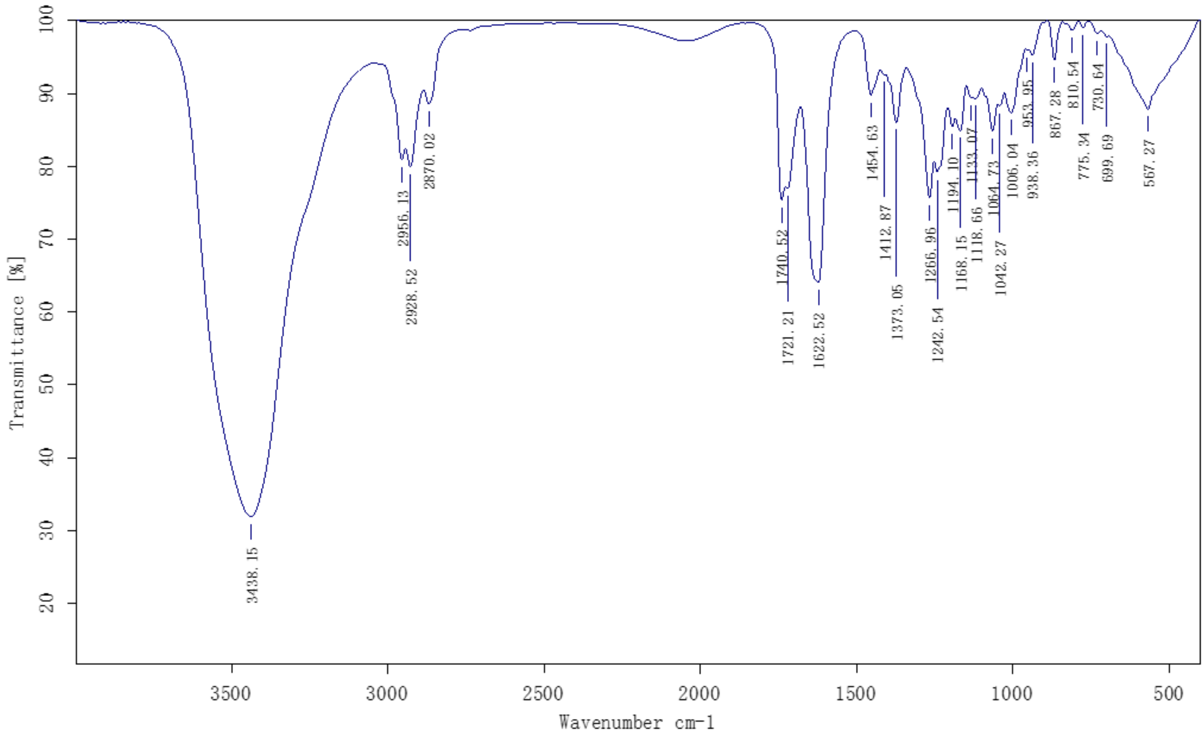

Figure S48. IR spectrum of 5.

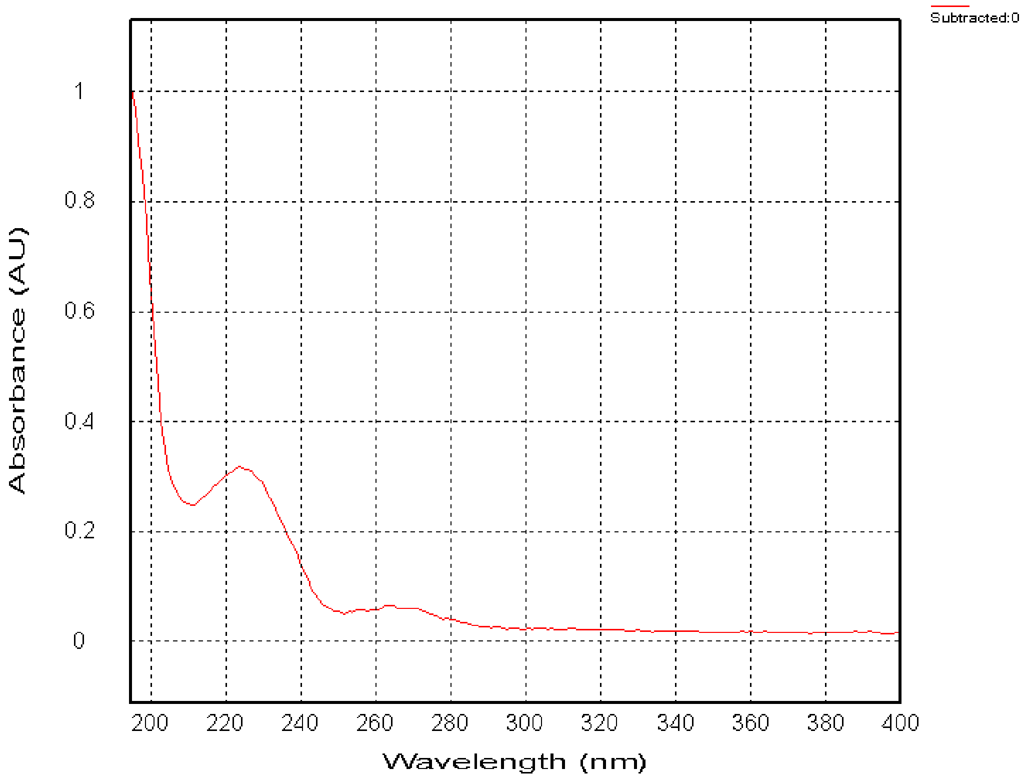

Figure S49. UV spectrum of 5.

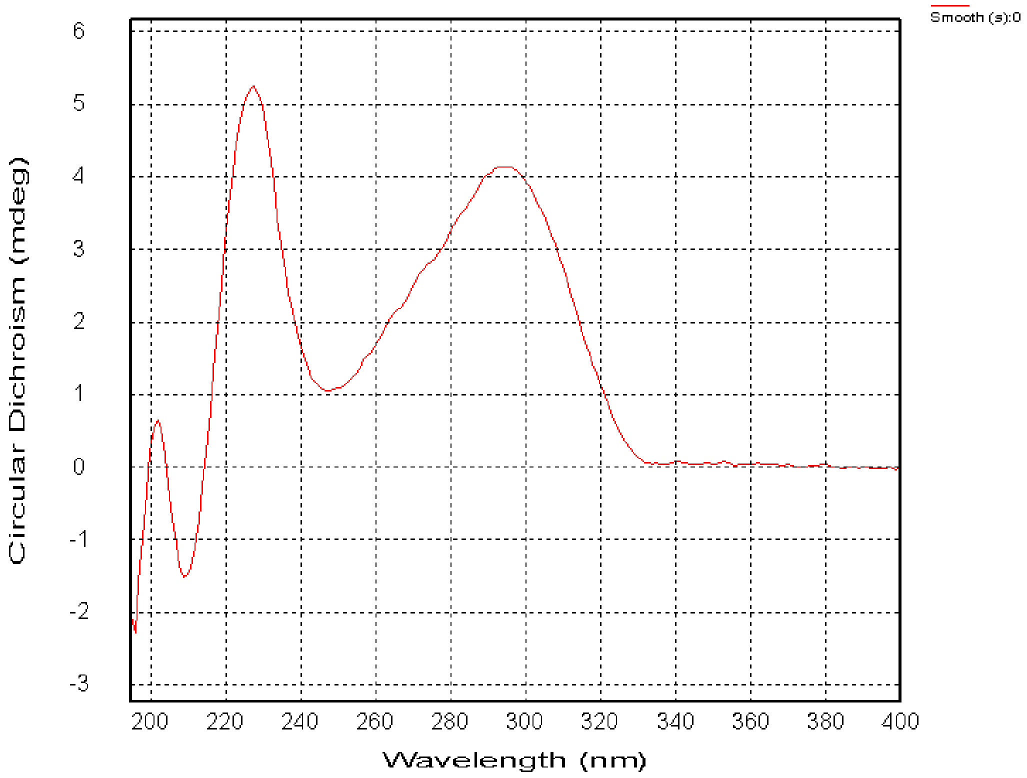

Figure S50. CD spectrum of 5.

Original spectroscopic data of known compounds

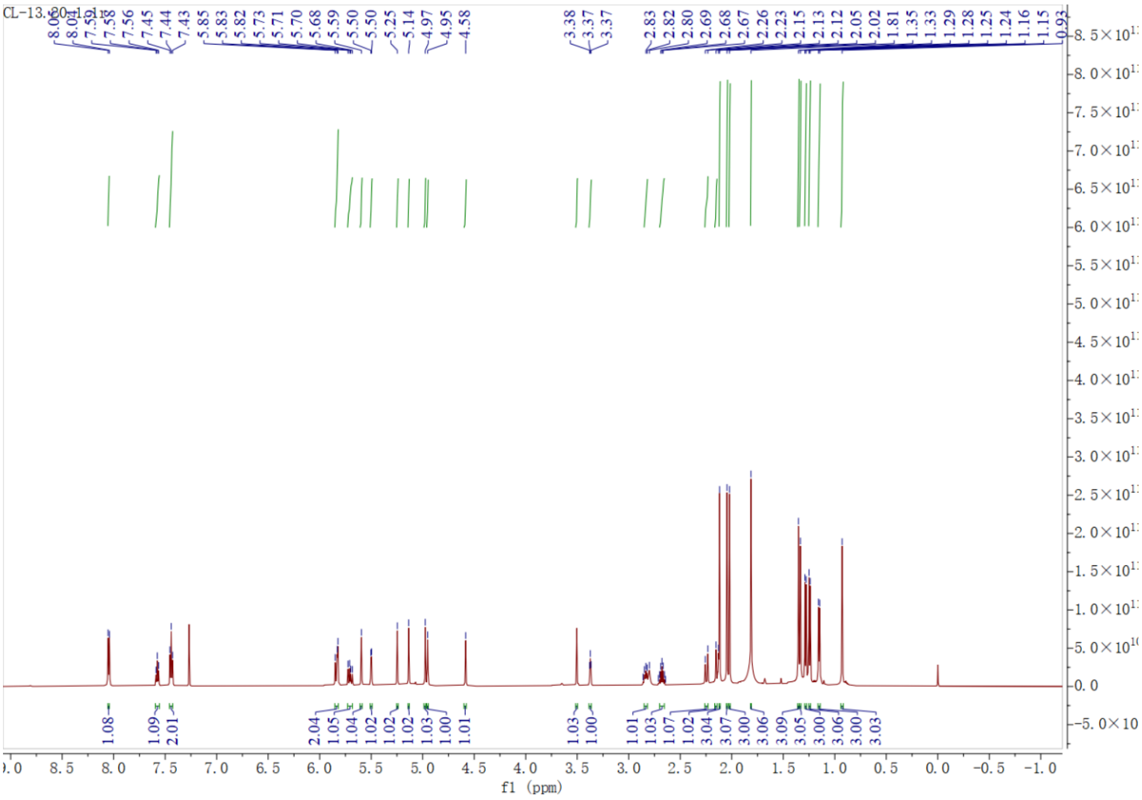

Figure S51. ^{1}**H NMR (400 MHz) spectrum of 6 in CDCl**$_3$**.**

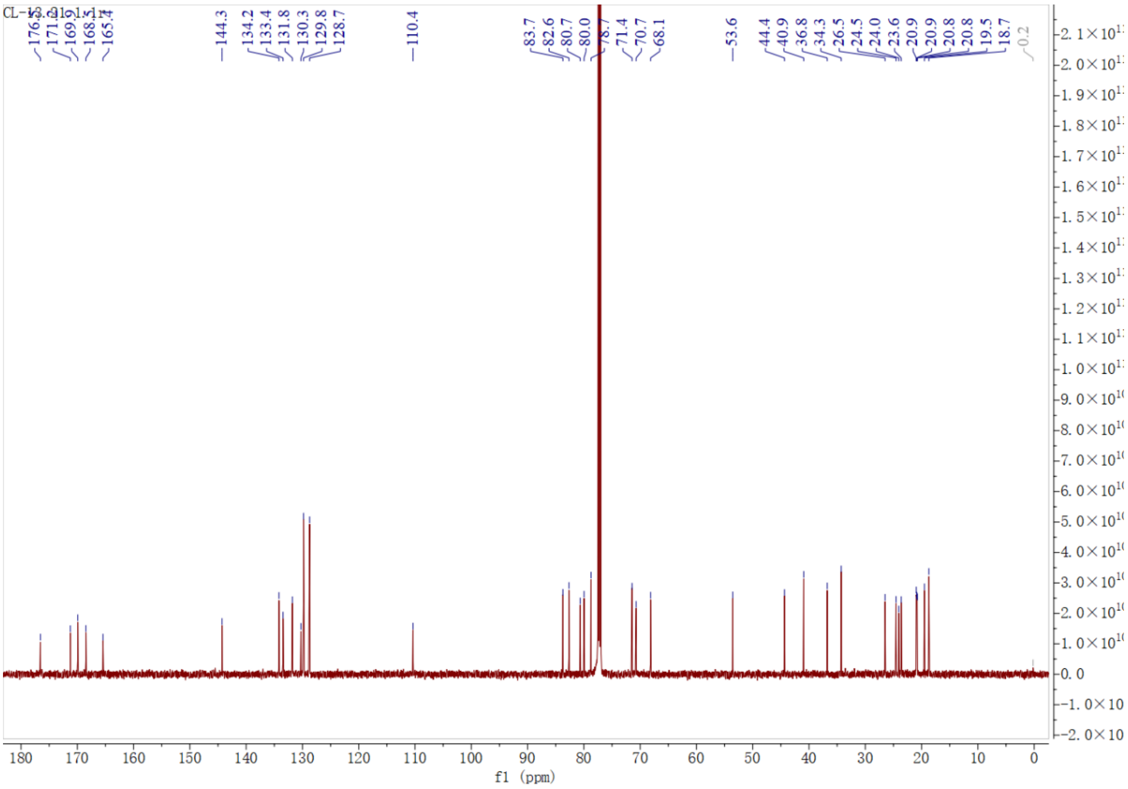

Figure S52. ^{13}**C NMR (100 MHz) spectrum of 6 in** $CDCl_3$**.**

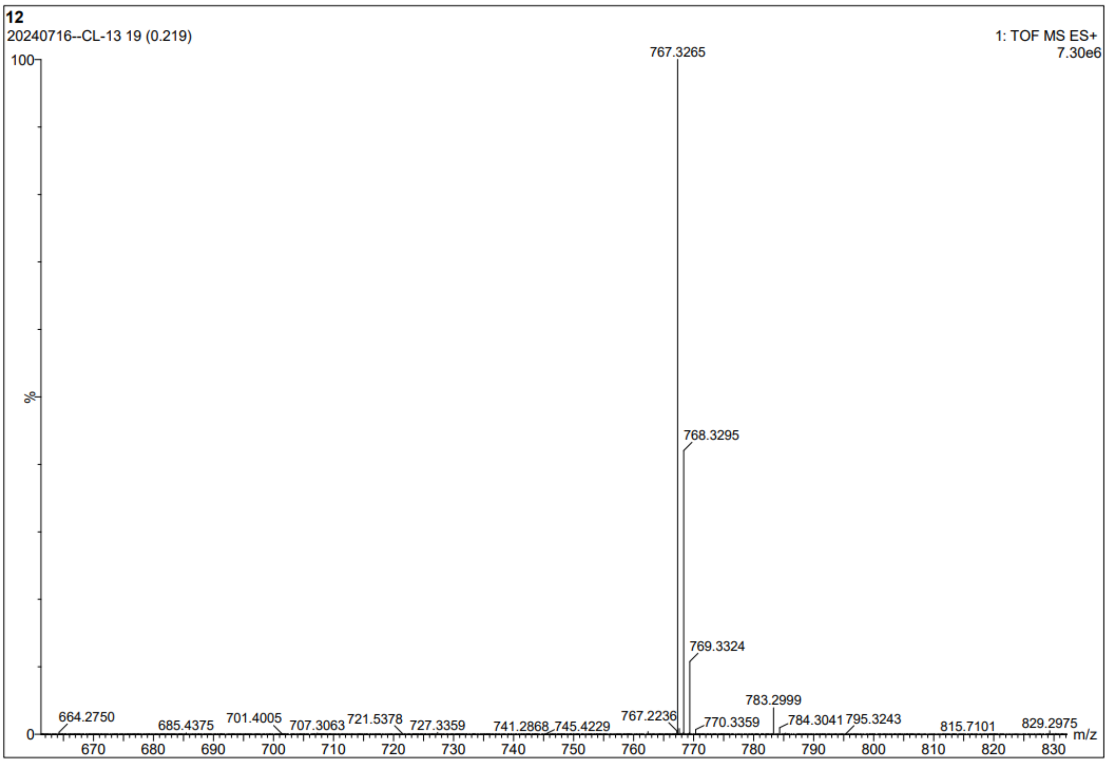

Figure S53. ESIMS spectrum of 6.

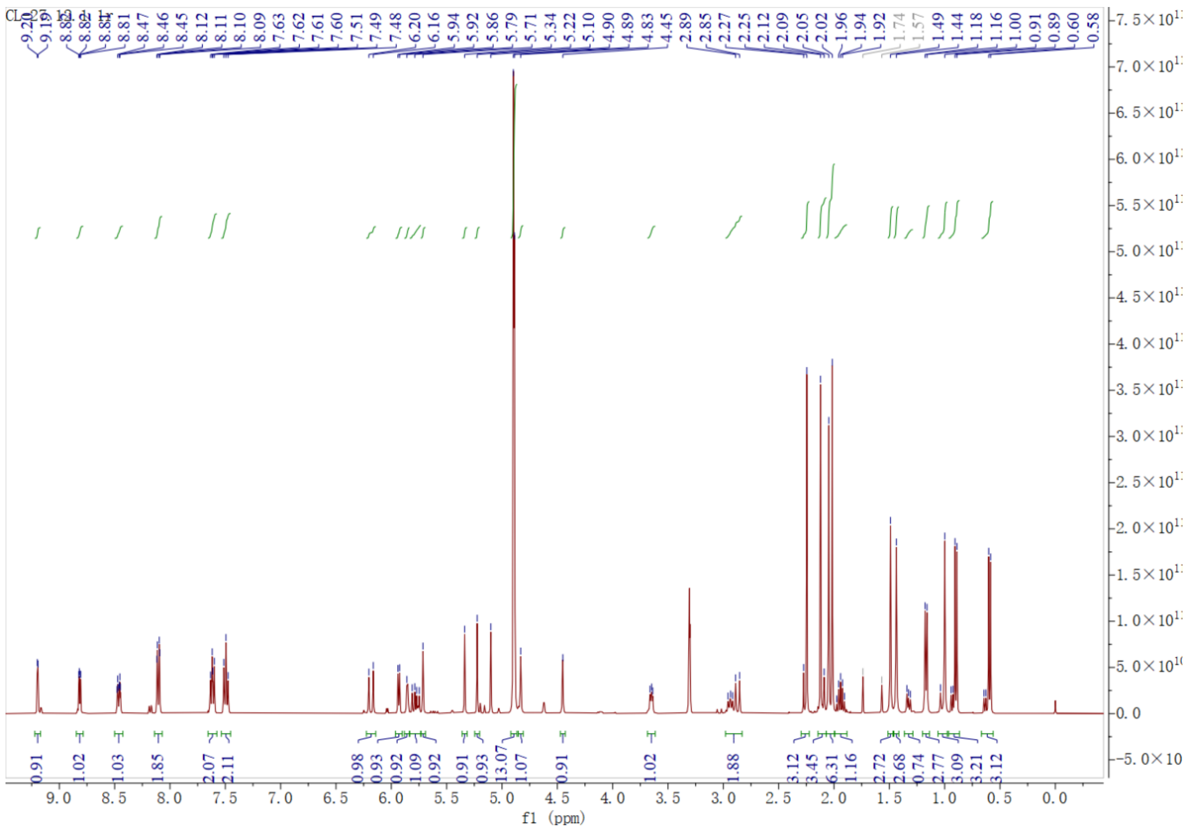

Figure S54. ^{1}H NMR (400 MHz) spectrum of 7 in CD_3OD.

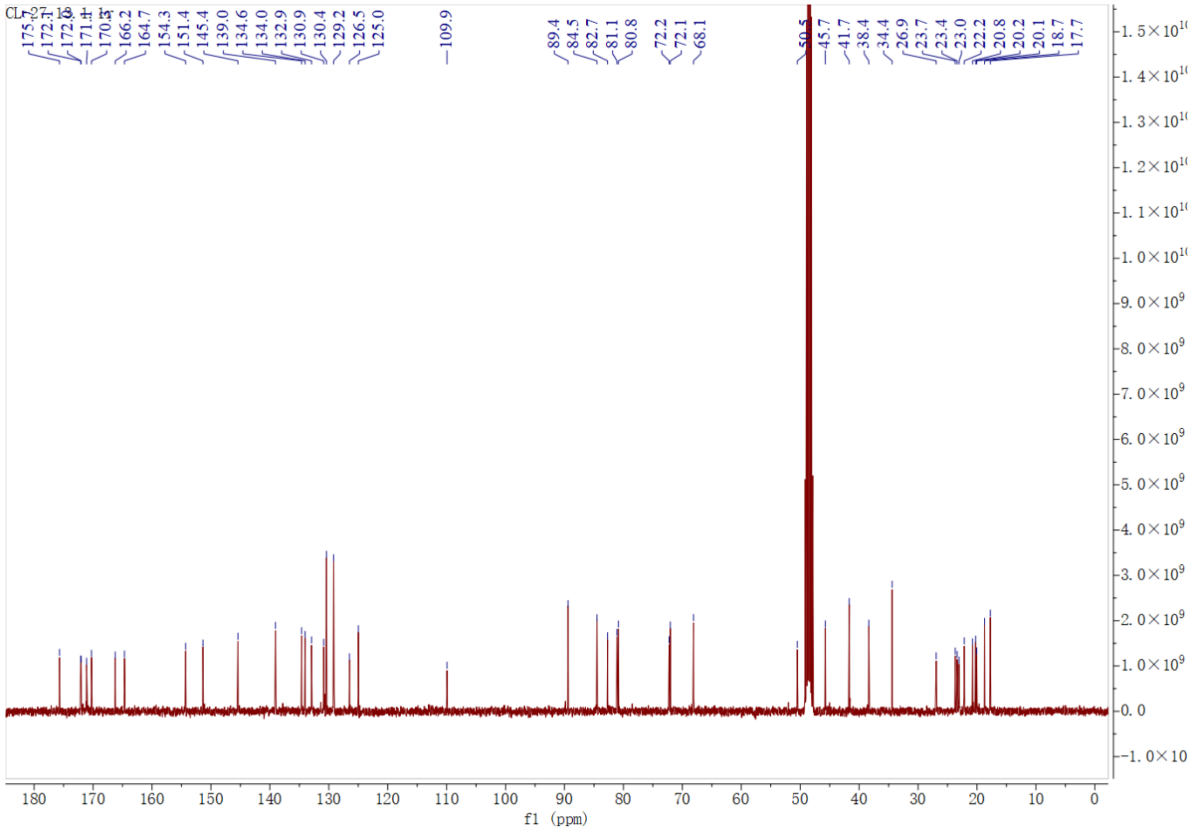

Figure S55. ^{13}C NMR (100 MHz) spectrum of 7 in CD_3OD.

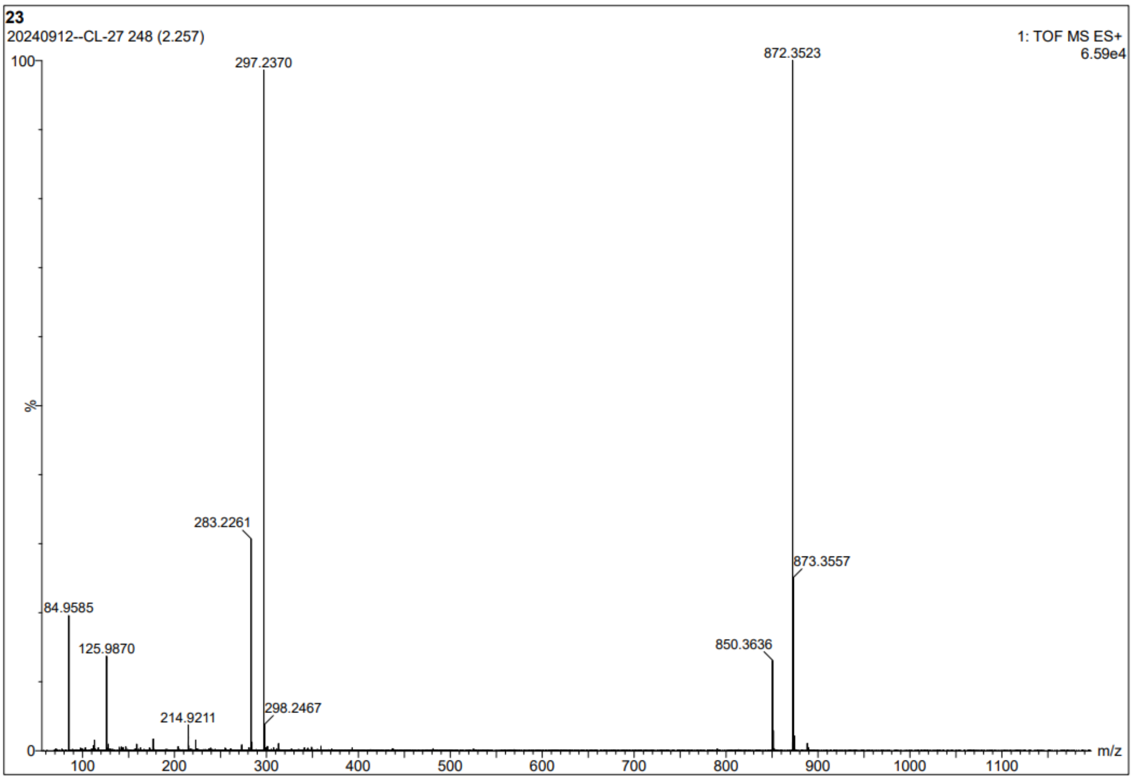

Figure S56. ESIMS spectrum of 7.

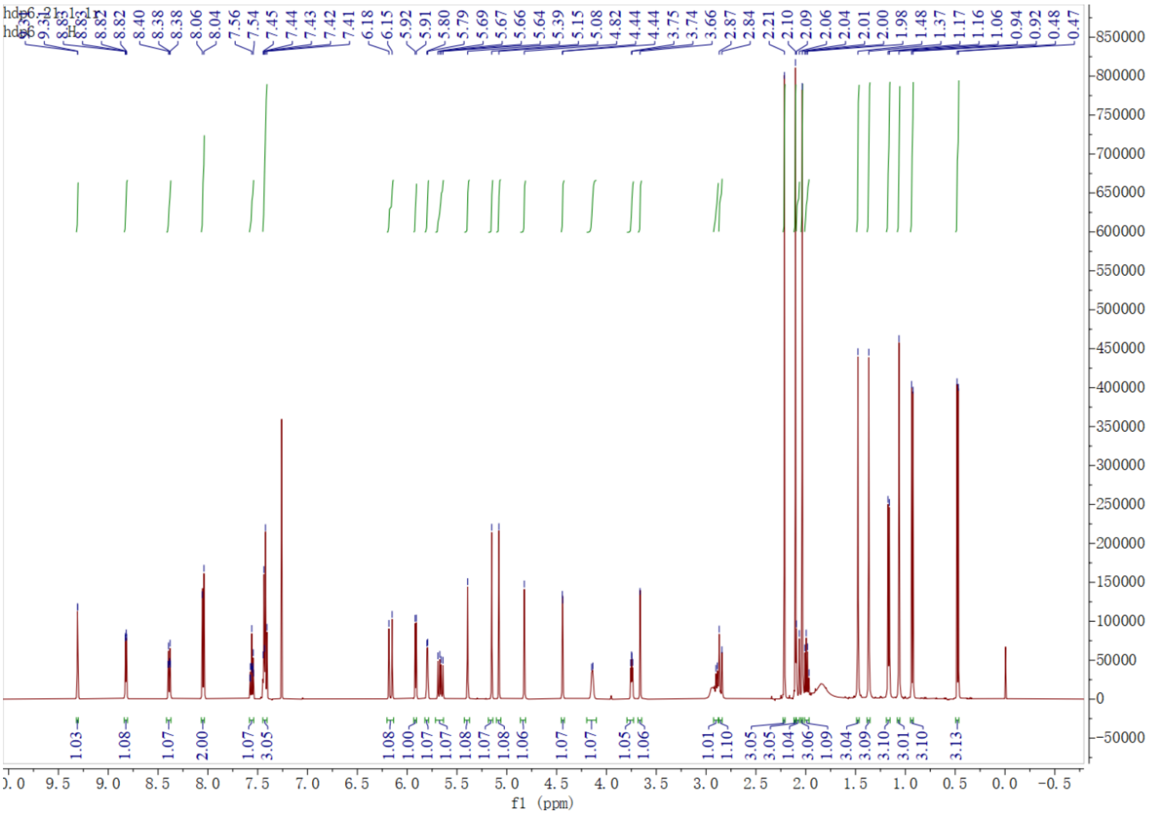

Figure S57. ^{1}H NMR (400 MHz) spectrum of 8 in $CDCl_3$.

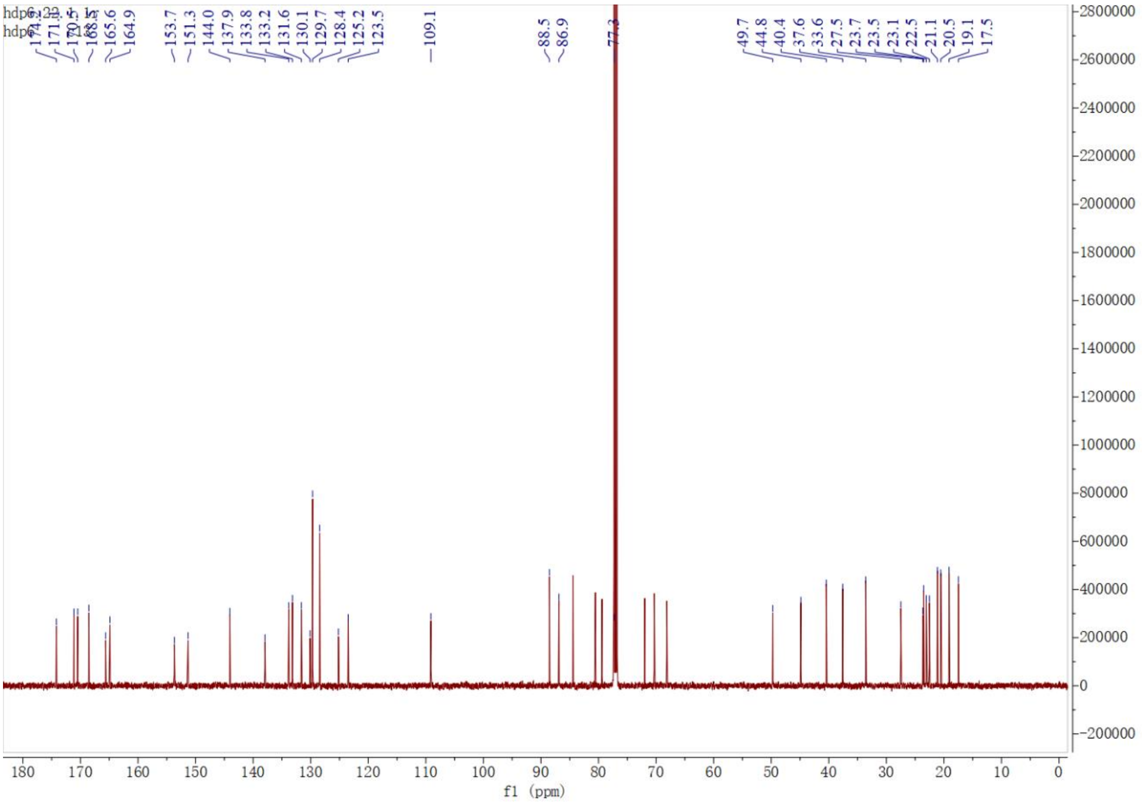

Figure S58. ^{13}C NMR (100 MHz) spectrum of 8 in $CDCl_3$.

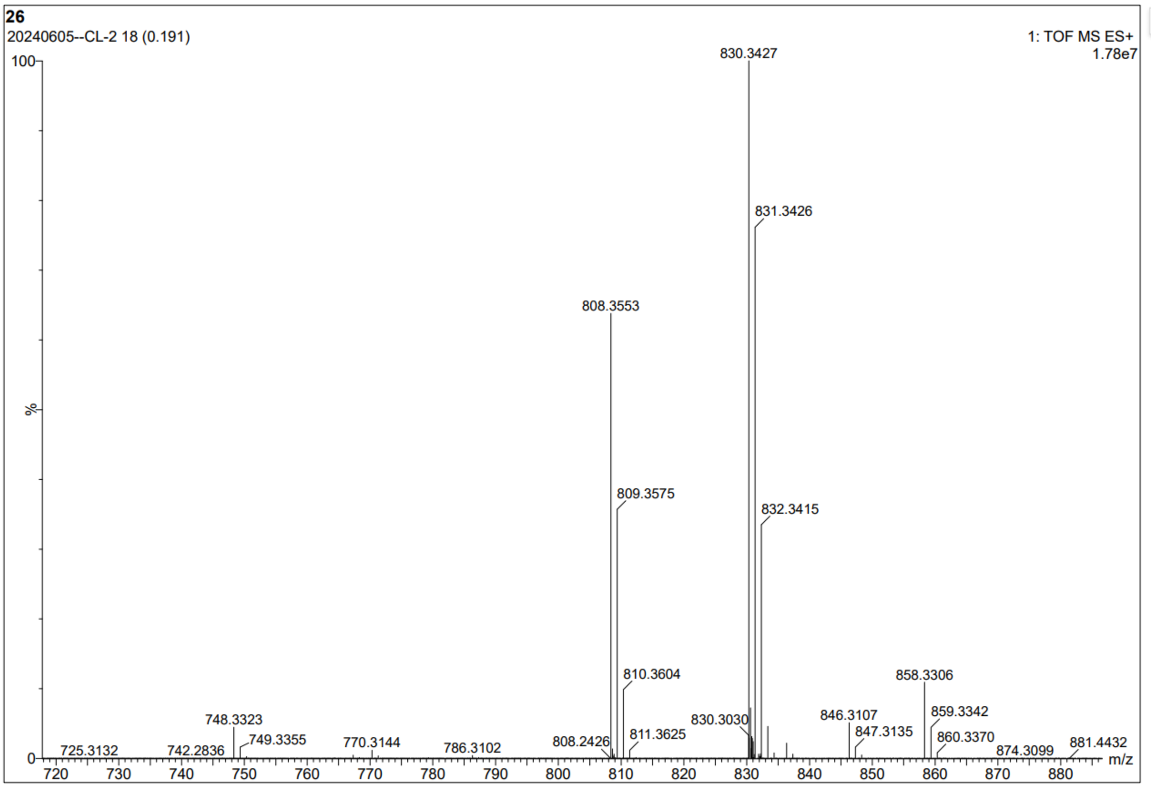

Figure S59. ESIMS spectrum of 8.

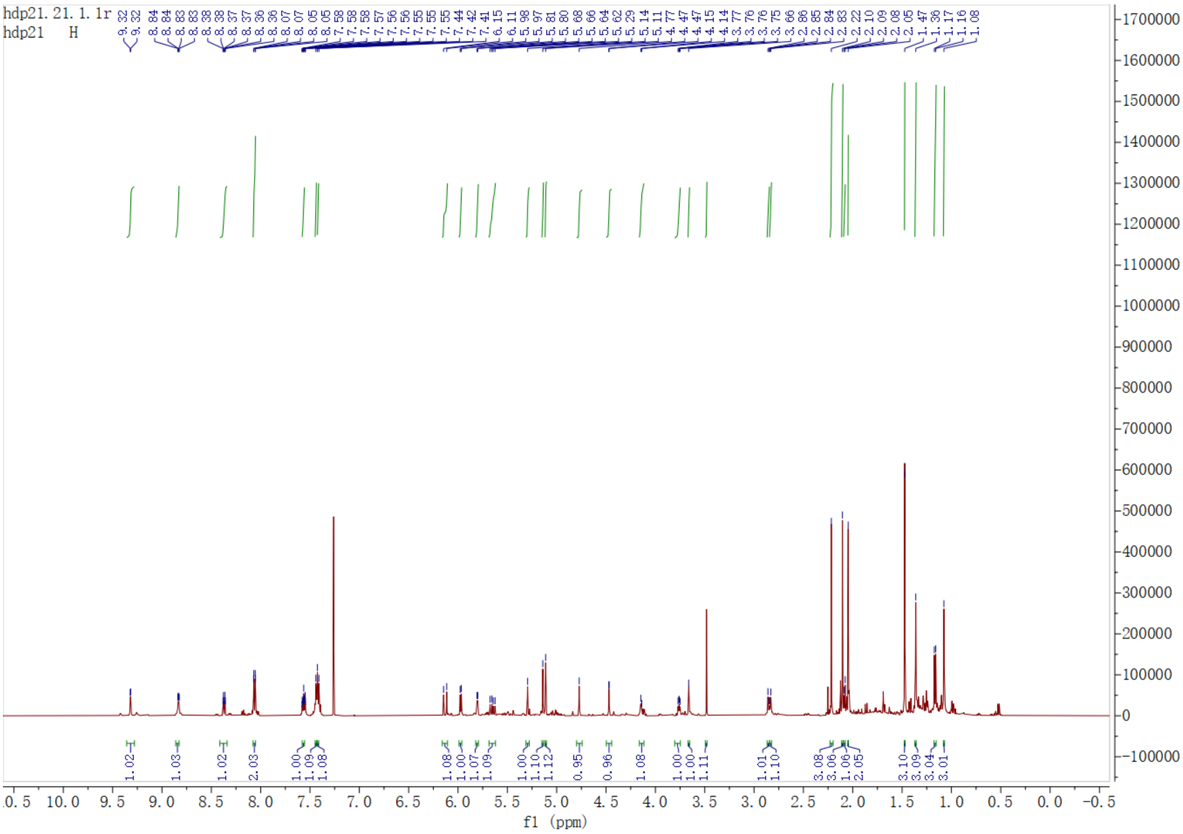

Figure S60. ^{1}**H NMR (400 MHz) spectrum of 9 in** $CDCl_3$**.**

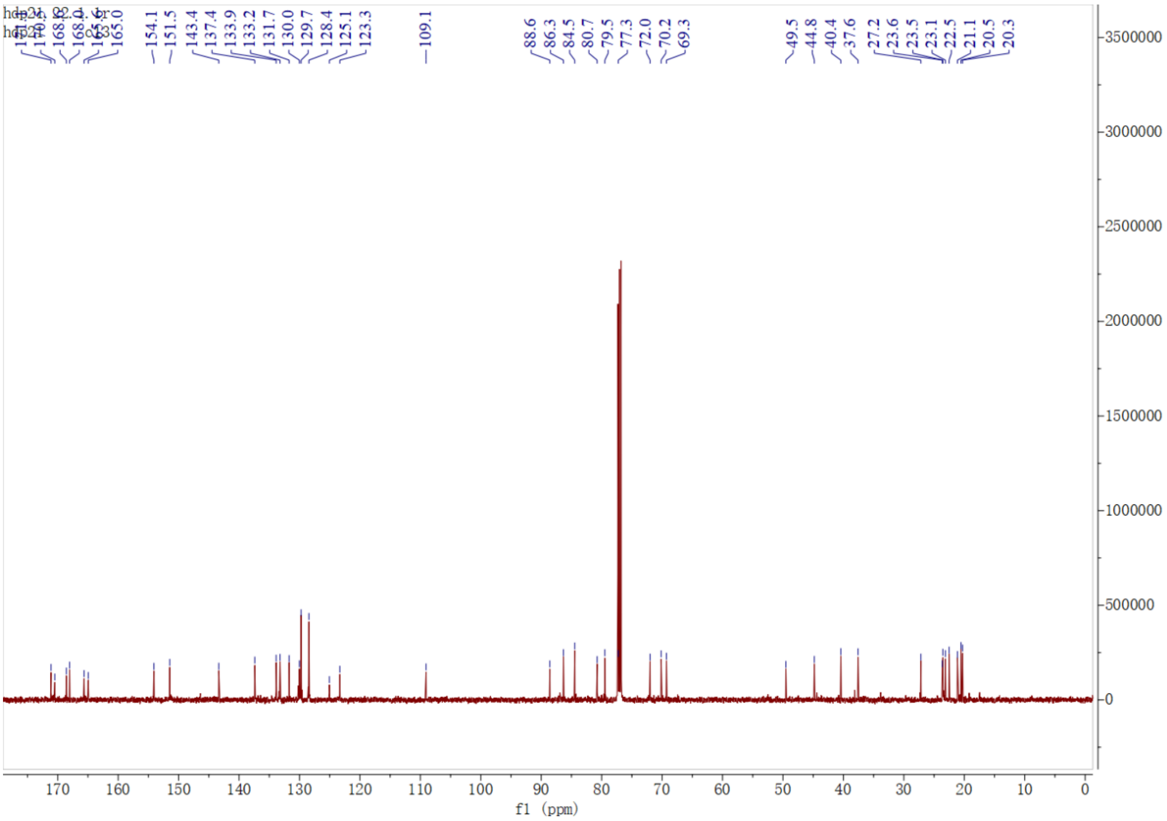

Figure S61. ^{13}**C NMR (100 MHz) spectrum of 9 in** $CDCl_3$**.**

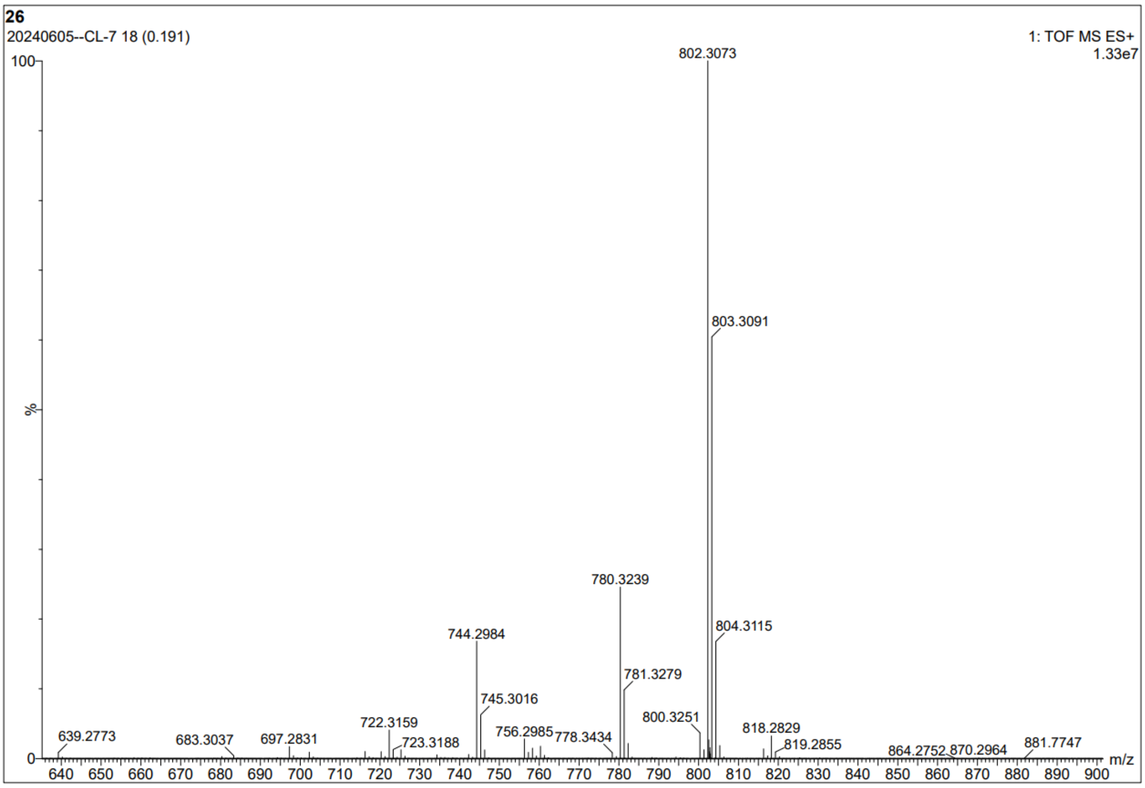

Figure S62. ESIMS spectrum of 9.

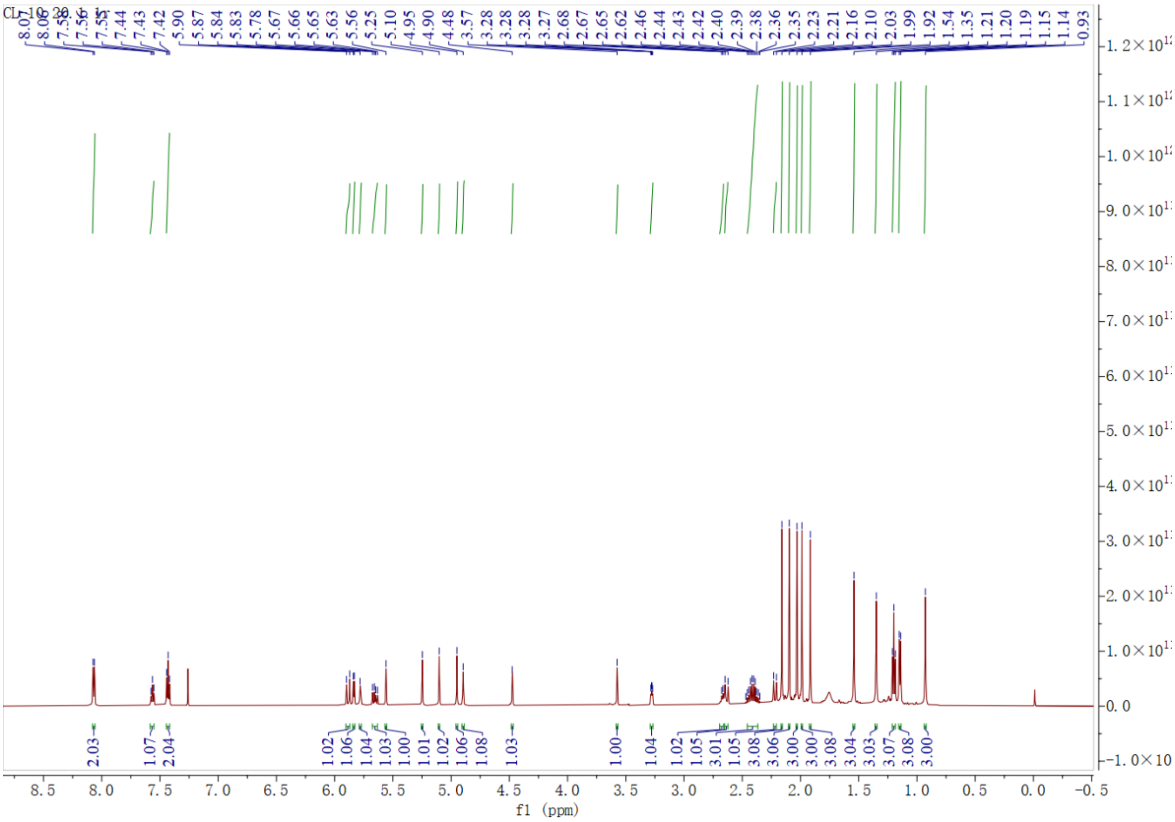

Figure S63. ^{1}H NMR (400 MHz) spectrum of 10 in $CDCl_3$.

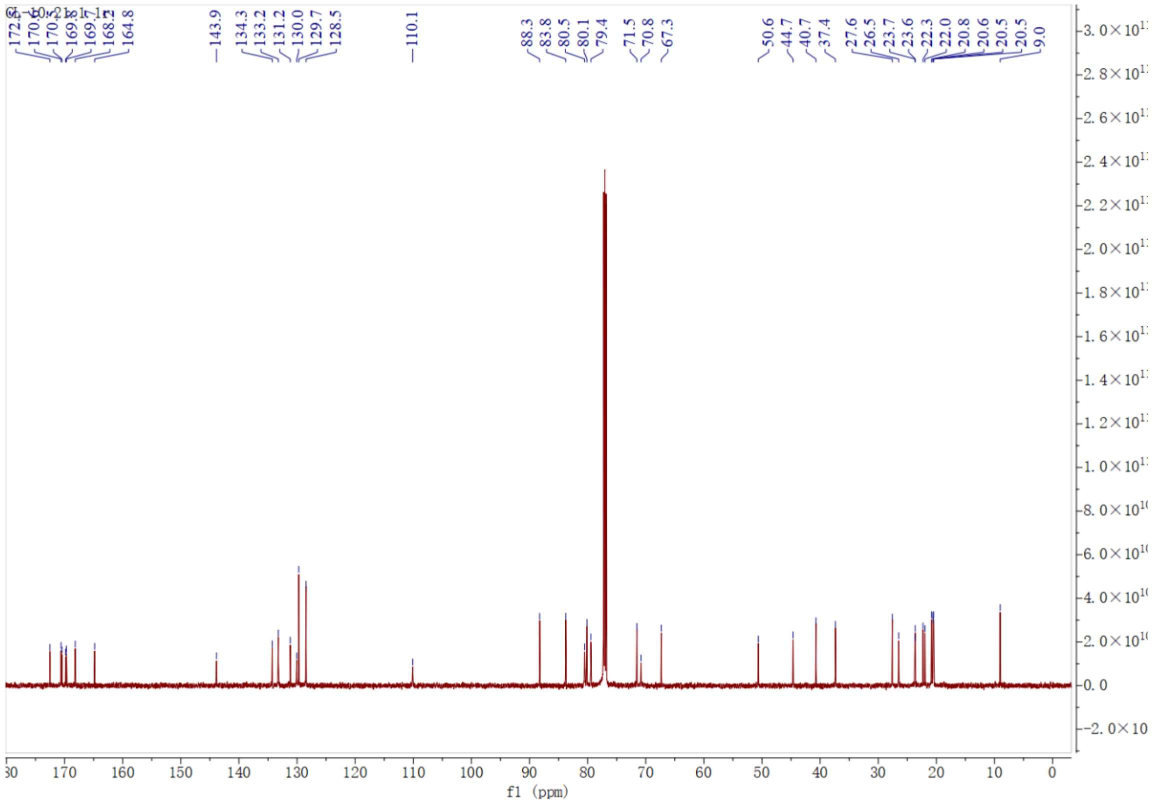

Figure S64. ^{13}**C NMR (100 MHz) spectrum of 10 in** $CDCl_3$**.**

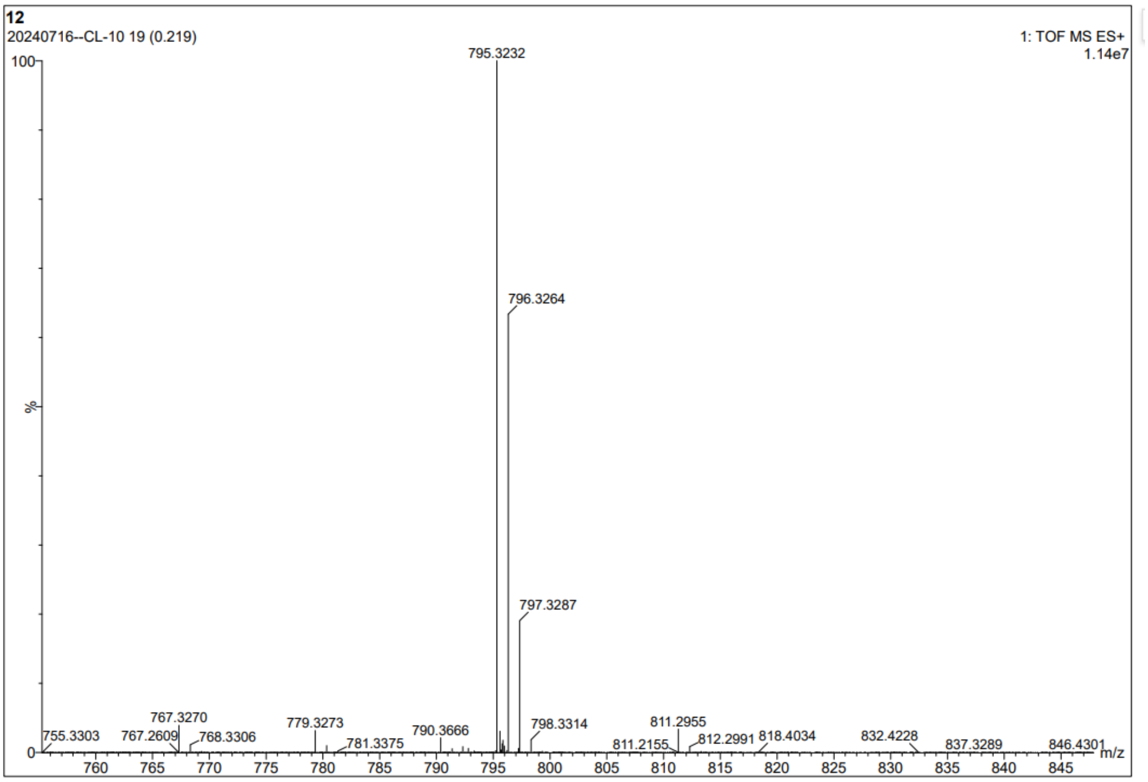

Figure S65. ESIMS spectrum of 10.

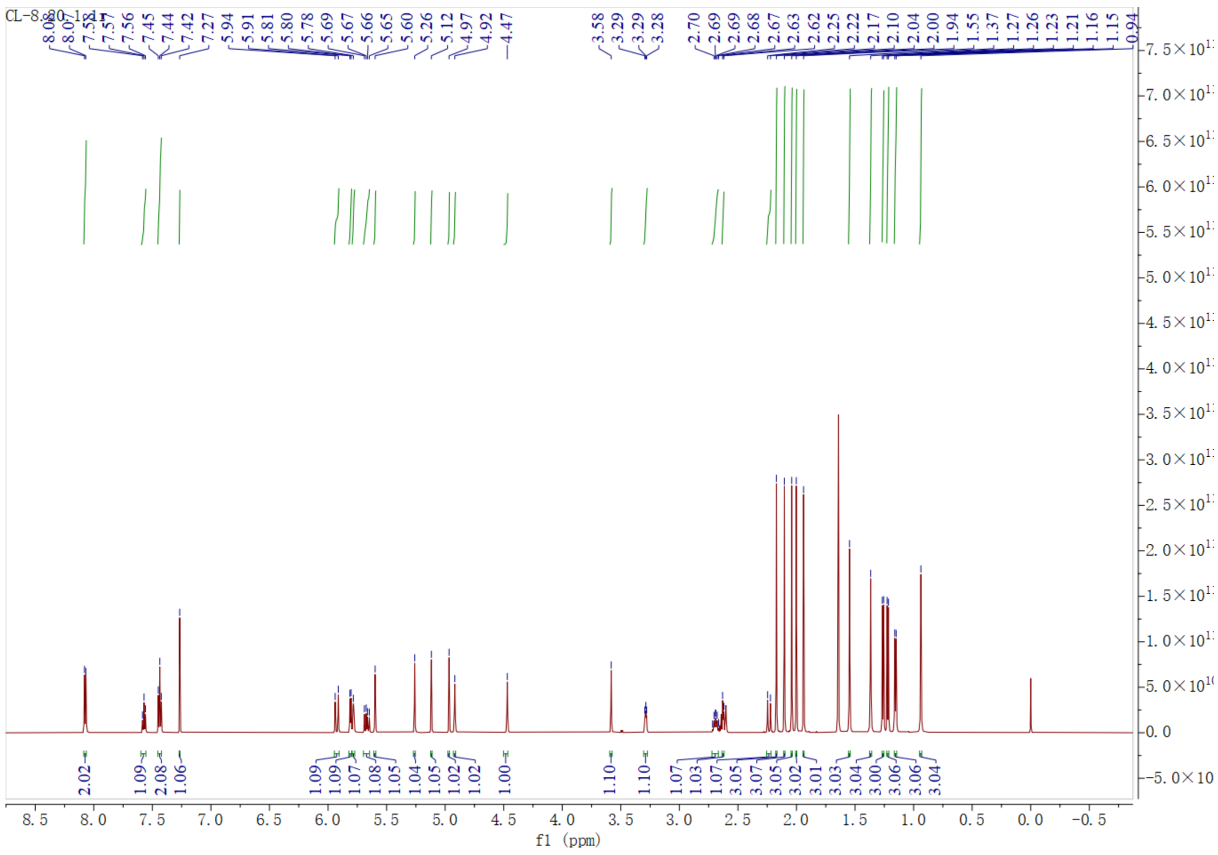

Figure S66. ^{1}H NMR (400 MHz) spectrum of 11 in $CDCl_3$.

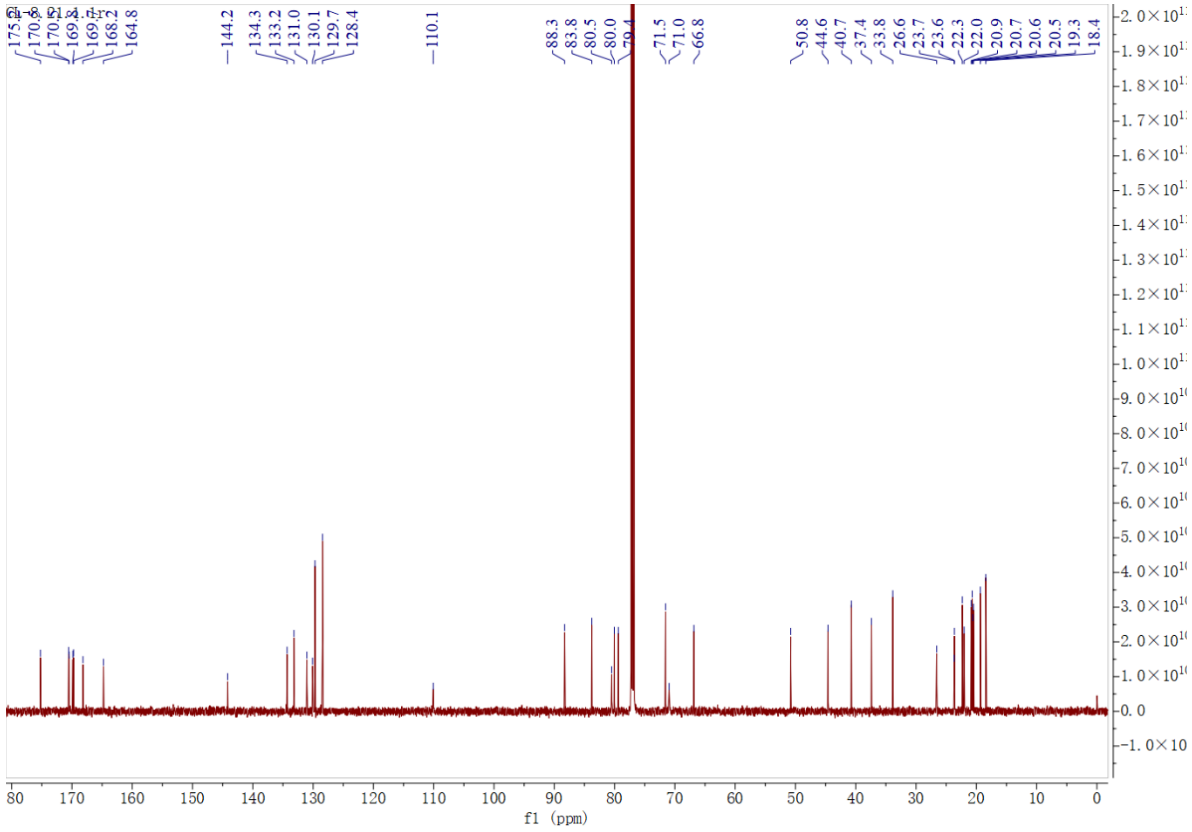

Figure S67. ^{13}C NMR (100 MHz) spectrum of 11 in $CDCl_3$.

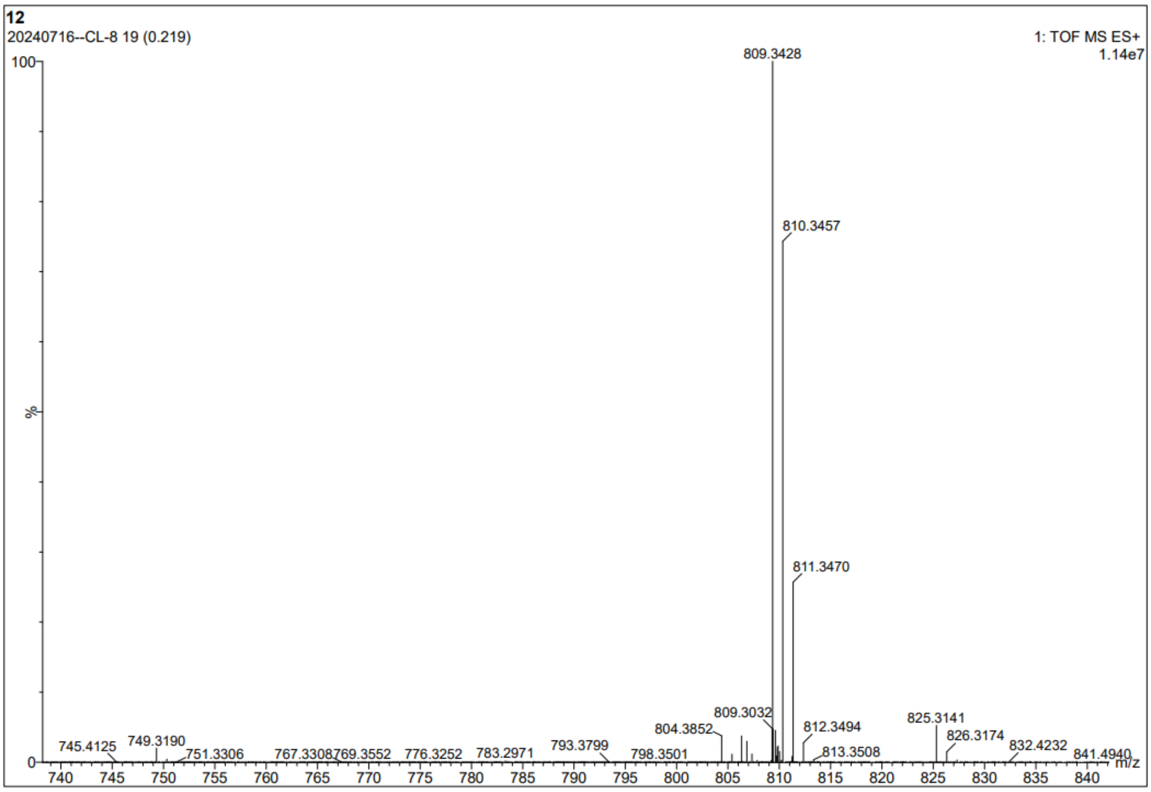

Figure S68. ESIMS spectrum of 11.

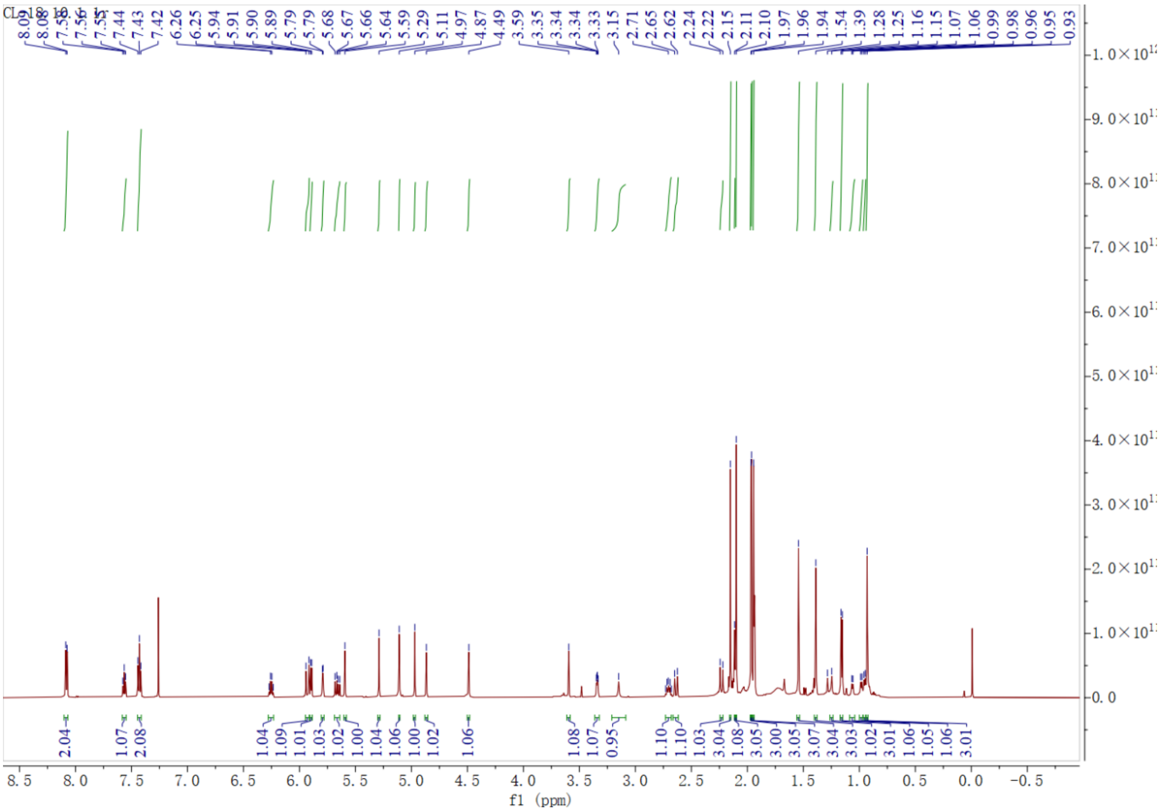

Figure S69. ^{1}H NMR (400 MHz) spectrum of 12 in $CDCl_3$.

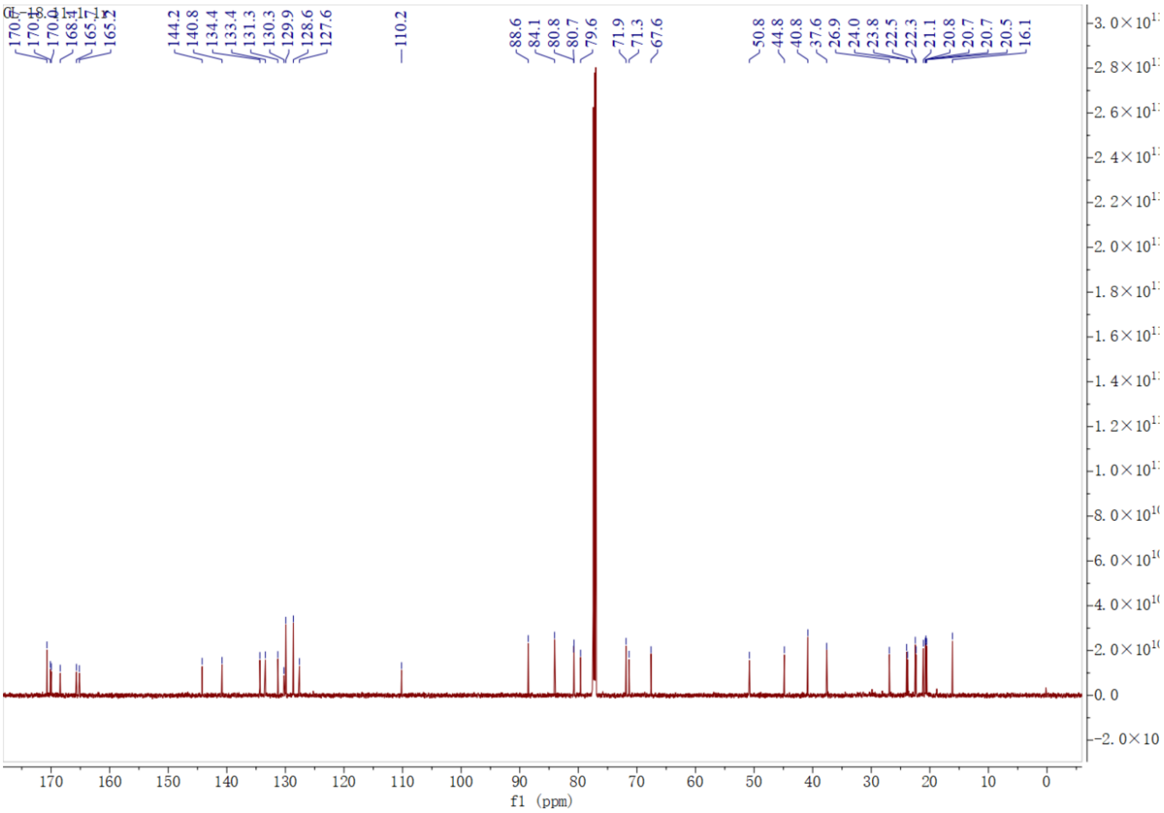

Figure S70. ^{13}C NMR (100 MHz) spectrum of 12 in $CDCl_3$.

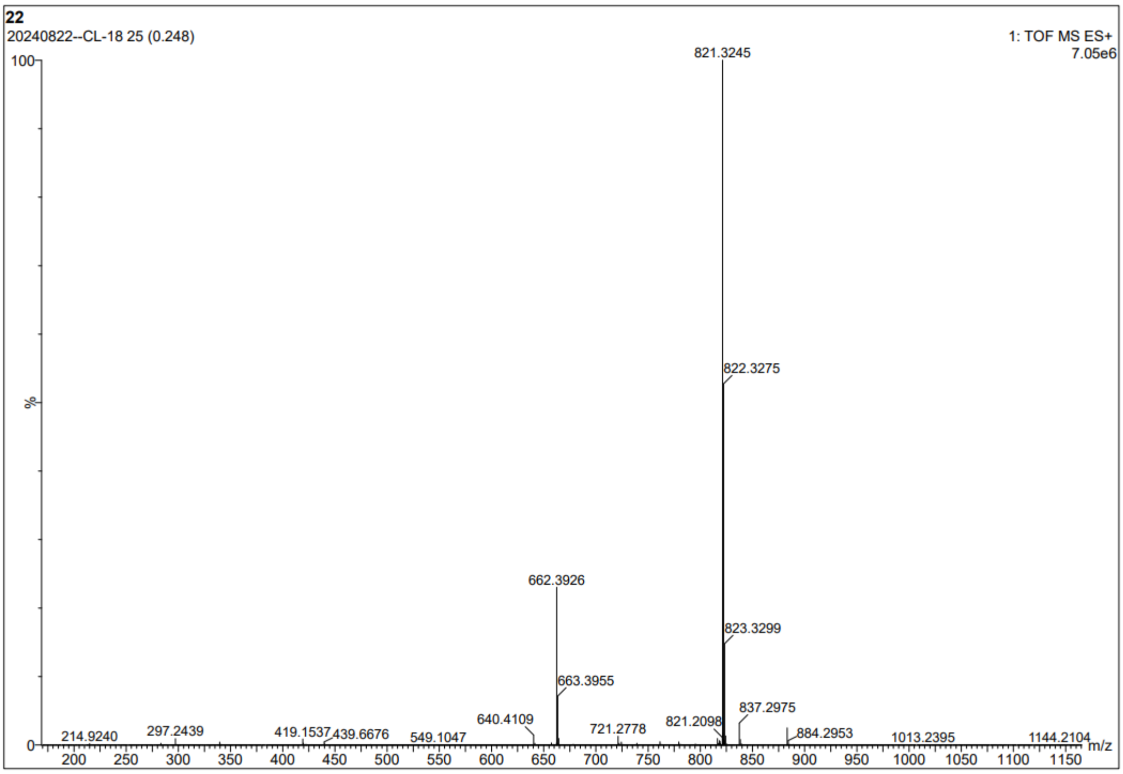

Figure S71. ESIMS spectrum of 12.

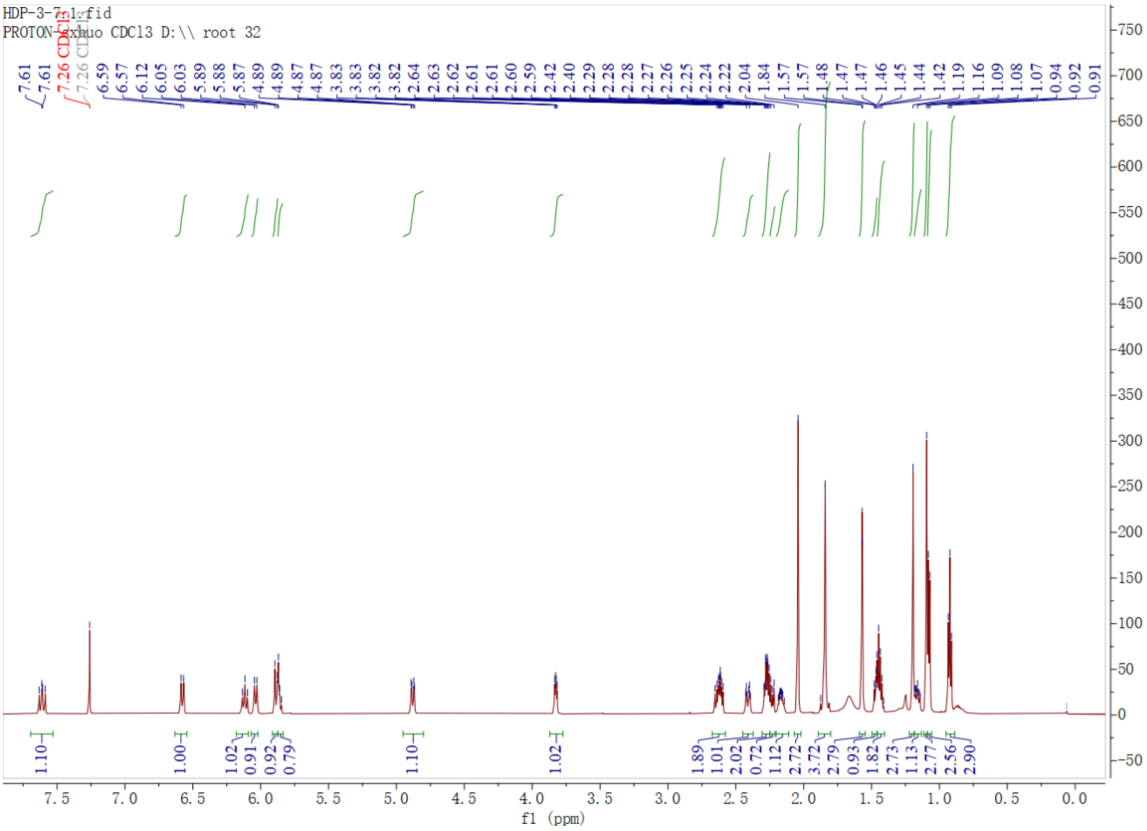

Figure S72. ^{1}H **NMR (600 MHz) spectrum of 13 in** $CDCl_3$.

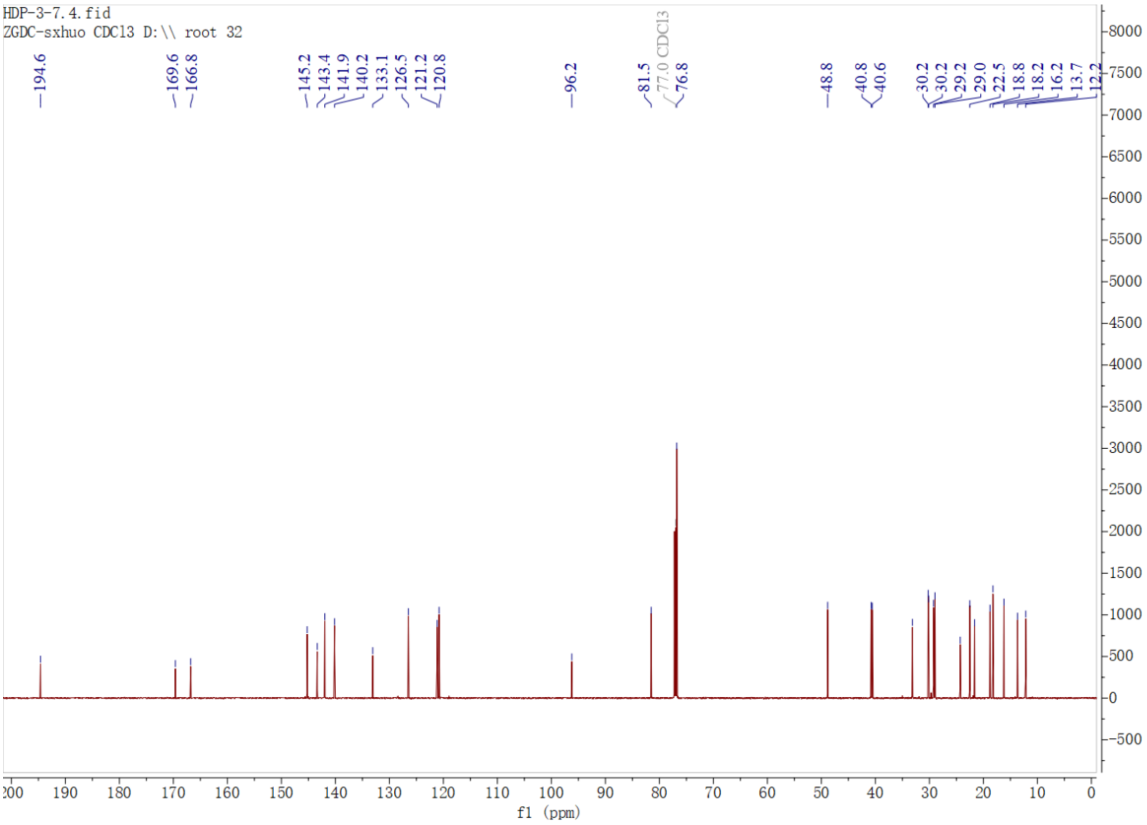

Figure S73. ^{13}C **NMR (150 MHz) spectrum of 13 in** $CDCl_3$.

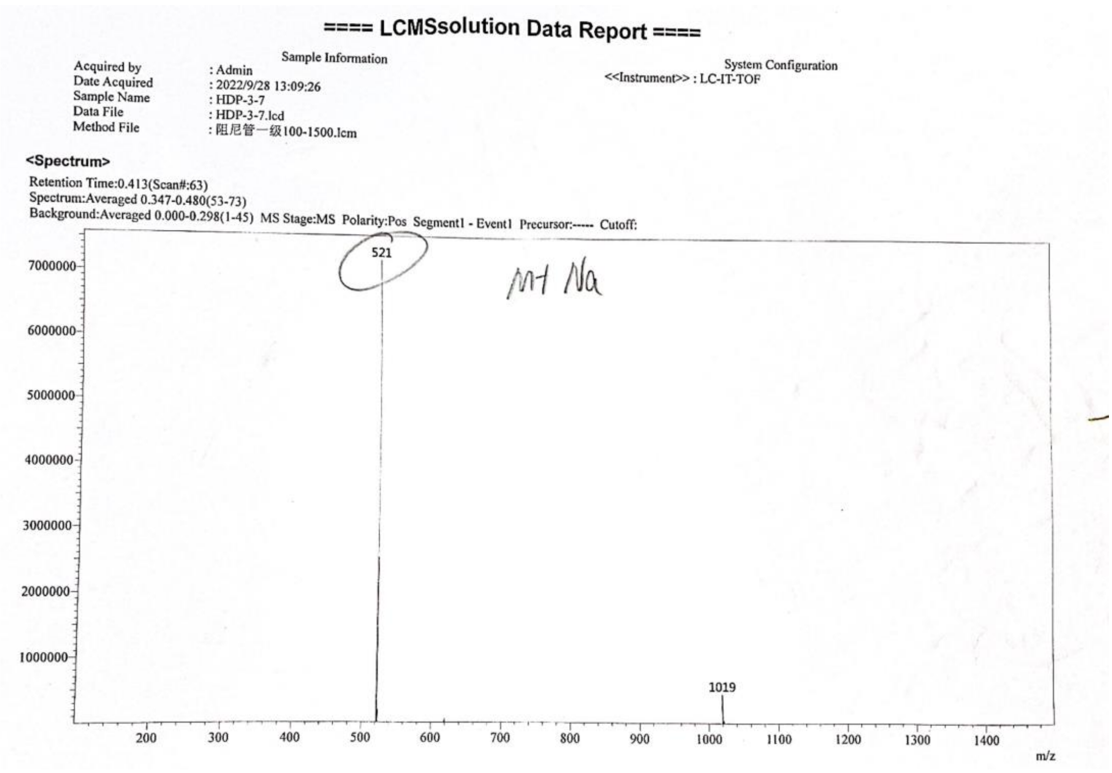

Figure S74. ESIMS spectrum of 13.
